# Historical reconstruction of the population dynamics of southern right whales in the southwestern Atlantic Ocean

**DOI:** 10.1038/s41598-022-07370-6

**Published:** 2022-02-28

**Authors:** M. A. Romero, M. A. Coscarella, G. D. Adams, J. C. Pedraza, R. A. González, E. A. Crespo

**Affiliations:** 1 Escuela Superior de Ciencias Marinas - Universidad Nacional del Comahue, San Martín 247, 8520 San Antonio Oeste, Río Negro Argentina; 2 Centro de Investigación Aplicada y Transferencia Tecnológica en Recursos Marinos “Almirante Storni” (CIMAS). Consejo Nacional de Investigaciones Científicas y Técnicas (CONICET), Güemes 1030, 8520 San Antonio Oeste, Río Negro Argentina; 3 Laboratorio de Mamíferos Marinos, Centro Para El Estudio de Sistemas Marinos (CESIMAR) CENPAT-CONICET, Blvd. Brown 2915, 9120 Puerto Madryn, Chubut Argentina; 4 Universidad Nacional de La Patagonia San Juan Bosco, Blvd. Brown 3051, 9120 Puerto Madryn, Chubut Argentina; 5 School of Aquatic and Fishery Science, University of Washington, 1122 NE Boat St, Seattle, WA 355020 USA; 6 Ciclo Básico Común - Área Matemática - Universidad de Buenos Aires. Ciudad Universitaria, Nuñez, Av. Cantilo S/N, Pab 3, Subsuelo, 1428 Buenos Aires, Argentina

**Keywords:** Zoology, Ecology, Ecological modelling, Population dynamics

## Abstract

Understanding the recovery of whale populations is critical for developing population-management and conservation strategies. The southern right whale (SRW) *Eubalena australis* was one of the baleen whale species that has experienced centuries of exploitation. We assess here for the first time the population dynamics of the SRW from the southwestern Atlantic Ocean at the regional level to measure numerically the effect of whaling and estimate the population trend and recovery level after depletion. We reconstructed the catch history of whaling for the period 1670–1973 by an extensive review of different literature sources and developed a Bayesian state-space model to estimate the demographic parameters. The population trajectory indicated that the pre-exploitation abundance was close to 58,000 individuals (median = 58,212; 95% CI = 33,329–100,920). The abundance dropped to its lowest abundance levels in the 1830s when fewer than 2,000 individuals remained. The current median population abundance was estimated at 4,742 whales (95% CI = 3,853–6,013), suggesting that the SRW population remains small relative to its pre-exploitation abundance (median depletion *P*_2021_ 8.7%). We estimated that close to 36% of the SRW population visits the waters of the Península Valdés, the main breeding ground, every year. Our results provide insights into the severity of the whaling operation in the southwestern Atlantic along with the population´s response at low densities, thus contributing to understand the observed differences in population trends over the distributional range of the species worldwide.

## Introduction

From the pioneering models for growing populations—such as the classical logistic function of Verhulst and Elton’s focus on population cycles—ecologists and managers have been interested in understanding how populations change over time^1,2^. Analyses of temporal series of animal abundances are of great interest for life history theory, population ecology, and wildlife management through providing estimates of abundance trends or growth rates as well as density-dependence processes^3–5^. Demographic assessment is also an essential step in conservation biology because that evaluation enables one to diagnose the cause of long-term changes in population abundance^6^.

Populations of large vertebrates have undergone numerous threats, including habitat loss, harvesting, climate change and prey depletion, leading to drastic reductions in their abundance^7,8^. In the marine realm, the past large-scale whaling and sealing operations were the main threats for marine mammal´s populations^9,10^. Whales and seals have been pursued historically as prized sources of oil, fur, meat, baleen and ambergris^11,12^. At present, certain populations remain at low abundance levels since the end of commercial whaling, while others have continued to decline or have even become extinct or extirpated. Most, however, have manifested remarkable recoveries after severe depletions^13–15^. Effective conservation actions require a better understanding of the underlying mechanisms that determine the magnitude of the population trends.

The southern right whale (SRW) *Eubalaena australis* (Desmoulins, 1822) was one of the baleen whale species most extensively hunted by commercial whaling in the Southern Hemisphere, having been driven almost to the border of extinction around the mid-nineteenth century^16–20^. Whaling activity started in the early seventeenth century, and was mainly led by American, British, French, Portuguese and Spanish whalers. Despite the whales having been protected by an international agreement since 1935, illegal hunting by the Soviet Union whaling fleet in the 1960s and 1970s killed off half the existing population at the time^20,21^.

Reconstructions of historical trajectories indicated that before whaling over 70,000 SRWs could be found in the 12 wintering grounds^14,19,20,22^. Between the 18th and the mid-nineteenth centuries a conservative estimate suggested that more than 150,000 SRW had been killed, with only around 300 individuals remaining worldwide by the 1920s^19,20,22^. Currently, the SRW populations have increased to roughly 12,000–15,000 individuals, as estimated in 2009 over the species circumpolar distribution^22^.

Despite the positive global trend, SRW populations have evidenced different population growths. Carroll et al.^23^ estimated that the New Zealand SRW population was growing at 7% per annum (95% IC = 5–9%). The South African-Namibian breeding population was likewise thriving with an annual population growth rate of 6.5% (S.E. 0.3%)^24^. The ‘western’ Australian SRW subpopulation evidences a population trend of 5.6% per year (95% CI = 4–7.28%), while the ‘eastern’ subpopulation remains relatively small^25^. In southeastern Australia, the SRW population has increased at a rate of 4.7% (95% IC = 2.3% − 7.3%) per annum between 1996 and 2017^26^. The continued low abundance of the SRW in certain regions, such as in southeast Australia and the Chile-Peru area, was linked to a strong female fidelity to calving grounds^27–29^.

The breeding ground off Península Valdés (42–43°S), Argentina, has been increasing at around 6–7 ± 0.2% annually for 40 years since the beginning of the population dynamics studies in 1970, thus housing the largest aggregations of SRWs in the southwestern Atlantic Ocean^22,30,31^. During the last ten years the rate of increase has declined to almost 3.15%, (95% IC = 0.53–5.75%) indicating a density-dependence process at this breeding ground^32^. A growing breeding ground also exists along the east coast of South America at Santa Catarina State (27–29° S), Brazil^33,34^, while an increasing number of sightings has been reported in Uruguay, farther north from Península Valdés and south of Santa Catarina and the Falkland (Malvinas) Islands^35–41^. All this area, referred to as the ´Brazil Banks´, was intensively exploited between the seventeenth and twentieth centuries by whaling that took a minimum of 30,000 individuals^18,42,43^.

Evidence from different sources suggests that SRWs from the southwestern Atlantic Ocean belong to the same population. The annual growth rate in southern Brazil has been estimated to be higher than expected due to a purely endogenous increase, suggesting that immigration from other wintering grounds, such as the Península Valdés, may be occurring^33^. This interchange of SRWs between both breeding grounds had previously been indicated by photo-ID studies^44^. Recently, satellite track data from animals tagged in northern Patagonia demonstrated substantial displacements from Uruguay to South Georgia (Islas Georgia del Sur) and the Scotia Sea (Mar de Escocia), suggesting that the SRW inhabits vast extensions of the south Atlantic Ocean and visits multiple potential feeding areas each season^45^. This result is also consistent with findings from isotopic analysis^46–48^, genetic studies^28,49,50^ and whaling voyage logbooks that indicate a continuum in recorded catches along the South American Atlantic coastline^16,21,51^.

The current international management recognizes this population to be of a single stock, and therefore emphasizes the urgency of integrated conservation actions along the east coast of South America. A call was issued to achieve regional-scale estimates of demographic parameters in order to fully understand the dynamics and recovery rates of the SRW population from the southwest Atlantic^52^. The lack of an extensive pre-modern whaling dataset, however, has severely limited our ability to conduct regional assessments of the SRW commercial whaling in the past so as to develop population trajectories for estimating pre-exploitation baselines and current recovery levels.

Therefore, in the work reported here we have assessed for the first time the population dynamics of the SRW from the southwestern Atlantic Ocean at the regional level in order to measure numerically the effect of whaling and estimate the population trend and recovery level after depletion. We reconstructed the catch history of whaling for the period 1670–1973 by an extensive review of different literatures sources, and then used this information to estimate the current and pre-exploited population abundance, using a Bayesian state-space surplus production model. The results from this analysis enhance our understanding of the response of the species to past exploitation and thus can assist in quantifying baselines for conservation objectives to ensure effective resource management.

## Materials and methods

### Annual whaling data

Whalers termed the *Eubalaena* spp. as the ´right´ whale to kill for their accessibility in nearshore habitats, relatively slow-moving and their tendency to float when dead. Moreover, their capture yielded long baleen plates and copious oil^19^. The first whalers used the Basque-shore whaling technique which was exported to the South Atlantic by 1602^53^. This basic technique was first appropriated by European nations but then spread all the way from Salvador de Bahia to Imbituba, Brazil^54^. Following the decline in the Basque-style era at the end of the seventeenth century, the American shore and pelagic whaling methods prevailed during approximately two centuries^55^. Offshore whaling, although termed American (“Yankee”), was in fact conducted by many nations, plundering the South Atlantic for the remnants of the SRW population^51,56,57^. By the start of modern whaling at the beginning of the twentieth century, the species had already become rare^58^.

**Figure 1:**
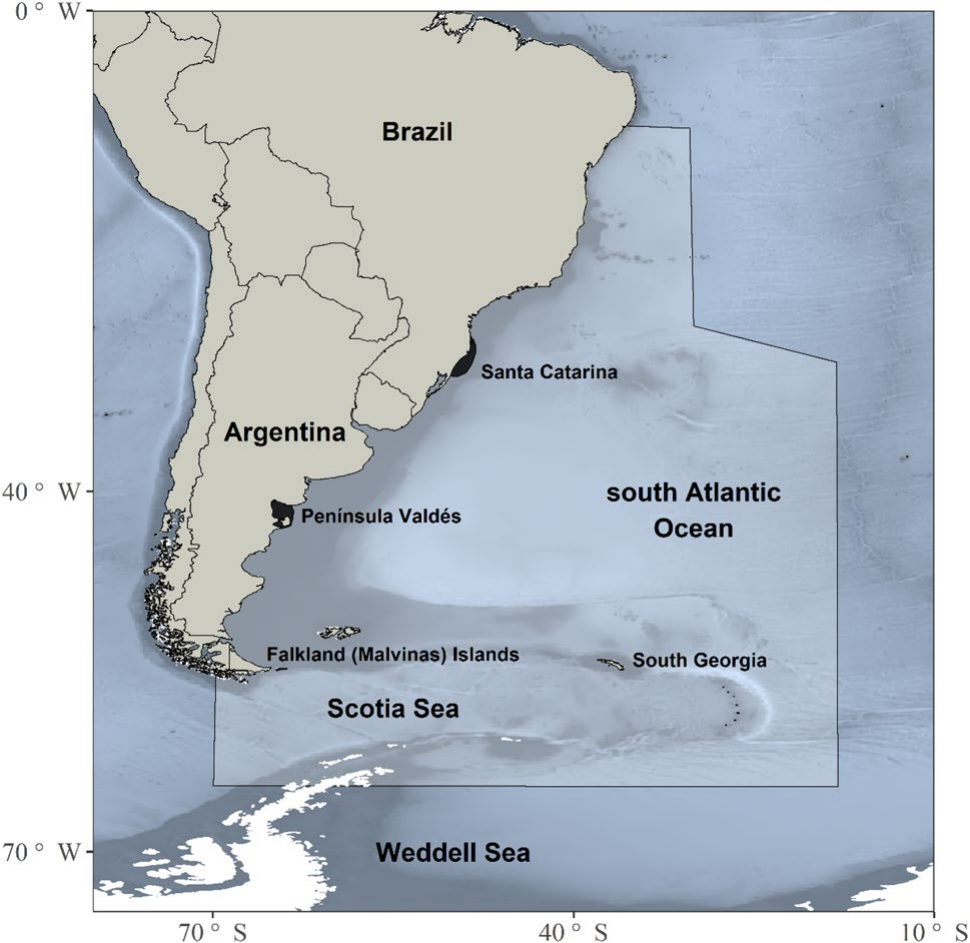
Population range of the southern right whale *Eubalaena australis* over the southwestern Atlantic Ocean, indicating the two breeding grounds (Santa Catarina and Península Valdés; black swathes). Polygon indicates the main area of the whaling operations. Map created with R^76^ through the use of marmap^115^ and ggplot2^116^ packages.

The catch history of the SRW from the southwestern Atlantic Ocean (Table S1, Fig. 1) was reconstructed by combining information from the following sources:

(i) *An extensive review of published articles, books, and theses*. Partial catch reconstructions are available for different fleets, but vary in completeness and in the information tabulated. The whaling operation along the coast of Brazil was initially reviewed by Ellis^59,60^ and subsequently by Palazzo & Carter^61^. Du Pasquier^42^ provided a list of whaling voyages for French whaling vessels, including a detailed description of the whales killed. The whaling activities of American, British, French, Spanish and Portuguese fleets up to the middle of the eighteenth century was reviewed by Richards^18^. These data were assigned for the period 1772–1812. Additional data were compiled from^62–67^.
(ii) *The database available on the Whaling History website* (www.whalinghistory.org). This dataset contains information about the American offshore whaling, the British Southern Whale Fishery, and the French whaling. The American Offshore Whaling Logbook database^68^ was extracted from the original whaling logbooks compiled by Lt. Cmdr. Matthew Fontaine Maury in the 1850s, by Charles Haskins Townsend in the 1930s and by the Census of Marine Life project (CoML, www.coml.org). This database contains records providing information on the whales seen and captured along with the date and the location of the vessel. These records were plotted and filtered to select all those included in the study area. The Voyage database of the British Southern Whale Fishery^69^, which operated from 1775 to 1859, documents the events of around 2,550 whaling or sealing voyages to the south of Britain in over 930 different vessels. The records provided the quantity of whale oil measured in tuns, casks or barrels, but are not linked to a specific geographic position. Therefore, the voyages were selected according to the destination, keeping separate those whose destination was exclusively the southwestern Atlantic Ocean from the records where this area had been included *en route* to other destinations. The catches were organized according to the year the vessel returned from the voyage, as nearly 90% of the voyages analyzed took fewer than two years. The data for French whaling^70^ comes primarily from a digitization of T. Du Pasquier’s books. The criterion for filtering the data was similar to that used for the British Southern Whale Fishery database.
(iii) *The Records of the Boards of Customs (Reference: CUST) of the UK Government obtained from the National Archives* (www.nationalarchives.gov.uk/). The CUST 4 division is available in digital format and contains the ledgers of imports to Britain giving, under the names of the exporting countries, the several articles imported from each along with the quantities and official values. These annual records cover the period between 1809 and 1899. From each of these documents was extracted the information on the quantity of train oil and blubber (tuns) imported from South America to construct a vector of annual whale catches. Unavoidable uncertainties occur with respect to the whaling methods and the precise date—the latter since the year of export would not necessarily be the year the whale was caught. Although how to address this uncertainty was not apparent, this long-term dataset was nevertheless essential for reconstructing the catches that took place during the second half of the nineteenth century, which information is absent in the other sources.
(iv) *Historical catch data for the period 1907–1966, computed from the International Whaling Commission (IWC) catch database*^71^. The data for the illegal Soviet whaling was obtained combing the three available data from Tormosov^21^ for the southwestern Atlantic Ocean with this database.

We converted the reports of whale oil to rough numbers of whales using an average number of 60 barrels of oil (1 tun = 8 barrels; 1 cask = 6.5 barrels) per right whale, following Best^43^. We then filtered all records to select those included in the study area (Fig. 1). This area extends west of 20° W from Salvador de Bahia to the northern Weddell Sea (Mar de Weddell). The selection covered different periods of whaling operations although certain catches were duplicated or triplicated. For example, the records for the French Offshore fleet were partially covered by Du Pasquier^42^, Richards^18^ and the French database from the Whaling History website^70^. An additional source of uncertainty comes from the lack of identification of whale species in the British Southern Whale Fishery database and in the records obtained from the Customs of the UK. Moreover, the term *train oil* probably also included the oil from pinnipeds and humpback whales.

The uncertainties in the catch records were addressed by developing two temporal series of annual catches, a low- and a high–SRW-catch series. We constructed the low series considering the minimum value among overlapped records and excluding the Custom dataset and records with more than one destination. Since only a fraction of the available American offshore whaling logs have been analysed in sufficient detail to provide catch information^68^, all these records were included in the low-catch series. For the high-catch series, we assumed that all the estimated catches from the Custom dataset corresponded to right whales that were killed within the study area.

The catch series were corrected to account for whales struck by whalers but lost during whaling operation. The ‘struck and lost’ rate factor (*SLR*) for the pre-modern period (1771–1850) was set at a value of $SLR_{1}$ ~ N(1.6, 0.04) [1/(1 minus the loss rate)]. For the modern whaling period (1851–1973), the catches were corrected upward through the use of a factor of $SLR_{2}$ ~ N(1.09, 0.04)^72^. No *SLR*s were used for 1648–1770 and 1974-present.

### Estimates of relative abundance

Estimates of relative abundance were calculated using a two-stage approach. First, an aerial-survey protocol as described in Crespo et al.^32^ was performed in 1999, 2000, and 2005–2019. Stated succinctly, the procedure was the following: A monitoring area was defined from the south of Península Valdés to the limit of the main concentration area, totalling a coastal strip 620 km in length. Between 2 and 8 flights were made each year depending on the weather conditions and the financial support. Relative abundance was estimated by counting the total number of whales within the monitoring area and using the methods described in Crespo et al.^32^. Flights were carried out between May 1999 and October 2019. The total number of whales in the area per day was estimated for each year through the generalized-linear-model (GLM) procedure because that method can accurately assess the parameters for the cumulative data, such as the censuses for the SRW^73–75^. As predictor variables, we included Year (categorical), Julian day (continuous), and Julian day^2 (continuous), allowing the models to explore a nonlinear relationship resulting from the seasonal variation. We assumed a negative binomial distribution and log link and estimated parameters using the MASS package in the R Statistical Environment^76^. The second step, employing the parameters of the GLM, involved the use of the estimated daily number of whales for each year to build a cumulative curve that assessed the number of whales that came into the breeding ground each year:

$$ W_{t,y} = e^{{ - a + b_{y} + ct - dt^{2} }} \;1 \le t \le 320,\;1999 \le y \le 2019 $$

where *t* is the Julian day and *y* the year, *W* is the estimated number of whales on Julian day *t* for the year *y*. As the whales come to the area in April, we assumed that $W_{t,y} = 0$ for the range $1 \le t \le 99$. The values for *a*, *b*, *c* and *d* are the parameter estimates from the negative binomial GLM.

Any individual whale will remain in the area a certain number of days that will be shorter than the whole season, and hence the number of whales estimated for the Julian day *t* cannot be directly added to the previous day to build the accumulated curve since the number of transient whales will be seriously overestimated^77^ through a failure to subtract those that have egressed. The time any given whale remains in the area is given by a probability distribution $p\left( k \right)$. We assumed this probability distribution function to be normal $N\left( {\mu = 60; \sigma = 8.66} \right)$; furthermore, the parameters are derived from the only available published information^77^. We also assumed that $p_{k}$ is independent of the day the whale came into the area.

$E_{t,y}$ denotes the number of individuals coming into the area during the Julian day $t$ $\left( {t = 1, 2, 3, \ldots , 320} \right)$ for the year $y$. Thus, if $A_{x}$ is the accumulated number of whales coming into the area from day 1 until day $x$, we can write:

$$ A_{x,y} = \mathop \sum \limits_{t = 1}^{x} E_{t,y} $$

Moreover, we can denote $S_{t,y}$ as indicating to the number of whales that leave the area the Julian day $t$ for the year $y$. The outgoing function $S$ is related to the incoming function by means of the $p_{k}$ distribution in the following way:

$$ S_{t,y} = \mathop \sum \limits_{k = 1}^{t} p_{k} E_{t - k,y} $$

where $t = 1, 2, \ldots , 320$. We assumed that $E_{0,y} = 0$.

Both, the incoming $E$ and the outgoing $S$ functions are related to the estimated number of whales in a particular Julian day $t$ for the year $y$ with the number of estimated whales $W$ the following way:

$$ E_{t,y} - S_{t,y} = W_{t,y} - W_{t - 1,y} = \Delta W_{t,y} $$

hence the difference between the number of whales entering and leaving the area is the same as the difference between the number of estimated whales for any given Julian day *t* and those present the previous day. As we assumed that $W_{0,y} = 0$, so we can be inferred:

$$ E_{t,y} = \Delta W_{t,y} + \mathop \sum \limits_{k = 1}^{t} p_{k} E_{t - k,y} $$

then,

$$ \begin{aligned} A_{x,y} = & \mathop \sum \limits_{t = 1}^{x} E_{t,y} = \mathop \sum \limits_{t = 1}^{x} \Delta W_{t,y} + \mathop \sum \limits_{t = 1}^{x} \mathop \sum \limits_{k = 1}^{t} p_{k} E_{t - k,y} \\ = & W_{x,y} + \mathop \sum \limits_{k = 1}^{x} p_{k} \left( {\mathop \sum \limits_{t = 1}^{x - k} E_{t,y} } \right) = W_{x,y} + \mathop \sum \limits_{k = 1}^{x} p_{k} A_{x - k,y} \\ \end{aligned} $$

The final formulation for the accumulated number of whales is:

$$ A_{x,y} = W_{x,y} + \mathop \sum \limits_{k = 1}^{x} p_{k} A_{x - k,y} $$

If we assume that $A_{0,y} = 0$, within the range of $1 \le x \le 320$ for $x$ and between $1999 \le y \le 2019$ for the year $y$, this function enables an estimation of the accumulated number of whales $A_{x,y}$ (*i.e.*, the total number of animals reaching the survey area during year $y$) through the use of the number of estimated whales for a given Julian day and the preceding one ($A_{x - k,y} , 1 \le k \le x$) (Table S2). Given the small probability of new whales entering the area after mid-November, we set the following restriction $A_{x} = A_{320} , 320 \le x \le 365$.

### Estimates of absolute abundance

Estimated absolute abundance for 2010 of 4,245 (SE: 245) was taken from the IWC^22^.

### Population dynamics modelling

The population dynamics of the SRW from the southwestern Atlantic Ocean was modelled via an age- and sex-aggregated density-dependent model. The model was implemented in a Bayesian state-space framework. This approach is regarded as a powerful tool for modelling time-varying abundance indices because such an implementation simultaneously accounts for both stochastic variability (the state model) and stochastic measurement error (the observation model)^4,78^. The state model accounts for the unobservable stochasticity in the evolution of the animal population over time and with changes in environmental conditions, while the observation model takes into consideration the imperfect detection and sampling variations. With this approach, all the identifiable sources of uncertainty related to the mathematical representation of the biological system were addressed in producing the posterior distribution of the parameters. The key population parameters comprise the carrying capacity (*K*), the maximum rate of increase $\left( {R_{max} } \right)$, the proportion of *K* at which maximum production is achieved (*Pmsy*), and the predictions of population abundance. The annual abundances were treated as unobserved random variables in the Bayesian modelling framework. The model was run for the period 1648–2019 and projected forward to 2030. The input of data for this model included annual-catch records, relative abundance estimates (accumulated numbers of whales), and estimated absolute abundance in 2010. Different modelling scenarios were proposed to test the sensitivity of model outputs to the available data and the assumptions of the model.

The basic population dynamic process was modelled by means of the following discrete formulation:

$$ N_{y + 1} = N_{y} + f\left( {N_{y} } \right) - C_{y} *SLR_{y} $$

where $N_{y}$ is the unknown underlying state variable in year $y$ (in this instance, the unobserved annual abundance for the SRW population exposed to whaling, $y$ = 1648,…,2019), $C_{y}$ the number of individuals removed by commercial whaling in year $y$, $SLR_{y}$ the correction factor for the year (*y*) to account for whales that were struck and lost, and $f\left( \cdot \right)$ a surplus-production function. This function was specified as the following generalized theta-logistic equation^79^:

$$ f\left( {N_{y} } \right) = R_{max} N_{y} \left[ {1 - \left( {\frac{{N_{y} }}{K}} \right)^{z} } \right] $$

where $R_{max}$ is the maximum rate of increase (*i.e*., the intrinsic population growth rate when $N_{y} \sim$ 0), $K$ the carrying capacity, and $z$ a shape parameter that controls the level of nonlinearity in the density-dependence. This parameter was calculated numerically from *Pmsy*.

The two estimated time-series of annual catches, given uncertainty in the number of landed whales, were combined to estimate $C_{y}$ according to Zerbini et al.^80^:

$$ C_{y} = C_{y,min} + \pi *\left( {C_{y,max} - C_{y,min} } \right) $$

where $\pi$, the catch parameter, is a single time-invariant parameter that determines the true landings from $C_{y,min}$ and $C_{y,max}$, which correspond, respectively, to the minimum and maximum total estimated catch in year $y$.

Process error was accounted for in the state process by assuming independent and multiplicative lognormal error structures with the variance parameter $\sigma^{2}$^81^. This model also assumed that the pre-exploitation population was at the environmental carrying capacity before the beginning of whaling operations in 1678 (i.e., $N_{1648 - 1677} = K$). The median population abundance in year $y$ is $\tilde{N}_{y}$ . To avoid estimates of negative abundance and attempting to take the log of a negative number, a lower boundary was placed on $\tilde{N}_{y}$^82^. Therefore, the state process was assumed to follow a centred stochastic transition model as:

$$ \begin{aligned} & \tilde{N}_{1648 - 1677} = K \\ & \tilde{N}_{y + 1} = {\text{max}}\left\{ {N_{c} , N_{y} + f\left( {N_{y} } \right) - C_{y} *SLR_{y} } \right\}\;{\text{for }}y > 1677 \\ & \left. {N_{y} } \right|\tilde{N}_{y} \sim {\text{log}} - {\text{normal}}\left( {{\text{log}}\left[ {\tilde{N}_{y} } \right],\sigma^{2} } \right)\;{\text{for }}y = 1648 - 2030 \\ \end{aligned} $$

where $N_{c}$ is a constraint on the minimum abundance of SRW (described below). The observation process of the stochastic model assumed that the accumulated number of observed right whales $\left( {A_{y} , y = 1999, \ldots , 2019} \right)$ were proportional to the true abundance $\left( {N_{y} } \right)$ through the catchability coefficient $q$ and a parameter that determines the level of density-dependence in catchability $\left( \beta \right)$. Observation error was accounted for by assuming a multivariate lognormal error structure:

$$ \left. {{\varvec{A}}_{{\varvec{y}}} } \right|{\varvec{N}}_{{\varvec{y}}} ,q,\beta ,{\varvec{\varSigma}} \sim {\text{log}} - {\text{normal}}\left( {{\text{log}}\left[ {q{\varvec{N}}_{{\varvec{y}}}^{1 + \beta } } \right],{\varvec{\varSigma}}} \right)\;y = 1999,2000, 2005 - 2019 $$

where ${\varvec{A}}_{{\varvec{y}}}$ and ${\varvec{N}}_{{\varvec{y}}}$ are vectors of the accumulated and estimated number of observed whales, respectively, and ${\varvec{\varSigma}}$ is the variance–covariance matrix of the log accumulated number of observed whales calculated by numerical simulation (Table S3). Catchability was derived by analytically integrating over a $\log \left( q \right)\sim {\text{uniform}}\left( { - \infty ,\infty } \right)$ prior and the multivariate lognormal likelihood, resulting in evaluation of the likelihood with $q$ replaced by the analytical catchability ($\hat{q}$):

$$ \hat{q} = {\text{exp}}\left( {\sum{\varvec{\varSigma}}^{ - 1} {\text{log}}\left( {\frac{{{\varvec{A}}_{{\varvec{y}}} }}{{{\varvec{N}}_{{\varvec{y}}}^{1 + \beta } }}} \right)/\sum{\varvec{\varSigma}}^{ - 1} } \right) $$

to calculate the marginal likelihood. Observation error for absolute abundance in 2010 was accounted for by assuming a univariate lognormal error structure:

$$ \left. {A_{2010} } \right|N_{2010} , \tau^{2} \sim {\text{ log}} - {\text{normal}}\left( {{\text{log}}\left[ {N_{2010} } \right], \tau^{2} } \right) $$

### Minimum population size

We included a constraint (*N*_*c*_) on the minimum abundance of right whales based on the number of maternally inherited mitochondrial DNA (mtDNA) haplotypes. Recent work has sequenced 24 unique mtDNA haplotypes of SRW in Southern Brazil^22^ and we used the IWC recommended 3 × correction for constraining population models to account for males and non-contributing individuals^84^. Therefore, the constraint on minimum abundance was set to *N*_*c*_ = 72 individuals. Sensitivity to the minimum population size was also explored given multiple estimates of the number of haplotypes (described below).

### Parameter estimation

Bayesian estimation was applied to estimate both the abundance trajectory (*N*_1_, …, *N*_*y*_ with *y* = 1648–2030) and the uncertainty in the parameter estimates following a backwards approach^85^ using a sampling-importance-resampling (SIR) algorithm implemented by McAllister et al.^86^. Rather than estimating and assigning a prior to carrying capacity (*K*) directly, the backwards approach assigns a prior to a recent abundance $N_{recent}$ and back-calculates the abundance trajectory. Therefore, the unknown parameters in the model were $R_{max} ,{ }N_{recent} ,P_{msy} ,{ }q,\beta ,{ }SRL_{1} , SRL_{2} , \pi ,\;{\text{and}}\;\sigma^{2}$. Priors for base case and sensitivity models are described below. A total of 20,000 posterior draws were generated for each model. The posterior distribution for catchability was sampled across posterior draws from a multivariate lognormal distribution according to the following density function:

$$ \left. q \right|{\varvec{N}}_{{\varvec{y}}} ,{\varvec{A}}_{{\varvec{y}}} ,\beta , {\varvec{\varSigma}} \sim {\text{ log}} - {\text{normal}}\left( {{\text{log}}\left[ {\frac{{{\varvec{A}}_{{\varvec{y}}} }}{{{\varvec{N}}_{{\varvec{y}}}^{1 + \beta } }}} \right],{\varvec{\varSigma}}} \right) $$

In our Base Case model, vaguely informative prior distributions were used for model parameters, centred at plausible values, and constrained within realistic biological bounds (Table 1). Owing to uncertainty about an appropriate prior mean for $R_{max}$, a uniform prior distribution spanning 0 – 0.11 was chosen. The uniform prior distribution was restricted to a maximum value of 0.11, based on the maximum biologically possible rate of increase^87^. A uniform distribution over the interval 100–10,000 was used to describe the prior for $N_{recent}$ in 2019 (Table 1). The ‘struck and lost’ rate factors for pre-modern and modern eras $\left( {SRL_{1} , SRL_{2} } \right)$ were assumed to be normally distributed (described above). A uniform prior was imposed on $\pi$ spanning 0 to 1. The prior for the process error variance $p\left( {\sigma^{2} } \right)$ was chosen to be a uniform distribution. The lower bound of the uniform distribution was derived by simulating an age-structured population through time with process error in juvenile and adult survival using the model implemented by Punt et al*.*^88^ and calculating the variance in total abundance at equilibrium. To simulate the age-structured population we sampled the following input parameters from^31^: annual calf mortality rate (0.179; S.E. 0.027), annual adult mortality rate (0.026; S.E. 0.003), annual rate of population increase (1.065, S.E. 0.002), age-at-first-pregnancy (7.58, S.E. 0.18). Annual variation was accounted for by adding normally distributed deviates of annual calf (mean 0; S.D. 0.097 ^31^;) and adult survival (mean 0; S.D. 0.19423) estimated from North Atlantic Right Whales^89^. To our knowledge, there are no current estimates of interannual variation in adult mortality rate of SRW. We therefore assumed interannual variation in the log-odds of survival of SRW was similar to that estimated for North Atlantic Right whales. Variance in the population at equilibrium likely represents the lower bound in process error of the total population given that we ignore process error in reproduction. Given a lack of information to inform the upper bound of the uniform distribution, the upper bound was derived by multiplying the lower bound by 10. Model sensitivity to the selected upper bound was explored by fitting models where the upper bound was selected by multiplying the lower bound by 2 and 100 (described below). We assumed no density-dependence in catchability and assumed $\beta = 0$.

**Table 1:** Estimable parameters and prior specifications for Bayesian state-space models. Alternative prior specifications were considered in the sensitivity analyses (Scens 1–14).

Parameter	Base case prior	Alternative prior
$N_{recent}$	$N_{2019} \sim unif\left( {100, 10,000} \right)$	$N_{2004} \sim unif\left( {100, 10,000} \right)$ (Scen6)
Maximum rate of increase	$R_{max} \sim unif\left( {0, 0.11} \right)$	$R_{max} \sim lnorm\left( { - 2.67, 0.5} \right)$ (Scen1)
		$$R_{max} \sim lnorm\left( { - 2.67, 0.3} \right)$$ (Scen2)
		$$R_{max} \sim lnorm\left( { - 2.67, 0.5} \right)t\left( {0.02, 0.11} \right)$$ (Scen3)
Process variance	$\sigma^{2} \sim unif\left( {6.5e^{ - 5} , 6.5e^{ - 4} } \right)$	$\sigma^{2} \sim unif\left( {6.5e^{ - 5} , 6.5e^{ - 3} } \right)$ (Scen4)
		$$\sigma^{2} \sim unif\left( {6.5e^{ - 5} , 1.3e^{ - 4} } \right)$$ (Scen5)
Depletion at maximum sustainable yield	$P_{msy} \sim unif\left( {0.5, 0.8} \right)$	
Struck and lost’ rate factor (period: 1771–1850)	$SRL_{1} \sim norm\left( {1.6,0.04^{2} } \right)$	
Struck and lost’ rate factor (period: 1851–1973)	$SRL_{2} \sim norm\left( {1.09,0.04^{2} } \right)$	
Catch parameter	$\pi \sim unif\left( {0, 1} \right)$	
Catchability	Analytically derived	$\tau^{2} \sim lnorm\left( {{\text{log}}\left( {0.2} \right), 0.5} \right)$ (Scen13)
Density-dependence of catchability	$\beta = 0$	$\beta \sim norm\left( {0, 0.1} \right)$ (Scen14)

A useful diagnostic from a Bayesian numerical integration is the so-called *post-model-pre-data* distribution. These distributions reveal how the priors interact with a model given the catch data but before the model is fitted to an abundance index. This approach enables evaluation of the extent to which fitting the model to abundance index data updates the distributions determined by the interaction of the priors and inputted catch records within the model formulation. The post-model-pre-data distribution was compared to the posterior distribution to indicate the extent of posterior updating on each parameter.

### Sensitivity analysis

Model sensitivity to the prior probability specifications and the input data was evaluated by exploring 14 alternative models to the Base Case scenario (Table S4). `Model assumption` scenarios assessed variation in the model outputs when different prior distributions were specified for $R_{max}$, $\sigma^{2}$, and $N_{recent}$. Scenario Scen 1 evaluated a vaguely informative lognormal prior distribution was used for $R_{max}$ with a mean of 0.069. Distribution parameters were computed from SRW life-history data^22,87^ with a CV of 50%, enabling sufficient flexibility to be able to estimate the probable value of $R_{max}$. In Scen 2, a CV of 30% was used to evaluate the sensitivity of the posterior distribution of $R_{max}$ to a more informative prior distribution. In Scen 3, a truncated lognormal prior on $R_{max} { }$ was used instead of the lognormal prior. The prior was truncated at 2% and 11% (minimum and maximum plausible)^87^. In scenarios Scen 4 and Scen 5, the upper bound of the uniform distribution on ${\upsigma }^{2}$ was moved to 100 times and twice the lower bound, respectively. In Scen 6, a uniform prior from 100 to 10,000 was used for $N_{recent}$ for 2004. The `Catch` scenarios investigated the effects when struck-and-lost rates were excluded (Scen 7), and when only the low (Scen 8) or the high- (Scen 9) catch series was considered. The `Minimum population size` scenarios investigated the impact of an alternative population constraints (*N*_*c*_) based given multiple estimates of the number of mtDNA haplotypes^22^. *N*_*c*_ of zero (Scen 10), 75 (Scen 11), and 111 (Scen 12) were considered to evaluate the lack of population bounds and estimates of 25 and 37 mtDNA haplotypes, respectively. `Index` scenarios evaluated the potential for time-varying and/or density-dependent catchability. In the breeding ground of Peninsula Valdés, because of the population growth, expansion to less dense regions (*i.e*., suboptimal habitats) by some types of groups was recorded^32,39,90^. This change in distribution could mean that catchability of animals in the sampling area is varying over time. Scen 13 evaluated the potential for time-varying catchability by including additional observation error attributable to variation in catchability^91^. An estimated observation error term $\tau^{2}$ was added to the diagonal of ${\varvec{\varSigma}}$. A vaguely uninformative lognormal prior was used for $\tau$ with a mean of 0.2 and CV of 50%. Given habitat use of the survey area may be density-dependent^32^, Scen 14 evaluated the potential for density-dependent catchability by assuming a vaguely informative normal prior with a mean of 0 and SD of 0.1 for $\beta$. The priors used for $\tau$ and $\beta$ in Scen 13 and 14, respectively, were not informed by previous information, but were selected to evaluate model sensitivity to alternative catchability assumptions while also limiting overfitting.

### Model uncertainty

The relative probabilities of the competing models were inferred by calculating Bayes factors assuming all models were equally probable a priori^92^. All models were comparable given they contained the same data. The consistency between the model and the data was checked by the Bayesian posterior-predictive-checking procedures designed to check the ability of the model a posteriori to replicate abundance data similar to those observed. Additionally, we performed multi-model inference using Bayesian model averaging to balance model goodness of fit and model uncertainty, rather than relying on one ‘true’ model^93^. The posterior distributions from the candidate models were sampled relative to the models posterior probability based on the calculated Bayes factors. Scen 4 and Scen 5 and models belonging to the `Catch` scenarios were not included in model averaging because those scenarios were proposed aiming only to assess the sensitivity of model outputs.

## Results

The reconstruction of annual catches suggested that since the mid-seventeenth century the whaling operation had killed between 35,000 to 74,000 SRWs along the east coast of South America, under the scenario of maximal catches (Table S1). The largest number of whales caught occurred from the mid-18th to mid-nineteenth centuries, peaking between 1761 and 1776. The first stage in the whaling activity involved a monopoly of the Portuguese crown, resulting in an expansion of the armação (land whaling station) southwards along the Brazilian coast^59,60^. The Brazilian whalers exploited the breeding stock of SRWs from these coastal whaling stations. During the period 1772–1812, American (40% of the total number of identified whaleships), British (49%), French (8.6%), and Spanish (2.4%) whalers dominated the exploitation of SRWs at the Brazil Bank^18^. The catches of the nineteenth century were led by the British fleet (50% of total catches), followed by the French (28%) and the Portuguese (0.16%). Relatively few whales were taken during the modern whaling period. Until the advent of international protection in 1935, 40% of the whales caught were taken by Norway, 26% by Argentina, 17% by the UK, and 15% by Chilean whalers. Illegal Soviet catches during the period 1951/52–1971/72 peaked in 1961/1962 when 1,335 whales were caught off the Patagonia^21,71^.

**Figure 2:**
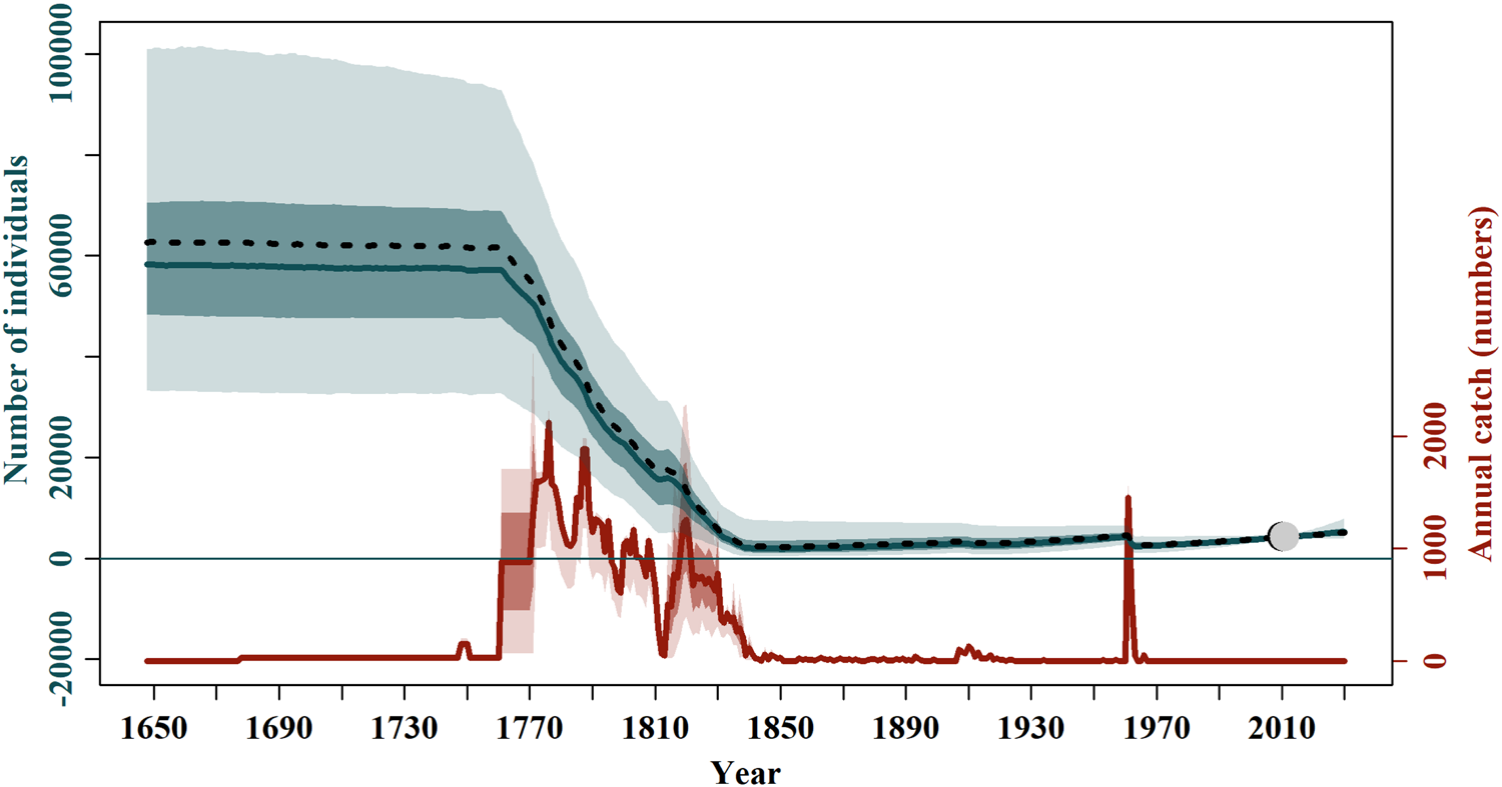
Model averaged population trajectories (blue lines) and time series of estimated catches (red lines) of southern right whale (SRW) *Eubalaena australis.* The solid blue line represents the median estimated model-averaged trajectory of the population abundance (*N*_*y*_), while the shaded areas correspond to the 50% and 95% credible intervals. The dashed line represents the median estimated base case trajectory of the abundance. The solid red line represents the average number of whaling catches as estimated by the catch parameter (π), while the red shaded areas correspond to the 50% and 95% credible intervals. The grey and black dots represent the estimated and observed, respectively, absolute abundance in 2010 (confidence and credible intervals can be found in Fig. S4).

Overall, the Bayesian population dynamic model performed here provided the first estimate of pre-exploitation abundances while predicting reasonable dynamics for the SRW from the southwestern Atlantic Ocean at the regional level. Through the use of the multi-model inference procedure, eleven models were selected to generate the model-averaged trajectory (*i.e.*, the posterior distribution of mean abundances, *N*_*y*_) (Tables S5 and S6, Fig. 2). Table 2 summarises the posterior distributions of the key biological parameters after model averaging. All the parameters fall within biologically plausible constraints. The estimated means were generally greater than the medians, thus indicating a positive skewness. The median model-averaged estimated analytically derived $\hat{q}$ was 0.36, which means that close to 36% of the SRW population visits the waters of the Península Valdés, the main breeding ground, every year.

**Table 2:** Posterior mean, standard deviations and 50% and 95% Bayesian credible intervals (CI) for the key biological parameters estimated by the model-averaged assessment of the southern right whale *Eubalaena australis*.

	Mean	Median	2.5%	25%	75%	97.5%
*R*_*max*_	0.014	0.012	0.001	0.006	0.02	0.034
*K*	60,682	58,212	33,329	48,271	70,580	100,920
*P*_*MSY*_	0.648	0.647	0.506	0.571	0.724	0.793
*σ*	0.0189	0.0197	0.0091	0.0155	0.0229	0.0253
*N*_*Min*_	1,958	1,829	355	969	2,919	3,969
*N*_2021_	4,796	4,742	3,853	4,400	5,143	6,013
*N*_2030_	5,466	5,299	3,886	4,698	6,079	7,931
*P*_*Min*_	0.032	0.03	0.008	0.019	0.042	0.067
*P*_2021_	0.087	0.082	0.042	0.064	0.103	0.16
*P*_2030_	0.1	0.091	0.043	0.069	0.122	0.205

*P*_*Min*_ refers to the minimum estimated abundance relative to *K.*

The model-averaged population trajectory indicated that the pre-exploitation abundance was close to 58,000 individuals (median *K* = 58,212; 95% CI = 33,329–100,920 individuals). After the beginning of whaling operation, the population decreased slightly in abundance followed by a rapid and severe depletion of the SRW population in the early 1770s due, primarily, to the sizeable catches taken along the coast of Brazil (Fig. 2). The abundance dropped to the lowest abundance levels in the 1830s when close to 2,000 individuals (median = 1,829; 95% CI = 355–3,969 individuals) were left along the southwestern Atlantic Ocean. The population remained at low levels during the nineteenth century, and by the early twentieth century was estimated to be around 5% of the pre-exploitation abundance. A brief recovery period was observed after 1920, followed by a second decline in abundance in the 1960s when illegal Soviet whaling operated in the Southern Hemisphere. Since no whaling occurred after 1973, the population increased at a growth rate close to $R_{max}$ (median = 0.012; 95% CI = 0.001–0.034) until the present. The current median population abundance (*N*_2021_) is estimated at 4,742 whales (95% CI = 3,853–6,013), revealing that the SRW population still remains small relative to its pre-exploitation abundance (median depletion *P*_2021_ 8.7%: 95% CI = 4.2–16.1%; Fig. 2). The projected abundance, however, indicates that the population will continue to grow for the next decade. The projected median population abundance in 2030 (*N*_2030_) was estimated at 5,299 whales (95% CI = 3,886–7,931).

**Figure 3:**
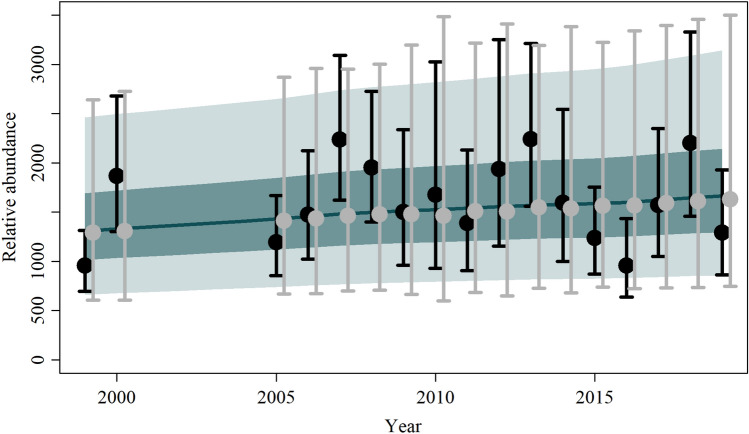
Trend of the observed (black dots) and estimated (grey dots) accumulated numbers of the southern right whale *Eubalaena australis* and associated 95% confidence interval (black bars) and 95% posterior predictive intervals (grey bars). The solid blue line represents the median estimated model-averaged trajectory of the population abundance (*N*_*y*_) multiplied by posterior catchability (q), while the shaded areas correspond to the 50% and 95% credible intervals.

The data were compared with the median and the 95% credible intervals of the corresponding posterior predictive distributions, with all the observed abundance points falling within the 25th and 75th percentiles of the predictions (*A*_*y*_) provided by the model (Fig. 3 and Fig. S4.1–S15).

**Figure 4:**
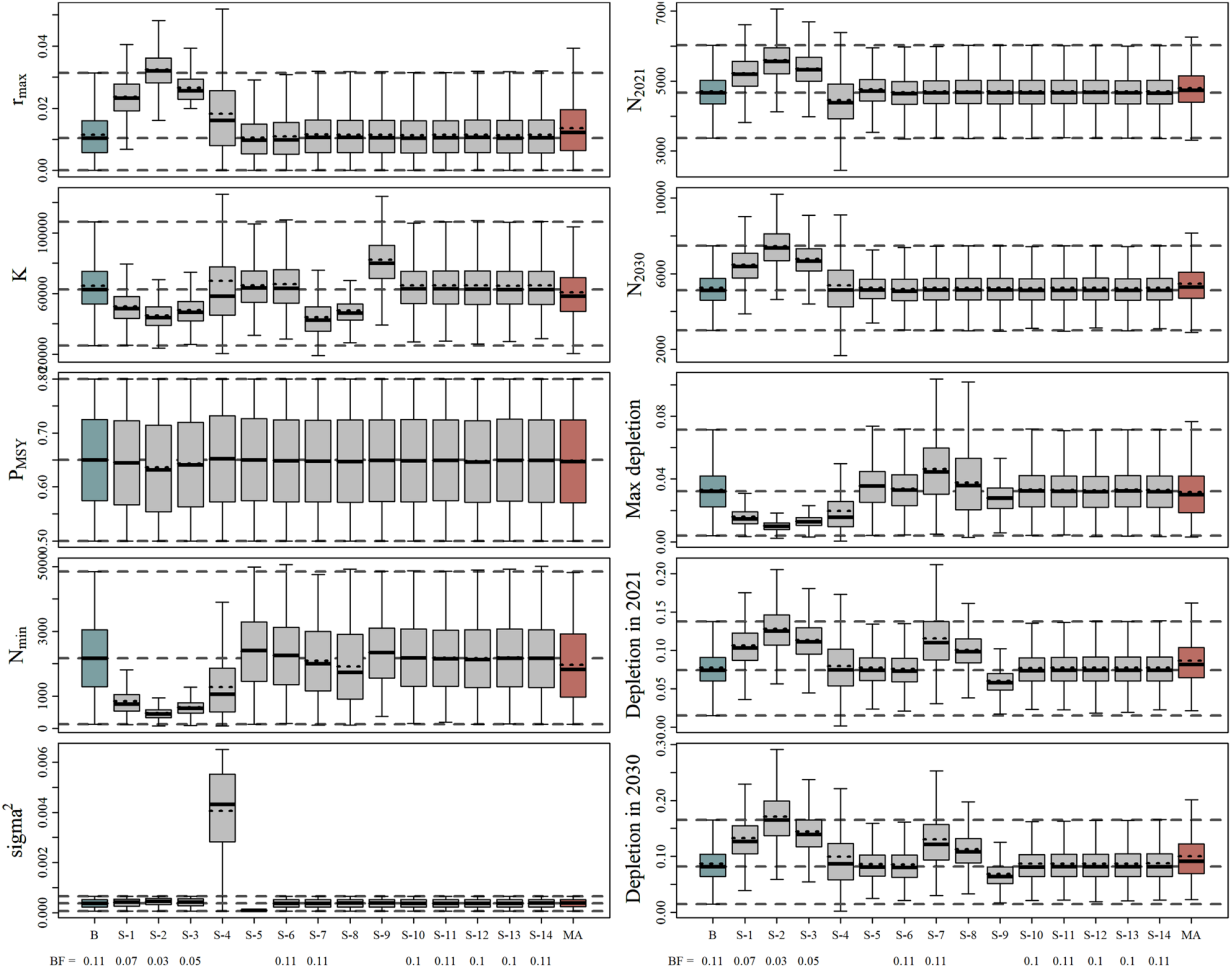
Posterior probability distribution of the key biological parameters for the Base Case (blue), sensitivity scenarios (grey), and model-averaged (red) assessment of southern right whale (SRW) *Eubalaena australis*. The mean (dotted line in the boxes) and median (solid black lines in boxes) estimates, first and third quartiles (boxes), and the 95% CIs (whiskers) are presented. Bayes factors are presented for each scenario at the bottom.

**Figure 5:**
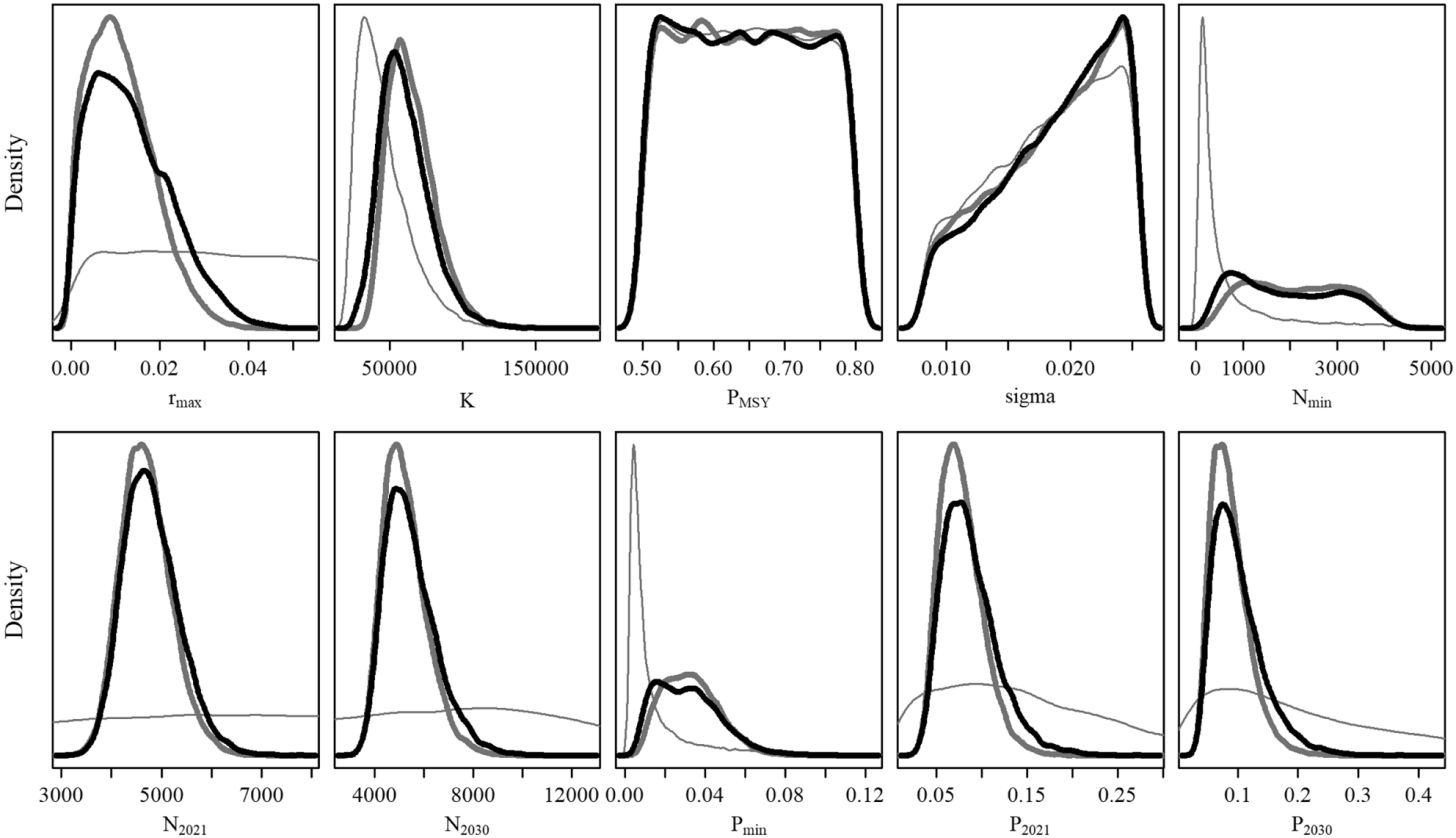
Posterior probability distributions of the key biological parameters for the Base Case (thick grey line) and model-averaged (thick black line) assessment of southern right whale (SRW) *Eubalaena australis*. Post-model pre-data probability distributions of the key biological parameters for the Base Case are presented in the thin grey line.

Figure 4 illustrates the posterior distribution of the model parameters selected for the Base Case and the fourteen sensitivity scenarios. All the models implemented converged adequately given the number of unique samples derived from the SIR algorithm (> 10,000). Despite the relatively wide posterior distributions, most key parameter distributions differed from the prior and post-model-pre-data distributions, suggesting that the information content in the likelihood was informative for parameter estimation (Fig. 5 and Fig. S2.1–S15).

In general terms, the posterior probability distributions of the proposed sensitivity scenarios were consistent with the Base Case scenario (Fig. 4). However, when a lognormal prior on population growth was used (Scen 1–3), the posterior distribution was updated to higher values (Fig. S2.1–4) and historical abundance was decreased (Fig. S3.1–4). The posteriors for the process error variance (Scen 4–5) were sensitive to their prior distribution, and had some effect on the other estimated parameters. For example, increasing the upper bound on the process error variance (Scen 4) led to increased uncertainty in $R_{max}$ and $K$ and a lower minimum population size. Posterior medians for $R_{max} , K$ and $q$ were slightly lower when the struck-and-lost rate factors were not included in the analyses (Scen 7) or when the catches were modelled with the low- SRW catch series (Scen 8). Overall, apart from scaling the population size, the parameter estimates were relatively insensitive to the `Catch` scenarios. Similarly, changing the prior for $N_{recent}$ (Scen 10), altering the number of mtDNA haplotypes (Scens 11–12), or accounting for density dependence/time-varying catchability (Scens 13–14) had little impact on the results. However, for Scen 13, the additional observation error to account for time-varying catchability $\left( {\tau^{2} } \right)$ was poorly estimated (Fig. S2). The posterior 95% CI for $\beta$ in Scen 14 overlapped with 0 (Table S5), indicating limited evidence in support of density dependent catchability.

## Discussion

The SRW was one of the baleen whale species that has experienced long periods of exploitation. Here, we integrated information from multiple sources for a better understanding of the SRW population dynamic from the southwestern Atlantic Ocean over a 370-year time frame. The modelling approach was based on a Bayesian state-space surplus production model that, for the first time, enabled to estimate the population trajectory and its historical carrying capacity. An in-depth reconstruction of whaling catches was essential to generate credible population dynamics. Our estimates of the total SRW removals from the study area since the seventeenth century are higher than previous estimates, which were limited to shorter time periods or smaller areas than considered here. Richards^18^ reported a total removal of 30,000 whales during the period 1772–1813, this value is close to our low catch-series. If this catch series is corrected by the ‘struck and lost’ rate factors, at least 50,000 SRW were removed from the southwestern Atlantic Ocean since the beginning of whaling activities.

### Uncertainties in the data and the structure of the model

Stock assessments of cetacean species are subject to different sources of uncertainty that impact the estimation of population trajectories. The Bayesian state-space framework implemented here enabled that uncertainty in the estimates of parameters, catch data, biological stochasticity, and measurement error to be adequately incorporated^78^. The selection of plausible prior distributions for parameters combined with the multi-model inference provided reliable posterior distributions for the parameters. Although the slightly wide posterior credible intervals suggested some degree of uncertainty in the parameters, no estimation problems were diagnosed upon subjection to the analytical criteria. The posterior distribution for most of the estimated parameters were smooth and unimodal (Fig. S2). Alternative possibilities such as a larger sample size or sampling replicates could substantially improve the capacity of the state-space model for monitoring biological populations^94,95^, but for many observational studies such as those performed here, to have replicates of estimates is often impossible. Similarly, in a Bayesian framework, specifying informative priors typically stabilizes model fitting and reduces the uncertainty regarding estimated quantities, but specification of the latter should rely on a solid foundation such as meta-analyses or independent data.

The model-structure uncertainty—within the context of analyses of cetacean populations—is related mainly to uncertainties concerning the stock structure^96^. In the example of SRW, the biological and historical evidence do not currently suggest multiple separate populations calving in the southwestern Atlantic Ocean. Recent evidence from genetic data indicates a connectivity between Brazil and Argentina. Samples from the South Georgia (Georgias del Sur) feeding ground were associated with wintering grounds in the South Atlantic, rather than the Indo-Pacific and were closer to the Argentinian and Brazilian wintering grounds than to the South-African^50^. The authors also suggested that the Chile-Peru population could have historically been a “stepping stone” between the south Atlantic and Indo-Pacific, rather than more closely linked to the southwest Atlantic wintering grounds as they had initially hypothesized. Analyses of SRWs satellite-tracked from the north of Península Valdés indicated areas of potentially major foraging on the outer continental shelf off southern South America, the South Atlantic Basin, the Eastern Scotia Sea (Mar de Escocia), and the northern Weddell Sea (Mar de Weddell)^45^. All these findings were considered during the filtering of whaling records from the multiple historical datasets studied for the generation of the time-series of catches impacting on population dynamics.

Whaling catches have been identified as another source of uncertainty that usually has great impact on estimates of the current abundance of cetacean populations^97^. Zerbini et al.^80^ assessed the recovery of southwestern Atlantic humpback whales (*Megaptera novaeangliae*) and found that scenarios where pre-modern whaling catches and struck-and-lost rates were not included resulted in a lower estimate of pre-exploitation abundance and higher estimates of the status parameters, thus underscoring the need to incorporate all available catches and loss rates in cetacean assessments. Although certain caveats associated with catch series estimates still remain (*e.g*., the barrel-to-whale conversion factor, local whaling stations for which no catch records exist, the patchiness of import records), the large amount of data compiled here about the whaling operation in the South Atlantic and information about the ‘struck and lost’ rate^47^ enable us to minimise the sources of uncertainty in the whaling catches.

The uncertainty or gaps in the whaling records may also lead to an imprecise estimate of the pre-exploitation baseline because analyses for cetacean stocks conventionally started in the first year for which catches were recorded with the assumption being made that the stock was at carrying capacity at that time. The choice of baseline year is not a trivial task and may lead to unrealistically low estimates of depletion levels, having considerable management implications for the rebuilding and conservation of these populations^98–100^. In general, the estimates of carrying capacity from analyses in which the projections start fairly recently (*e.g.*, when the model projections start after the stock has been subject to unknown previous catches) are imprecise^96^. Recently, Collins et al.^101^ demonstrated that for eight species of Canadian mammals the use of 1850 rather 1970 as the baseline year resulted in a shift of four species from an increase to a decrease in population since 1970. For the SRW, in 2001, the IWC performed a global assessment to obtain an estimate of the initial population size^19^ setting the pre-exploitation baseline in 1770. This analysis estimated recovery levels at approximately 20–25% in 2009 for the Southern Hemisphere populations. In our study, the population was assumed to be at equilibrium (carrying capacity) in 1678, when the first estimates of annual catches are available^60^. Before this year, coastal whaling activity was restricted to northern Brazil and its catches were assumed to be negligible within the context of the history of whaling. Overall, through the use of long-term corrected whaling records and an accounting for certain key uncertainties (*e.g*. through a state-space framework, sensitivity analyses and model averaging) by a Bayesian modelling approach, this study presents a reasonable assessment of the population dynamics of the SRW in the southwestern Atlantic Ocean.

### Population modelling

The estimated model-averaged trajectory suggested that the pre-exploitation SRW population abundance was between 48,000–70,000 (50% CI) individuals. The most plausible values agreed with the results obtained from other populations and basins. Jackson et al.^102^ estimated a pre-exploitation abundance of the New Zealand SRW population at between 28,000–47,000 individuals. For the SRWs off South Africa, the initial population size was roughly estimated at 20,000 individuals^103^, based on a cumulative catch estimate of 12,000 whales from 1785 to 1805. Within the circumpolar area, the most recent analysis estimated a total of 95,000–102,000 including both males and females^14^. Those authors also estimated a pre-exploitation abundance totalling 70,000 individuals for the Atlantic and Indian Oceans; which figure is consistent with the estimates available for South Africa and the southwestern Atlantic Ocean, though information is lacking with respect to other management units. Although the current state of knowledge about the interplay of biotic and abiotic factors of the Southern Ocean is incomplete, a credible estimate of the baseline state of the system has enabled us to have a more comprehensive understanding of the true impact of anthropic activities.

Our model indicated that the population had collapsed by 1830. de Morais et al.^67^ had reconstructed the pre-modern catch data in the tropical southwestern Atlantic Ocean by establishing a relationship between whaling stations’ history and the distribution of the species hunted along the coast. That study suggested that this population collapsed within the same decade based on increasingly rare reports of sightings for the nineteenth century and the closing of the last armação in the breeding grounds off southern Brazil.

The current observed abundances were accurately estimated by the SRW model (Fig. 3), suggesting a total population abundance in 2021 of close to 5,000 individuals throughout the study area. This figure implies a recovery rate lower than 10% from the pre-exploitation abundance. Similar rates were estimated for the New Zealand region (*i.e*., 7–12%)^102^, and circumpolar distribution. Tulloch et al.^14^ estimated that the SRWs worldwide remain at less than 11% of their estimated carrying capacity. The South-African population manifested a total abundance of 6,116 animals in 2017, suggesting a recovery of over 25%, although mention was made that this rate needed a reconsideration^24,104^. The SRW recovery level is lower than the humpback whale, which species exhibits an estimated current population of 93% of its pre-exploitation size in the southwestern Atlantic Ocean^80^. This recovery can be explained in part by the high growth rate estimated for this population (median $R_{max}$ = 0.087).

The slow recovery rate of the SRW may be the consequence of multiple causes in a process that is undoubtedly dynamic. Relatively limited removals in the late nineteenth and twentieth centuries from a population at an extremely low abundance constituted a major condition contributing to the failure of SRW recovery in the southwestern Atlantic Ocean for more than 100 years. Recent SRW-calf die-offs at Península Valdés has been identified as a potential threat to future recovery^105^. The fidelity to migratory destinations has been inferred to explain the spatially variable recovery of the SRW^27,106^ and humpback whale^107,108^. The apparent loss of cultural memory when whales are extirpated that display fidelity to a migratory destination likewise seems to be contributing to the slow recovery of the SRW^28,103^. This influence could also be exacerbated if individuals show fidelity to suboptimal feeding grounds. In the southwestern Atlantic Ocean, González Carman et al.^109^, using Ensemble Distribution Models, found evidence that the Subtropical Frontal Zone and the Polar Front systems stand as prominent potential feeding grounds for SRWs from late spring to early fall within the circumpolar region. Those authors hypothesized that the cultural memory of feeding in the relatively stable and predictable high productive waters of the Subtropical Frontal Zone might have been lost because of whaling. The location of the whaling operations supports this hypothesis since most of the whaling records involving SRWs have occurred at mid-latitudes. Moreover, reproductive success has been linked to climate conditions through a negative effect on foraging-ground quality^110,111^; and accordingly, in view of the current global-warming projections, the level of recovery of the SRW population from the southwestern Atlantic Ocean would be compromised in overcoming years of low recruitment through changes in prey distribution and abundance driven by climate^111^.

Nevertheless, the differences in movement patterns reported from satellite tracking^45^ along with the diversity of food sources revealed from isotopic signals^47,48^ have suggested a degree of plasticity in the migratory fidelity of SRWs in the southwestern Atlantic Ocean. This notion is also supported by evidence of recolonization of previously inhabited migratory destinations^33,39–41^. Sueyro et al.^90^, assessing the change in the distribution of SRWs in the breeding grounds of Península Valdés, proposed that a threshold in the whale’s density within breeding areas triggered a density-dependent response, with the mother-calf pairs remaining in the area and the other groups being displaced to new regions. Currently, the underlying process of the observed recovery of the SRW population from the southwestern Atlantic Ocean appears to involve a slower increase within the traditional feeding ground^32^ accompanied by a higher probability of recolonizing ancient habitats^39,90^. These findings, together with model estimates for the entire study area, lead us to propose that the SRW population will continue growing for many decades, though density-dependent effects on population parameters can be expected.

### Limitations and future directions

Bayesian surplus production models are useful when the available data comprise only aggregate catches and population abundance time series^112^. Although modern catch series offer information on sex composition^71^, most pre-modern whaling data on the SRW are only available on the species level or have been reconstructed by converting barrels of landed and exported oil into whale numbers (*e.g*., the data from the Records of the Boards of Customs of UK). Currently, the lack of age- or size-structured data for SRW catches from Brazil Bank limits the development of models that enable an assessment of selective catching (*e.g*. mothers with dependent calves) to the population trajectory. Nevertheless, production models are widely used for the stock assessment of cetaceans (*e.g.*, 80,102), and experience has often indicated little justification for the inclusion of sex-structure in such analyses^96^. A notable exception occurs when catches have been female-biased, in which instance the decline in the population could be underestimated.

The density-dependent logistic model used here assumes that $K$ and $R_{max}$ are constant over time. This assumption agrees with the current modelling approach used by the IWC. The time-invariant carrying capacity would limit the ability of our model to project the population trajectory of the SRW forward over the long-term if both food availability and space to reproduce change over time, thus reducing the environmental carrying capacity. Past changes in available calving and foraging habitats are less likely to have affected the estimated trajectory since the population remained at an extremely low level of abundance over the past 200 years. A recent study coupled a predator–prey model to a global climate change model to project the SRW population in the Atlantic and Pacific basins forward to 2100. This study suggested that, despite the initial recovery from historical whaling, the circumpolar populations will show substantial reductions in total numbers by the end of this century under the climate change scenery^113^.

Even though the prior for the maximum rate of population increase was set at values close to the highest rates of annual increase (*R*) reported for the population^22,30,31,87^ (Scen 1), our model updated the distribution of the parameter, favouring values below 6%. The trend of combined direct-surveys data and catch-series data does not support a higher maximum rate of increase, as suggested by the stage-structured model based on photo-id data^31^. Similar results were observed for the New Zealand SRW population, where the density-dependent model used favoured population growth rates below 6% since higher rates were associated with a low bottleneck in abundance^102^.

Changes in the catchability in the surveyed area could explain the low maximum rate of increase estimated. As mentioned previously, the observed redistribution of the whales in the Península Valdés area^32,39,90^ suggests that the area is getting close to its carrying capacity in the survey area as optimal habitat while solitary individuals and breeding groups are forced to use deeper waters or to move out of the region, in particular to Golfo San Matías^39,90^. Although `Index` scenarios exploring time-varying and/or density-dependent catchability were not able to estimate high rates of increase, models that assume a negative trend in catchability might favour higher values. Unfortunately, information about temporal variation in the proportion of the population inside the survey area is not available to better estimate a trend in catchability. If abundance in the traditional breeding grounds remains stable over time and the population continues to increase across its distribution, future studies should prioritize the generation of information to estimate the trend in catchability.

On the other hand, different age- and sex-structured population dynamics models have been explored to identify the existence and cause of delayed density-dependent feedbacks in seven species of baleen whales (suborder Mysticeti)^114^. A model with a growth rate that is not fixed and is density-regulated by intraspecific natural selection was the most plausible for explaining the observed time lag. The selection-delayed model estimated higher rates of current growth for all species and a higher initial abundance for the SRW. These conclusions have implications on the conservation status of the species and should be explored in future assessments of the SRW population from the southwestern Atlantic Ocean. At the same time, the present analysis did not account for other possible low anthropic impacts—*e.g*., habitat degradation, ship strikes, entanglement, kelp seagull harassment, impact of man-made debris—that would probably lead to an overestimation of the current conservation status of the population. Continued annual monitoring of the population will be essential to assess progress toward recovery and will, therefore, allow for validation (or not) of the results presented here on a scale that fitted to correspond to the analysis of the species past disruption.

### Conclusion

The SRW is recovering from severe depletion, but is still far from its historical abundance. In the present work, we assessed for the first time the population dynamics of the SRW population from the southwestern Atlantic Ocean employing a backward reconstruction of the population trajectory. The density-regulated model implemented produced plausible estimates of the SRW population trajectory and life-history parameters. This satisfactory approximation of the trajectory was made possible by integrating our long-term whale abundance database with the reconstructed series of catch data. Our results provide insights into the severity of the whaling operations that occurred in the South Seas and the way in which the population responded at low densities, thus contributing to an understanding of the observed differences in population trends over the worldwide distribution of the species. We also contributed to a filling-in of the gaps in the exploitation history, mainly within the pre-modern period. Overall, the results constitute a baseline for future studies aimed at accounting for alternative modelling structures and data (*e.g*., mark-recapture data, catchability trend), and additional anthropically caused cetacean mortalities.

## Supplementary Information

Supplementary Information.

## Data Availability

All the data generated or analysed during this study are included in this published article (and its Supplementary Information files). The Model runs, data and R code used in all population modelling can be found on GitHub (https://github.com/SW-atlantic-right-whale-2021-assessment/RightwhaleRuns). An R package developed to implement the SIR model is also available on GitHub (R Package: https://github.com/SW-atlantic-right-whale-2021-assessment/StateSpaceSIR).
