# Supplementary material for: Historical reconstruction of the population dynamics of southern right whales in the southwestern Atlantic Ocean
Source: Sci Rep. 2022 Feb 28;12:3324. doi: 10.1038/s41598-022-07370-6 (PMC8885757; doi:10.1038/s41598-022-07370-6)
Supplement: Supplementary file 1 — Supplementary Information. [file 41598_2022_7370_MOESM1_ESM.docx]

**Historical reconstruction of the population dynamics of southern right whales in the southwestern Atlantic Ocean**

Romero, M.A., M.A. Coscarella, G.D. Adams, J.C. Pedraza, R. González and E.A. Crespo

**Supplementary material**

Table S1. Observed catch series (number of individuals) used in this study. $C_{y}$: annual catch values.

| Year | $C_{y,min}$ | $C_{y,max}$ | Year | $C_{y,min}$ | $C_{y,max}$ |
| --- | --- | --- | --- | --- | --- |
| 1678 | 20 | 30 | 1849 | 2 | 28 |
| 1679 | 20 | 30 | 1850 | 6 | 34 |
| 1680 | 20 | 30 | 1851 | 1 | 1 |
| 1681 | 20 | 30 | 1852 | 1 | 1 |
| 1682 | 20 | 30 | 1853 | 0 | 2 |
| 1683 | 20 | 30 | 1854 | 0 | 0 |
| 1684 | 20 | 30 | 1855 | 0 | 2 |
| 1685 | 20 | 30 | 1856 | 1 | 44 |
| 1686 | 20 | 30 | 1857 | 6 | 8 |
| 1687 | 20 | 30 | 1858 | 7 | 19 |
| 1688 | 20 | 30 | 1859 | 5 | 6 |
| 1689 | 20 | 30 | 1860 | 8 | 14 |
| 1690 | 20 | 30 | 1861 | 0 | 10 |
| 1691 | 20 | 30 | 1862 | 5 | 16 |
| 1692 | 20 | 30 | 1863 | 15 | 34 |
| 1693 | 20 | 30 | 1864 | 7 | 22 |
| 1694 | 20 | 30 | 1865 | 0 | 23 |
| 1695 | 20 | 30 | 1866 | 5 | 17 |
| 1696 | 20 | 30 | 1867 | 0 | 3 |
| 1697 | 20 | 30 | 1868 | 0 | 24 |
| 1698 | 20 | 30 | 1869 | 0 | 45 |
| 1699 | 20 | 30 | 1870 | 0 | 19 |
| 1700 | 20 | 30 | 1871 | 0 | 16 |
| 1701 | 20 | 30 | 1872 | 0 | 25 |
| 1702 | 20 | 30 | 1873 | 0 | 7 |
| 1703 | 20 | 30 | 1874 | 0 | 27 |
| 1704 | 20 | 30 | 1875 | 0 | 10 |
| 1705 | 20 | 30 | 1876 | 0 | 5 |
| 1706 | 20 | 30 | 1877 | 0 | 4 |
| 1707 | 20 | 30 | 1878 | 2 | 17 |
| 1708 | 20 | 30 | 1879 | 5 | 21 |
| 1709 | 20 | 30 | 1880 | 0 | 27 |
| 1710 | 20 | 30 | 1881 | 0 | 12 |
| 1711 | 20 | 30 | 1882 | 0 | 21 |
| 1712 | 20 | 30 | 1883 | 0 | 36 |
| 1713 | 20 | 30 | 1884 | 0 | 15 |
| 1714 | 20 | 30 | 1885 | 0 | 7 |
| 1715 | 20 | 30 | 1886 | 0 | 12 |
| 1716 | 20 | 30 | 1887 | 1 | 28 |
| 1717 | 20 | 30 | 1888 | 4 | 23 |
| 1718 | 20 | 30 | 1889 | 3 | 40 |
| 1719 | 20 | 30 | 1890 | 0 | 13 |
| 1720 | 20 | 30 | 1891 | 0 | 18 |
| 1721 | 20 | 30 | 1892 | 0 | 2 |
| 1722 | 20 | 30 | 1893 | 0 | 73 |
| 1723 | 20 | 30 | 1894 | 0 | 41 |
| 1724 | 20 | 30 | 1895 | 0 | 8 |
| 1725 | 20 | 30 | 1896 | 0 | 0 |
| 1726 | 20 | 30 | 1897 | 0 | 2 |
| 1727 | 20 | 30 | 1898 | 0 | 0 |
| 1728 | 20 | 30 | 1899 | 0 | 32 |
| 1729 | 20 | 30 | 1900 | 0 | 0 |
| 1730 | 20 | 30 | 1901 | 0 | 0 |
| 1731 | 20 | 30 | 1902 | 0 | 0 |
| 1732 | 20 | 30 | 1903 | 0 | 0 |
| 1733 | 20 | 30 | 1904 | 0 | 0 |
| 1734 | 20 | 30 | 1905 | 0 | 0 |
| 1735 | 20 | 30 | 1906 | 0 | 0 |
| 1736 | 20 | 30 | 1907 | 93 | 93 |
| 1737 | 20 | 30 | 1908 | 65 | 65 |
| 1738 | 20 | 30 | 1909 | 56 | 56 |
| 1739 | 20 | 30 | 1910 | 117 | 117 |
| 1740 | 20 | 30 | 1911 | 95 | 95 |
| 1741 | 20 | 30 | 1912 | 26 | 26 |
| 1742 | 20 | 30 | 1913 | 67 | 67 |
| 1743 | 20 | 30 | 1914 | 21 | 21 |
| 1744 | 20 | 30 | 1915 | 19 | 19 |
| 1745 | 20 | 30 | 1916 | 15 | 15 |
| 1746 | 20 | 30 | 1917 | 54 | 54 |
| 1747 | 20 | 30 | 1918 | 14 | 14 |
| 1748 | 104 | 196 | 1919 | 15 | 15 |
| 1749 | 104 | 196 | 1920 | 13 | 13 |
| 1750 | 104 | 196 | 1921 | 10 | 10 |
| 1751 | 20 | 30 | 1922 | 1 | 1 |
| 1752 | 20 | 30 | 1923 | 12 | 12 |
| 1753 | 20 | 30 | 1924 | 12 | 12 |
| 1754 | 20 | 30 | 1925 | 1 | 1 |
| 1755 | 20 | 30 | 1926 | 1 | 1 |
| 1756 | 20 | 30 | 1927 | 3 | 3 |
| 1757 | 20 | 30 | 1928 | 3 | 3 |
| 1758 | 20 | 30 | 1929 | 1 | 1 |
| 1759 | 20 | 30 | 1930 | 1 | 1 |
| 1760 | 20 | 30 | 1931 | 2 | 2 |
| 1761 | 20 | 1750 | 1932 | 1 | 1 |
| 1762 | 20 | 1750 | 1933 | 1 | 1 |
| 1763 | 20 | 1750 | 1934 | 9 | 9 |
| 1764 | 20 | 1750 | 1935 | 0 | 0 |
| 1765 | 20 | 1750 | 1936 | 0 | 0 |
| 1766 | 20 | 1750 | 1937 | 0 | 0 |
| 1767 | 20 | 1750 | 1938 | 0 | 0 |
| 1768 | 20 | 1750 | 1939 | 1 | 1 |
| 1769 | 20 | 1750 | 1940 | 1 | 1 |
| 1770 | 20 | 1750 | 1941 | 0 | 0 |
| 1771 | 0 | 1750 | 1942 | 0 | 0 |
| 1772 | 1002 | 1002 | 1943 | 0 | 0 |
| 1773 | 1003 | 1003 | 1944 | 0 | 0 |
| 1774 | 1006 | 1006 | 1945 | 0 | 0 |
| 1775 | 1016 | 1016 | 1946 | 1 | 1 |
| 1776 | 1326 | 1326 | 1947 | 0 | 0 |
| 1777 | 994 | 994 | 1948 | 0 | 0 |
| 1778 | 970 | 970 | 1949 | 0 | 0 |
| 1779 | 880 | 880 | 1950 | 0 | 0 |
| 1780 | 772 | 772 | 1951 | 0 | 0 |
| 1781 | 700 | 700 | 1952 | 0 | 0 |
| 1782 | 645 | 645 | 1953 | 5 | 5 |
| 1783 | 635 | 635 | 1954 | 0 | 0 |
| 1784 | 670 | 670 | 1955 | 1 | 1 |
| 1785 | 907 | 907 | 1956 | 0 | 0 |
| 1786 | 857 | 857 | 1957 | 10 | 10 |
| 1787 | 1175 | 1175 | 1958 | 0 | 0 |
| 1788 | 1175 | 1175 | 1959 | 0 | 0 |
| 1789 | 854 | 854 | 1960 | 1 | 1 |
| 1790 | 720 | 720 | 1961 | 1335 | 1335 |
| 1791 | 791 | 791 | 1962 | 509 | 509 |
| 1792 | 780 | 780 | 1963 | 37 | 37 |
| 1793 | 760 | 760 | 1964 | 0 | 0 |
| 1794 | 610 | 610 | 1965 | 0 | 0 |
| 1795 | 780 | 780 | 1966 | 47 | 47 |
| 1796 | 548 | 548 | 1967 | 0 | 0 |
| 1797 | 518 | 518 | 1968 | 0 | 0 |
| 1798 | 396 | 396 | 1969 | 0 | 0 |
| 1799 | 376 | 376 | 1970 | 0 | 0 |
| 1800 | 650 | 650 | 1971 | 0 | 0 |
| 1801 | 673 | 673 | 1972 | 0 | 0 |
| 1802 | 601 | 601 | 1973 | 1 | 1 |
| 1803 | 727 | 727 | 1974 | 0 | 0 |
| 1804 | 581 | 581 | 1975 | 0 | 0 |
| 1805 | 577 | 577 | 1976 | 0 | 0 |
| 1806 | 553 | 553 | 1977 | 0 | 0 |
| 1807 | 474 | 474 | 1978 | 0 | 0 |
| 1808 | 667 | 667 | 1979 | 0 | 0 |
| 1809 | 543 | 543 | 1980 | 0 | 0 |
| 1810 | 400 | 400 | 1981 | 0 | 0 |
| 1811 | 184 | 184 | 1982 | 0 | 0 |
| 1812 | 40 | 40 | 1983 | 0 | 0 |
| 1813 | 34 | 34 | 1984 | 0 | 0 |
| 1814 | 0 | 673 | 1985 | 0 | 0 |
| 1815 | 0 | 647 | 1986 | 0 | 0 |
| 1816 | 0 | 1027 | 1987 | 0 | 0 |
| 1817 | 71 | 918 | 1988 | 0 | 0 |
| 1818 | 113 | 1154 | 1989 | 0 | 0 |
| 1819 | 201 | 1436 | 1990 | 0 | 0 |
| 1820 | 233 | 1454 | 1991 | 0 | 0 |
| 1821 | 171 | 1216 | 1992 | 0 | 0 |
| 1822 | 133 | 774 | 1993 | 0 | 0 |
| 1823 | 148 | 864 | 1994 | 0 | 0 |
| 1824 | 136 | 835 | 1995 | 0 | 0 |
| 1825 | 244 | 762 | 1996 | 0 | 0 |
| 1826 | 163 | 754 | 1997 | 0 | 0 |
| 1827 | 173 | 805 | 1998 | 0 | 0 |
| 1828 | 122 | 788 | 1999 | 0 | 0 |
| 1829 | 172 | 663 | 2000 | 0 | 0 |
| 1830 | 187 | 854 | 2001 | 0 | 0 |
| 1831 | 195 | 274 | 2002 | 0 | 0 |
| 1832 | 203 | 233 | 2003 | 0 | 0 |
| 1833 | 229 | 309 | 2004 | 0 | 0 |
| 1834 | 199 | 243 | 2005 | 0 | 0 |
| 1835 | 78 | 443 | 2006 | 0 | 0 |
| 1836 | 107 | 232 | 2007 | 0 | 0 |
| 1837 | 13 | 378 | 2008 | 0 | 0 |
| 1838 | 21 | 258 | 2009 | 0 | 0 |
| 1839 | 1 | 60 | 2010 | 0 | 0 |
| 1840 | 2 | 146 | 2011 | 0 | 0 |
| 1841 | 2 | 83 | 2012 | 0 | 0 |
| 1842 | 0 | 29 | 2013 | 0 | 0 |
| 1843 | 2 | 21 | 2014 | 0 | 0 |
| 1844 | 1 | 1 | 2015 | 0 | 0 |
| 1845 | 4 | 79 | 2016 | 0 | 0 |
| 1846 | 3 | 6 | 2017 | 0 | 0 |
| 1847 | 0 | 67 | 2018 | 0 | 0 |
| 1848 | 5 | 45 | 2019 | 0 | 0 |

Table S2. Observed total number of whales from the aerial-survey across the monitoring area south of Península Valdés to the limit of the main concentration area, totalling a coastal strip 620 km in length.

| Flight | Year | Month | Observed whales | Julian day |
| --- | --- | --- | --- | --- |
| 1 | 1999 | 5 | 5 | 139 |
| 2 | 1999 | 7 | 117 | 183 |
| 3 | 1999 | 8 | 460 | 229 |
| 4 | 1999 | 9 | 549 | 272 |
| 5 | 1999 | 11 | 172 | 314 |
| 6 | 1999 | 12 | 10 | 349 |
| 9 | 2000 | 5 | 43 | 149 |
| 10 | 2000 | 7 | 323 | 194 |
| 11 | 2000 | 9 | 558 | 271 |
| 12 | 2000 | 11 | 296 | 319 |
| 19 | 2005 | 6 | 84 | 159 |
| 20 | 2005 | 7 | 591 | 200 |
| 21 | 2005 | 9 | 733 | 251 |
| 22 | 2005 | 10 | 217 | 298 |
| 23 | 2005 | 12 | 3 | 351 |
| 25 | 2006 | 3 | 1 | 87 |
| 26 | 2006 | 5 | 4 | 130 |
| 27 | 2006 | 7 | 657 | 212 |
| 28 | 2006 | 9 | 786 | 250 |
| 29 | 2006 | 10 | 484 | 296 |
| 32 | 2007 | 5 | 57 | 143 |
| 33 | 2007 | 6 | 393 | 177 |
| 34 | 2007 | 8 | 1006 | 234 |
| 36 | 2007 | 10 | 957 | 275 |
| 37 | 2007 | 11 | 108 | 328 |
| 39 | 2008 | 4 | 16 | 120 |
| 40 | 2008 | 7 | 606 | 183 |
| 42 | 2008 | 9 | 621 | 251 |
| 43 | 2008 | 9 | 502 | 267 |
| 44 | 2008 | 11 | 173 | 315 |
| 46 | 2009 | 5 | 5 | 126 |
| 47 | 2009 | 6 | 335 | 176 |
| 48 | 2009 | 9 | 705 | 253 |
| 49 | 2010 | 4 | 6 | 117 |
| 50 | 2010 | 10 | 662 | 274 |
| 51 | 2011 | 5 | 14 | 133 |
| 52 | 2011 | 8 | 1262 | 228 |
| 53 | 2011 | 10 | 284 | 278 |
| 54 | 2012 | 5 | 58 | 144 |
| 55 | 2012 | 8 | 802 | 215 |
| 56 | 2013 | 5 | 50 | 138 |
| 57 | 2013 | 7 | 1143 | 207 |
| 58 | 2013 | 9 | 909 | 249 |
| 59 | 2013 | 11 | 161 | 317 |
| 60 | 2014 | 4 | 1 | 113 |
| 61 | 2014 | 6 | 253 | 170 |
| 62 | 2014 | 10 | 468 | 281 |
| 63 | 2015 | 4 | 3 | 113 |
| 64 | 2015 | 6 | 112 | 159 |
| 65 | 2015 | 9 | 557 | 257 |
| 66 | 2015 | 10 | 278 | 275 |
| 67 | 2015 | 11 | 102 | 322 |
| 68 | 2016 | 8 | 725 | 222 |
| 69 | 2016 | 9 | 439 | 265 |
| 70 | 2016 | 10 | 112 | 301 |
| 71 | 2017 | 7 | 542 | 188 |
| 72 | 2017 | 9 | 838 | 247 |
| 73 | 2017 | 10 | 246 | 294 |
| 74 | 2018 | 5 | 39 | 143 |
| 75 | 2018 | 8 | 1079 | 213 |
| 76 | 2018 | 8 | 1605 | 243 |
| 77 | 2019 | 7 | 301 | 189 |
| 78 | 2019 | 8 | 1077 | 237 |
| 79 | 2019 | 10 | 362 | 275 |

Table S3. Estimated abundance ($A_{y}$: accumulated number of right whales) and log-scale variance-covariance matrix from the two stage regression model. Regression parameters were estimated as follows intercept $a=-13.26 (0.66 SE)$, julian day $c=0.16 (0.0060 SE)$, and Julian day^2 $d=-0.00034 (0.000013 SE)$. Year specific regression parameters ($b_{y}$) are given below.

|  |  |  |  | $\boldsymbol{\Sigma}$ | | | | | | | | | | | | | | | | |
| --- | --- | --- | --- | --- | --- | --- | --- | --- | --- | --- | --- | --- | --- | --- | --- | --- | --- | --- | --- | --- |
| Year | $b_{y}$ | $A_{y}$ |  | 1999 | 2000 | 2005 | 2006 | 2007 | 2008 | 2009 | 2010 | 2011 | 2012 | 2013 | 2014 | 2015 | 2016 | 2017 | 2018 | 2019 |
| 1999 | 0 | 956 |  | 0.0261 | 0.0015 | 0.0016 | 0.0007 | 0.0014 | 0.0013 | 0.0009 | 0.0019 | 0.0008 | 0.0008 | 0.0012 | 0.0015 | 0.0015 | 0.0004 | 0.0002 | 0.0001 | 0.0004 |
| 2000 | 0.666 (0.239) | 1,868 |  | 0.0015 | 0.0339 | 0.0011 | 0.0007 | 0.0013 | 0.0011 | 0.0011 | 0.0017 | 0.0006 | 0.0008 | 0.0009 | 0.0017 | 0.0014 | 0.0001 | 0.0001 | 0.0004 | 0.0000 |
| 2005 | 0.219 (0.229) | 1,193 |  | 0.0016 | 0.0011 | 0.0294 | 0.0007 | 0.0011 | 0.0011 | 0.0005 | 0.0015 | 0.0007 | 0.0006 | 0.0012 | 0.0013 | 0.0014 | 0.0002 | 0.0001 | 0.0003 | 0.0000 |
| 2006 | 0.428 (0.243) | 1,473 |  | 0.0007 | 0.0007 | 0.0007 | 0.0346 | 0.0009 | 0.0007 | 0.0010 | 0.0011 | 0.0005 | 0.0008 | 0.0007 | 0.0012 | 0.0009 | 0.0001 | 0.0002 | 0.0006 | 0.0002 |
| 2007 | 0.85 (0.225) | 2,237 |  | 0.0014 | 0.0013 | 0.0011 | 0.0009 | 0.0272 | 0.0010 | 0.0009 | 0.0015 | 0.0007 | 0.0011 | 0.0012 | 0.0015 | 0.0014 | 0.0002 | 0.0003 | 0.0004 | 0.0000 |
| 2008 | 0.714 (0.229) | 1,953 |  | 0.0013 | 0.0011 | 0.0011 | 0.0007 | 0.0010 | 0.0289 | 0.0008 | 0.0012 | 0.0006 | 0.0005 | 0.0009 | 0.0010 | 0.0012 | 0.0002 | 0.0003 | 0.0002 | 0.0001 |
| 2009 | 0.437 (0.275) | 1,499 |  | 0.0009 | 0.0011 | 0.0005 | 0.0010 | 0.0009 | 0.0008 | 0.0515 | 0.0013 | 0.0010 | 0.0024 | 0.0009 | 0.0018 | 0.0009 | 0.0004 | 0.0003 | 0.0013 | 0.0006 |
| 2010 | 0.528 (0.337) | 1,676 |  | 0.0019 | 0.0017 | 0.0015 | 0.0011 | 0.0015 | 0.0012 | 0.0013 | 0.0910 | 0.0010 | 0.0020 | 0.0016 | 0.0016 | 0.0017 | 0.0002 | 0.0003 | 0.0009 | 0.0003 |
| 2011 | 0.364 (0.269) | 1,387 |  | 0.0008 | 0.0006 | 0.0007 | 0.0005 | 0.0007 | 0.0006 | 0.0010 | 0.0010 | 0.0477 | 0.0010 | 0.0006 | 0.0009 | 0.0008 | 0.0001 | 0.0003 | 0.0004 | 0.0001 |
| 2012 | 0.684 (0.308) | 1,936 |  | 0.0008 | 0.0008 | 0.0006 | 0.0008 | 0.0011 | 0.0005 | 0.0024 | 0.0020 | 0.0010 | 0.0700 | 0.0013 | 0.0019 | 0.0009 | 0.0008 | 0.0001 | 0.0017 | 0.0003 |
| 2013 | 0.847 (0.24) | 2,238 |  | 0.0012 | 0.0009 | 0.0012 | 0.0007 | 0.0012 | 0.0009 | 0.0009 | 0.0016 | 0.0006 | 0.0013 | 0.0340 | 0.0012 | 0.0012 | 0.0001 | 0.0001 | 0.0003 | 0.0001 |
| 2014 | 0.495 (0.283) | 1,592 |  | 0.0015 | 0.0017 | 0.0013 | 0.0012 | 0.0015 | 0.0010 | 0.0018 | 0.0016 | 0.0009 | 0.0019 | 0.0012 | 0.0571 | 0.0014 | 0.0000 | 0.0003 | 0.0009 | 0.0001 |
| 2015 | 0.253 (0.235) | 1,234 |  | 0.0015 | 0.0014 | 0.0014 | 0.0009 | 0.0014 | 0.0012 | 0.0009 | 0.0017 | 0.0008 | 0.0009 | 0.0012 | 0.0014 | 0.0321 | 0.0004 | 0.0005 | 0.0002 | 0.0003 |
| 2016 | -0.009 (0.261) | 954 |  | 0.0004 | 0.0001 | 0.0002 | 0.0001 | 0.0002 | 0.0002 | 0.0004 | 0.0002 | 0.0001 | 0.0008 | 0.0001 | 0.0000 | 0.0004 | 0.0432 | 0.0001 | 0.0005 | 0.0001 |
| 2017 | 0.49 (0.261) | 1,570 |  | 0.0002 | 0.0001 | 0.0001 | 0.0002 | 0.0003 | 0.0003 | 0.0003 | 0.0003 | 0.0003 | 0.0001 | 0.0001 | 0.0003 | 0.0005 | 0.0001 | 0.0422 | 0.0000 | 0.0000 |
| 2018 | 0.824 (0.265) | 2,201 |  | 0.0001 | 0.0004 | 0.0003 | 0.0006 | 0.0004 | 0.0002 | 0.0013 | 0.0009 | 0.0004 | 0.0017 | 0.0003 | 0.0009 | 0.0002 | 0.0005 | 0.0000 | 0.0445 | 0.0004 |
| 2019 | 0.293 (0.263) | 1,291 |  | 0.0004 | 0.0000 | 0.0000 | 0.0002 | 0.0000 | 0.0001 | 0.0006 | 0.0003 | 0.0001 | 0.0003 | 0.0001 | 0.0001 | 0.0003 | 0.0001 | 0.0000 | 0.0004 | 0.0422 |

Table S4. Estimable parameters and prior specifications for considered in the sensitivity analyses (Scens 1-14).

| Sensitivity | $R_{max}$ | $\sigma^{2}$ | $Pmsy$ | SLR | Catch | $N_{recent}$ | Q | N-haplotypes |
| --- | --- | --- | --- | --- | --- | --- | --- | --- |
| Base | $runif(0, 0.11)$ | $runif\left( 1, 10 \right)*0.0000651$ | *unif(0.5, 0.8)* | Include | $unif(Low, High)$ | 2019 U[100, 10,000] | Analytical | 24 |
| 1 | $\boldsymbol{rlnom(-2.67, 0.5)}$ | $runif\left( 1, 10 \right)*0.0000651$ | *unif(0.5, 0.8)* | Include | $unif(Low, High)$ | 2019 U[100, 10,000] | Analytical | 24 |
| 2 | $\boldsymbol{rlnom(-2.67, 0.3)}$ | $runif\left( 1, 10 \right)*0.0000651$ | *unif(0.5, 0.8)* | Include | $unif(Low, High)$ | 2019 U[100, 10,000] | Analytical | 24 |
| 3 | $\boldsymbol{rlnom}\left( \boldsymbol{-2.67, 0.5} \right)$  $\boldsymbol{T(0.02,0.11)}$ | $runif\left( 1, 10 \right)*0.0000651$ | *unif(0.5, 0.8)* | Include | $unif(Low, High)$ | 2019 U[100, 10,000] | Analytical | 24 |
| 4 | $runif(0, 0.11)$ | $\boldsymbol{runif}\left( \boldsymbol{1, 100} \right)\boldsymbol{*0.0000651}$ | *unif(0.5, 0.8)* | Include | $unif(Low, High)$ | 2019 U[100, 10,000] | Analytical | 24 |
| 5 | $runif(0, 0.11)$ | $\boldsymbol{runif}\left( \boldsymbol{1, 2} \right)\boldsymbol{*0.0000651}$ | *unif(0.5, 0.8)* | Include | $unif(Low, High)$ | 2019 U[100, 10,000] | Analytical | 24 |
| 6 | $runif(0, 0.11)$ | $runif\left( 1, 10 \right)*0.0000651$ | *unif(0.5, 0.8)* | Include | $unif(Low, High)$ | **2004 U[100, 10,000]** | Analytical | 24 |
| 7 | $runif(0, 0.11)$ | $runif\left( 1, 10 \right)*0.0000651$ | *unif(0.5, 0.8)* | **Exclude** | $unif(Low, High)$ | 2019 U[100, 10,000] | Analytical | 24 |
| 8 | $runif(0, 0.11)$ | $runif\left( 1, 10 \right)*0.0000651$ | *unif(0.5, 0.8)* | Include | $\boldsymbol{low}$ | 2019 U[100, 10,000] | Analytical | 24 |
| 9 | $runif(0, 0.11)$ | $runif\left( 1, 10 \right)*0.0000651$ | *unif(0.5, 0.8)* | Include | $\boldsymbol{high}$ | 2019 U[100, 10,000] | Analytical | 24 |
| 10 | $runif(0, 0.11)$ | $runif\left( 1, 10 \right)*0.0000651$ | *unif(0.5, 0.8)* | Include | $unif(Low, High)$ | 2019 U[100, 10,000] | Analytical | **0** |
| 11 | $runif(0, 0.11)$ | $runif\left( 1, 10 \right)*0.0000651$ | *unif(0.5, 0.8)* | Include | $unif(Low, High)$ | 2019 U[100, 10,000] | Analytical | **25** |
| 12 | $runif(0, 0.11)$ | $runif\left( 1, 10 \right)*0.0000651$ | *unif(0.5, 0.8)* | Include | $unif(Low, High)$ | 2019 U[100, 10,000] | Analytical | **37** |
| 13 | $runif(0, 0.11)$ | $runif\left( 1, 10 \right)*0.0000651$ | *unif(0.5, 0.8)* | Include | $unif(Low, High)$ | 2019 U[100, 10,000] | **Analytical + additional obs error** | 24 |
| 14 | $runif(0, 0.11)$ | $runif\left( 1, 10 \right)*0.0000651$ | *unif(0.5, 0.8)* | Include | $unif(Low, High)$ | 2019 U[100, 10,000] | **Analytical + power function** | 24 |

Table S5. Posterior mean, standard deviations and 50% and 95% Bayesian credible intervals (CI) for the key biological parameters estimated by the individual state-space assessment models of the southern right whale *Eubalaena australis*. $P_{Min}$ refers to the minimum estimated abundance relative to $K$. $\hat{q}$ refers to the distribution of analytical catchability across posterior draws while $q$ refers to the posterior distribution of catchability.

**S5.0 Model average**

|  | Mean | Median | 2.5% | 25% | 75% | 97.5% |
| --- | --- | --- | --- | --- | --- | --- |
| $R_{max}$ | 0.014 | 0.012 | 0.001 | 0.006 | 0.02 | 0.034 |
| $K$ | 60,682 | 58,212 | 33,329 | 48,271 | 70,580 | 100,920 |
| $z$ | 4.245 | 3.408 | 1.066 | 1.903 | 6.052 | 10.56 |
| $P_{MSY}$ | 0.648 | 0.647 | 0.506 | 0.571 | 0.724 | 0.793 |
| $\sigma$ | 0.0189 | 0.0197 | 0.0091 | 0.0155 | 0.0229 | 0.0253 |
| $N_{Min}$ | 1,958 | 1,829 | 355 | 969 | 2,919 | 3,969 |
| $N_{2019}$ | 4,667 | 4,634 | 3,857 | 4,333 | 4,964 | 5,668 |
| $N_{2021}$ | 4,796 | 4,742 | 3,853 | 4,400 | 5,143 | 6,013 |
| $N_{2030}$ | 5,466 | 5,299 | 3,886 | 4,698 | 6,079 | 7,931 |
| $P_{Min}$ | 0.032 | 0.03 | 0.008 | 0.019 | 0.042 | 0.067 |
| $P_{2019}$ | 0.084 | 0.08 | 0.042 | 0.064 | 0.1 | 0.152 |
| $P_{2021}$ | 0.087 | 0.082 | 0.042 | 0.064 | 0.103 | 0.16 |
| $P_{2030}$ | 0.1 | 0.091 | 0.043 | 0.069 | 0.122 | 0.205 |
| $\hat{q}$ | 0.383 | 0.362 | 0.207 | 0.345 | 0.38 | 0.717 |
| $q$ | 0.401 | 0.359 | 0.158 | 0.276 | 0.472 | 0.809 |
| $\beta$ | -0.001 | 0 | -0.081 | 0 | 0 | 0.067 |
| $\tau^{2}$ | 0.046 | 0 | 0 | 0 | 0 | 0.529 |

**S5.1 Base Case**

|  | Mean | Median | 2.5% | 25% | 75% | 97.5% |
| --- | --- | --- | --- | --- | --- | --- |
| $R_{max}$ | 0.011 | 0.01 | 0.001 | 0.006 | 0.016 | 0.028 |
| $K$ | 65,222 | 62,661 | 40,628 | 53,071 | 74,703 | 103,269 |
| $z$ | 4.276 | 3.474 | 1.072 | 1.957 | 6.095 | 10.491 |
| $P_{MSY}$ | 0.65 | 0.65 | 0.507 | 0.575 | 0.725 | 0.792 |
| $\sigma$ | 0.0187 | 0.0195 | 0.0091 | 0.0151 | 0.0228 | 0.0252 |
| $N_{Min}$ | 2,185 | 2,167 | 517 | 1,288 | 3,049 | 3,991 |
| $N_{2019}$ | 4,593 | 4,567 | 3,819 | 4,289 | 4,868 | 5,521 |
| $N_{2021}$ | 4,702 | 4,664 | 3,830 | 4,351 | 5,016 | 5,801 |
| $N_{2030}$ | 5,242 | 5,120 | 3,871 | 4,602 | 5,749 | 7,259 |
| $P_{Min}$ | 0.033 | 0.032 | 0.011 | 0.022 | 0.042 | 0.062 |
| $P_{2019}$ | 0.075 | 0.073 | 0.041 | 0.059 | 0.088 | 0.125 |
| $P_{2021}$ | 0.077 | 0.074 | 0.041 | 0.06 | 0.091 | 0.131 |
| $P_{2030}$ | 0.087 | 0.082 | 0.042 | 0.064 | 0.104 | 0.163 |
| $\hat{q}$ | 0.362 | 0.362 | 0.319 | 0.346 | 0.377 | 0.409 |
| $q$ | 0.379 | 0.358 | 0.185 | 0.28 | 0.462 | 0.668 |
| $\beta$ | 0 | 0 | 0 | 0 | 0 | 0 |
| $\tau^{2}$ | 0 | 0 | 0 | 0 | 0 | 0 |

**S5.2 Scen 1**

|  | Mean | Median | 2.5% | 25% | 75% | 97.5% |
| --- | --- | --- | --- | --- | --- | --- |
| $R_{max}$ | 0.024 | 0.023 | 0.013 | 0.019 | 0.028 | 0.037 |
| $K$ | 51,372 | 50,155 | 34,875 | 43,593 | 57,933 | 74,232 |
| $z$ | 4.176 | 3.338 | 1.058 | 1.836 | 5.975 | 10.539 |
| $P_{MSY}$ | 0.646 | 0.644 | 0.506 | 0.567 | 0.722 | 0.793 |
| $\sigma$ | 0.0195 | 0.0206 | 0.0094 | 0.0164 | 0.0234 | 0.0253 |
| $N_{Min}$ | 838 | 753 | 270 | 539 | 1,046 | 1,894 |
| $N_{2019}$ | 4,982 | 4,961 | 4,140 | 4,669 | 5,276 | 5,933 |
| $N_{2021}$ | 5,224 | 5,199 | 4,242 | 4,860 | 5,559 | 6,353 |
| $N_{2030}$ | 6,469 | 6,379 | 4,795 | 5,777 | 7,074 | 8,630 |
| $P_{Min}$ | 0.016 | 0.015 | 0.007 | 0.011 | 0.019 | 0.031 |
| $P_{2019}$ | 0.101 | 0.099 | 0.062 | 0.084 | 0.116 | 0.154 |
| $P_{2021}$ | 0.106 | 0.103 | 0.064 | 0.087 | 0.122 | 0.165 |
| $P_{2030}$ | 0.132 | 0.127 | 0.073 | 0.104 | 0.155 | 0.223 |
| $\hat{q}$ | 0.362 | 0.361 | 0.319 | 0.346 | 0.377 | 0.41 |
| $q$ | 0.379 | 0.356 | 0.179 | 0.279 | 0.462 | 0.675 |
| $\beta$ | 0 | 0 | 0 | 0 | 0 | 0 |
| $\tau^{2}$ | 0 | 0 | 0 | 0 | 0 | 0 |

**S5.3 Scen 2**

|  | Mean | Median | 2.5% | 25% | 75% | 97.5% |
| --- | --- | --- | --- | --- | --- | --- |
| $R_{max}$ | 0.032 | 0.032 | 0.022 | 0.028 | 0.036 | 0.045 |
| $K$ | 45,518 | 44,255 | 31,869 | 39,076 | 51,150 | 65,183 |
| $z$ | 3.939 | 3.028 | 1.041 | 1.655 | 5.607 | 10.398 |
| $P_{MSY}$ | 0.636 | 0.631 | 0.504 | 0.554 | 0.714 | 0.791 |
| $\sigma$ | 0.0204 | 0.0215 | 0.0103 | 0.018 | 0.0238 | 0.0253 |
| $N_{Min}$ | 474 | 445 | 182 | 334 | 580 | 934 |
| $N_{2019}$ | 5,253 | 5,227 | 4,397 | 4,927 | 5,559 | 6,245 |
| $N_{2021}$ | 5,597 | 5,565 | 4,596 | 5,213 | 5,952 | 6,774 |
| $N_{2030}$ | 7,453 | 7,370 | 5,599 | 6,699 | 8,106 | 9,834 |
| $P_{Min}$ | 0.01 | 0.01 | 0.005 | 0.008 | 0.012 | 0.018 |
| $P_{2019}$ | 0.12 | 0.118 | 0.075 | 0.101 | 0.136 | 0.178 |
| $P_{2021}$ | 0.128 | 0.125 | 0.078 | 0.107 | 0.146 | 0.193 |
| $P_{2030}$ | 0.171 | 0.165 | 0.097 | 0.137 | 0.199 | 0.277 |
| $\hat{q}$ | 0.362 | 0.361 | 0.318 | 0.346 | 0.377 | 0.408 |
| $q$ | 0.379 | 0.356 | 0.174 | 0.278 | 0.462 | 0.687 |
| $\beta$ | 0 | 0 | 0 | 0 | 0 | 0 |
| $\tau^{2}$ | 0 | 0 | 0 | 0 | 0 | 0 |

**S5.4 Scen 3**

|  | Mean | Median | 2.5% | 25% | 75% | 97.5% |
| --- | --- | --- | --- | --- | --- | --- |
| $R_{max}$ | 0.027 | 0.026 | 0.02 | 0.023 | 0.029 | 0.038 |
| $K$ | 48,961 | 47,787 | 34,149 | 42,003 | 54,824 | 69,502 |
| $z$ | 4.097 | 3.26 | 1.056 | 1.78 | 5.832 | 10.467 |
| $P_{MSY}$ | 0.643 | 0.641 | 0.505 | 0.563 | 0.719 | 0.792 |
| $\sigma$ | 0.0197 | 0.0208 | 0.0096 | 0.0168 | 0.0235 | 0.0253 |
| $N_{Min}$ | 645 | 628 | 254 | 474 | 796 | 1,125 |
| $N_{2019}$ | 5,080 | 5,063 | 4,252 | 4,772 | 5,368 | 6,001 |
| $N_{2021}$ | 5,353 | 5,329 | 4,418 | 4,995 | 5,674 | 6,427 |
| $N_{2030}$ | 6,781 | 6,679 | 5,220 | 6,138 | 7,322 | 8,877 |
| $P_{Min}$ | 0.013 | 0.013 | 0.006 | 0.01 | 0.015 | 0.021 |
| $P_{2019}$ | 0.108 | 0.105 | 0.069 | 0.091 | 0.122 | 0.159 |
| $P_{2021}$ | 0.114 | 0.111 | 0.071 | 0.095 | 0.129 | 0.17 |
| $P_{2030}$ | 0.144 | 0.139 | 0.086 | 0.117 | 0.165 | 0.233 |
| $\hat{q}$ | 0.362 | 0.361 | 0.319 | 0.346 | 0.377 | 0.408 |
| $q$ | 0.378 | 0.356 | 0.178 | 0.278 | 0.462 | 0.679 |
| $\beta$ | 0 | 0 | 0 | 0 | 0 | 0 |
| $\tau^{2}$ | 0 | 0 | 0 | 0 | 0 | 0 |

**S5.5 Scen 4**

|  | Mean | Median | 2.5% | 25% | 75% | 97.5% |
| --- | --- | --- | --- | --- | --- | --- |
| $R_{max}$ | 0.018 | 0.016 | 0.001 | 0.008 | 0.026 | 0.048 |
| $K$ | 68,352 | 58,217 | 31,205 | 45,791 | 77,736 | 165,051 |
| $z$ | 4.361 | 3.538 | 1.059 | 1.928 | 6.442 | 10.46 |
| $P_{MSY}$ | 0.651 | 0.652 | 0.506 | 0.573 | 0.732 | 0.792 |
| $\sigma$ | 0.0619 | 0.0657 | 0.0243 | 0.0532 | 0.0744 | 0.08 |
| $N_{Min}$ | 1,279 | 1,056 | 163 | 510 | 1,868 | 3,320 |
| $N_{2019}$ | 4,251 | 4,215 | 3,134 | 3,809 | 4,647 | 5,576 |
| $N_{2021}$ | 4,449 | 4,389 | 3,061 | 3,925 | 4,911 | 6,168 |
| $N_{2030}$ | 5,391 | 5,139 | 2,938 | 4,247 | 6,191 | 9,700 |
| $P_{Min}$ | 0.02 | 0.016 | 0.004 | 0.01 | 0.026 | 0.056 |
| $P_{2019}$ | 0.076 | 0.072 | 0.024 | 0.053 | 0.094 | 0.148 |
| $P_{2021}$ | 0.08 | 0.075 | 0.024 | 0.054 | 0.101 | 0.164 |
| $P_{2030}$ | 0.099 | 0.087 | 0.024 | 0.058 | 0.123 | 0.257 |
| $\hat{q}$ | 0.381 | 0.379 | 0.318 | 0.357 | 0.404 | 0.452 |
| $q$ | 0.395 | 0.373 | 0.197 | 0.296 | 0.474 | 0.702 |
| $\beta$ | 0 | 0 | 0 | 0 | 0 | 0 |
| $\tau^{2}$ | 0 | 0 | 0 | 0 | 0 | 0 |

**S5.6 Scen 5**

|  | Mean | Median | 2.5% | 25% | 75% | 97.5% |
| --- | --- | --- | --- | --- | --- | --- |
| $R_{max}$ | 0.011 | 0.01 | 0.001 | 0.005 | 0.015 | 0.025 |
| $K$ | 65,401 | 63,721 | 42,267 | 54,251 | 74,884 | 98,081 |
| $z$ | 4.285 | 3.481 | 1.074 | 1.957 | 6.148 | 10.487 |
| $P_{MSY}$ | 0.65 | 0.65 | 0.507 | 0.575 | 0.726 | 0.792 |
| $\sigma$ | 0.0098 | 0.0099 | 0.0082 | 0.009 | 0.0107 | 0.0113 |
| $N_{Min}$ | 2,380 | 2,407 | 599 | 1,450 | 3,288 | 4,135 |
| $N_{2019}$ | 4,654 | 4,625 | 3,958 | 4,374 | 4,906 | 5,502 |
| $N_{2021}$ | 4,756 | 4,717 | 3,978 | 4,433 | 5,038 | 5,746 |
| $N_{2030}$ | 5,257 | 5,146 | 4,058 | 4,680 | 5,711 | 7,097 |
| $P_{Min}$ | 0.036 | 0.036 | 0.013 | 0.025 | 0.045 | 0.062 |
| $P_{2019}$ | 0.075 | 0.073 | 0.044 | 0.06 | 0.088 | 0.122 |
| $P_{2021}$ | 0.077 | 0.074 | 0.044 | 0.061 | 0.09 | 0.127 |
| $P_{2030}$ | 0.086 | 0.081 | 0.044 | 0.065 | 0.102 | 0.157 |
| $\hat{q}$ | 0.359 | 0.359 | 0.32 | 0.345 | 0.373 | 0.402 |
| $q$ | 0.376 | 0.356 | 0.183 | 0.277 | 0.46 | 0.663 |
| $\beta$ | 0 | 0 | 0 | 0 | 0 | 0 |
| $\tau^{2}$ | 0 | 0 | 0 | 0 | 0 | 0 |

**S5.7 Scen 6**

|  | Mean | Median | 2.5% | 25% | 75% | 97.5% |
| --- | --- | --- | --- | --- | --- | --- |
| $R_{max}$ | 0.011 | 0.01 | 0.001 | 0.005 | 0.015 | 0.027 |
| $K$ | 66,158 | 63,547 | 40,680 | 53,688 | 75,704 | 107,014 |
| $z$ | 4.247 | 3.443 | 1.073 | 1.925 | 6.043 | 10.526 |
| $P_{MSY}$ | 0.649 | 0.648 | 0.507 | 0.573 | 0.724 | 0.793 |
| $\sigma$ | 0.0188 | 0.0196 | 0.0092 | 0.0153 | 0.0229 | 0.0253 |
| $N_{Min}$ | 2,251 | 2,257 | 533 | 1,347 | 3,120 | 4,039 |
| $N_{2019}$ | 3,997 | 3,985 | 3,399 | 3,781 | 4,204 | 4,642 |
| $N_{2021}$ | 4,677 | 4,644 | 3,807 | 4,333 | 4,988 | 5,764 |
| $N_{2030}$ | 5,194 | 5,083 | 3,819 | 4,576 | 5,698 | 7,209 |
| $P_{Min}$ | 0.034 | 0.033 | 0.011 | 0.023 | 0.043 | 0.063 |
| $P_{2019}$ | 0.064 | 0.063 | 0.038 | 0.053 | 0.074 | 0.097 |
| $P_{2021}$ | 0.076 | 0.073 | 0.04 | 0.059 | 0.089 | 0.129 |
| $P_{2030}$ | 0.085 | 0.08 | 0.04 | 0.062 | 0.102 | 0.161 |
| $\hat{q}$ | 0.362 | 0.361 | 0.32 | 0.346 | 0.377 | 0.409 |
| $q$ | 0.379 | 0.358 | 0.185 | 0.28 | 0.461 | 0.667 |
| $\beta$ | 0 | 0 | 0 | 0 | 0 | 0 |
| $\tau^{2}$ | 0 | 0 | 0 | 0 | 0 | 0 |

**S5.8 Scen 7**

|  | Mean | Median | 2.5% | 25% | 75% | 97.5% |
| --- | --- | --- | --- | --- | --- | --- |
| $R_{max}$ | 0.012 | 0.011 | 0.001 | 0.006 | 0.016 | 0.028 |
| $K$ | 44,325 | 42,598 | 26,104 | 35,130 | 51,283 | 73,576 |
| $z$ | 4.22 | 3.418 | 1.07 | 1.923 | 6.009 | 10.487 |
| $P_{MSY}$ | 0.648 | 0.647 | 0.507 | 0.572 | 0.723 | 0.792 |
| $\sigma$ | 0.0188 | 0.0196 | 0.0091 | 0.0152 | 0.0229 | 0.0253 |
| $N_{Min}$ | 2,092 | 2,004 | 447 | 1,165 | 2,992 | 3,991 |
| $N_{2019}$ | 4,595 | 4,564 | 3,823 | 4,291 | 4,868 | 5,523 |
| $N_{2021}$ | 4,705 | 4,665 | 3,823 | 4,356 | 5,010 | 5,810 |
| $N_{2030}$ | 5,249 | 5,138 | 3,858 | 4,614 | 5,755 | 7,296 |
| $P_{Min}$ | 0.046 | 0.044 | 0.015 | 0.03 | 0.06 | 0.092 |
| $P_{2019}$ | 0.112 | 0.108 | 0.057 | 0.086 | 0.133 | 0.193 |
| $P_{2021}$ | 0.115 | 0.11 | 0.058 | 0.088 | 0.137 | 0.202 |
| $P_{2030}$ | 0.13 | 0.121 | 0.059 | 0.093 | 0.157 | 0.251 |
| $\hat{q}$ | 0.362 | 0.362 | 0.32 | 0.347 | 0.377 | 0.41 |
| $q$ | 0.379 | 0.358 | 0.185 | 0.28 | 0.462 | 0.669 |
| $\beta$ | 0 | 0 | 0 | 0 | 0 | 0 |
| $\tau^{2}$ | 0 | 0 | 0 | 0 | 0 | 0 |

**S5.9 Scen 8**

|  | Mean | Median | 2.5% | 25% | 75% | 97.5% |
| --- | --- | --- | --- | --- | --- | --- |
| $R_{max}$ | 0.012 | 0.011 | 0.001 | 0.006 | 0.016 | 0.028 |
| $K$ | 48,695 | 47,251 | 35,532 | 42,559 | 53,021 | 70,792 |
| $z$ | 4.231 | 3.4 | 1.067 | 1.917 | 6.047 | 10.555 |
| $P_{MSY}$ | 0.648 | 0.647 | 0.506 | 0.572 | 0.724 | 0.793 |
| $\sigma$ | 0.0188 | 0.0196 | 0.0092 | 0.0153 | 0.0229 | 0.0252 |
| $N_{Min}$ | 1,908 | 1,729 | 285 | 905 | 2,905 | 3,998 |
| $N_{2019}$ | 4,595 | 4,575 | 3,821 | 4,297 | 4,869 | 5,504 |
| $N_{2021}$ | 4,706 | 4,672 | 3,832 | 4,356 | 5,021 | 5,766 |
| $N_{2030}$ | 5,250 | 5,139 | 3,867 | 4,616 | 5,762 | 7,262 |
| $P_{Min}$ | 0.038 | 0.036 | 0.007 | 0.021 | 0.053 | 0.076 |
| $P_{2019}$ | 0.098 | 0.096 | 0.06 | 0.083 | 0.111 | 0.145 |
| $P_{2021}$ | 0.1 | 0.098 | 0.06 | 0.084 | 0.115 | 0.153 |
| $P_{2030}$ | 0.113 | 0.108 | 0.06 | 0.089 | 0.132 | 0.194 |
| $\hat{q}$ | 0.362 | 0.362 | 0.32 | 0.347 | 0.377 | 0.409 |
| $q$ | 0.379 | 0.359 | 0.185 | 0.28 | 0.462 | 0.668 |
| $\beta$ | 0 | 0 | 0 | 0 | 0 | 0 |
| $\tau^{2}$ | 0 | 0 | 0 | 0 | 0 | 0 |

**S5.10 Scen 9**

|  | Mean | Median | 2.5% | 25% | 75% | 97.5% |
| --- | --- | --- | --- | --- | --- | --- |
| $R_{max}$ | 0.011 | 0.011 | 0.001 | 0.006 | 0.016 | 0.027 |
| $K$ | 82,367 | 80,169 | 54,382 | 70,069 | 91,694 | 124,377 |
| $z$ | 4.249 | 3.461 | 1.074 | 1.939 | 6.064 | 10.467 |
| $P_{MSY}$ | 0.649 | 0.649 | 0.507 | 0.574 | 0.724 | 0.792 |
| $\sigma$ | 0.0188 | 0.0196 | 0.0091 | 0.0153 | 0.0229 | 0.0252 |
| $N_{Min}$ | 2,346 | 2,346 | 750 | 1,555 | 3,101 | 4,005 |
| $N_{2019}$ | 4,595 | 4,566 | 3,816 | 4,294 | 4,866 | 5,518 |
| $N_{2021}$ | 4,706 | 4,665 | 3,835 | 4,352 | 5,020 | 5,803 |
| $N_{2030}$ | 5,251 | 5,132 | 3,853 | 4,613 | 5,759 | 7,287 |
| $P_{Min}$ | 0.028 | 0.028 | 0.012 | 0.021 | 0.034 | 0.045 |
| $P_{2019}$ | 0.059 | 0.057 | 0.034 | 0.048 | 0.068 | 0.094 |
| $P_{2021}$ | 0.06 | 0.058 | 0.034 | 0.048 | 0.07 | 0.099 |
| $P_{2030}$ | 0.068 | 0.064 | 0.034 | 0.051 | 0.081 | 0.125 |
| $\hat{q}$ | 0.362 | 0.361 | 0.319 | 0.347 | 0.377 | 0.409 |
| $q$ | 0.379 | 0.358 | 0.185 | 0.28 | 0.462 | 0.668 |
| $\beta$ | 0 | 0 | 0 | 0 | 0 | 0 |
| $\tau^{2}$ | 0 | 0 | 0 | 0 | 0 | 0 |

**S5.11 Scen 10**

|  | Mean | Median | 2.5% | 25% | 75% | 97.5% |
| --- | --- | --- | --- | --- | --- | --- |
| $R_{max}$ | 0.011 | 0.01 | 0.001 | 0.006 | 0.016 | 0.028 |
| $K$ | 65,449 | 63,149 | 40,708 | 53,472 | 74,640 | 103,802 |
| $z$ | 4.251 | 3.45 | 1.071 | 1.923 | 6.091 | 10.524 |
| $P_{MSY}$ | 0.649 | 0.649 | 0.507 | 0.573 | 0.725 | 0.793 |
| $\sigma$ | 0.0188 | 0.0196 | 0.0091 | 0.0152 | 0.0229 | 0.0253 |
| $N_{Min}$ | 2,198 | 2,185 | 524 | 1,294 | 3,070 | 4,029 |
| $N_{2019}$ | 4,589 | 4,563 | 3,813 | 4,286 | 4,863 | 5,510 |
| $N_{2021}$ | 4,698 | 4,662 | 3,818 | 4,344 | 5,014 | 5,774 |
| $N_{2030}$ | 5,235 | 5,119 | 3,836 | 4,609 | 5,737 | 7,260 |
| $P_{Min}$ | 0.033 | 0.032 | 0.011 | 0.022 | 0.042 | 0.062 |
| $P_{2019}$ | 0.075 | 0.072 | 0.041 | 0.059 | 0.088 | 0.125 |
| $P_{2021}$ | 0.077 | 0.074 | 0.041 | 0.06 | 0.09 | 0.131 |
| $P_{2030}$ | 0.087 | 0.081 | 0.042 | 0.064 | 0.103 | 0.163 |
| $\hat{q}$ | 0.363 | 0.362 | 0.32 | 0.346 | 0.378 | 0.41 |
| $q$ | 0.379 | 0.359 | 0.185 | 0.28 | 0.462 | 0.669 |
| $\beta$ | 0 | 0 | 0 | 0 | 0 | 0 |
| $\tau^{2}$ | 0 | 0 | 0 | 0 | 0 | 0 |

**S5.12 Scen 11**

|  | Mean | Median | 2.5% | 25% | 75% | 97.5% |
| --- | --- | --- | --- | --- | --- | --- |
| $R_{max}$ | 0.011 | 0.01 | 0.001 | 0.006 | 0.016 | 0.028 |
| $K$ | 65,380 | 63,131 | 40,420 | 53,235 | 74,846 | 103,595 |
| $z$ | 4.257 | 3.464 | 1.068 | 1.956 | 6.072 | 10.473 |
| $P_{MSY}$ | 0.649 | 0.649 | 0.506 | 0.575 | 0.725 | 0.792 |
| $\sigma$ | 0.0188 | 0.0195 | 0.0091 | 0.0153 | 0.0228 | 0.0253 |
| $N_{Min}$ | 2,186 | 2,151 | 509 | 1,300 | 3,040 | 3,996 |
| $N_{2019}$ | 4,594 | 4,571 | 3,824 | 4,297 | 4,867 | 5,488 |
| $N_{2021}$ | 4,704 | 4,669 | 3,829 | 4,356 | 5,018 | 5,760 |
| $N_{2030}$ | 5,241 | 5,125 | 3,850 | 4,619 | 5,760 | 7,214 |
| $P_{Min}$ | 0.033 | 0.032 | 0.011 | 0.022 | 0.042 | 0.062 |
| $P_{2019}$ | 0.075 | 0.072 | 0.041 | 0.059 | 0.088 | 0.125 |
| $P_{2021}$ | 0.077 | 0.074 | 0.041 | 0.06 | 0.091 | 0.131 |
| $P_{2030}$ | 0.087 | 0.081 | 0.042 | 0.064 | 0.104 | 0.163 |
| $\hat{q}$ | 0.362 | 0.362 | 0.32 | 0.347 | 0.377 | 0.409 |
| $q$ | 0.379 | 0.358 | 0.185 | 0.28 | 0.462 | 0.668 |
| $\beta$ | 0 | 0 | 0 | 0 | 0 | 0 |
| $\tau^{2}$ | 0 | 0 | 0 | 0 | 0 | 0 |

**S5.13 Scen 12**

|  | Mean | Median | 2.5% | 25% | 75% | 97.5% |
| --- | --- | --- | --- | --- | --- | --- |
| $R_{max}$ | 0.012 | 0.011 | 0.001 | 0.006 | 0.016 | 0.028 |
| $K$ | 65,365 | 62,894 | 40,794 | 52,886 | 74,926 | 104,918 |
| $z$ | 4.208 | 3.377 | 1.07 | 1.914 | 5.979 | 10.487 |
| $P_{MSY}$ | 0.648 | 0.646 | 0.507 | 0.572 | 0.723 | 0.792 |
| $\sigma$ | 0.0188 | 0.0195 | 0.0091 | 0.0152 | 0.0228 | 0.0253 |
| $N_{Min}$ | 2,169 | 2,128 | 502 | 1,267 | 3,051 | 3,999 |
| $N_{2019}$ | 4,595 | 4,568 | 3,821 | 4,289 | 4,867 | 5,504 |
| $N_{2021}$ | 4,707 | 4,672 | 3,833 | 4,357 | 5,017 | 5,774 |
| $N_{2030}$ | 5,250 | 5,125 | 3,861 | 4,621 | 5,767 | 7,267 |
| $P_{Min}$ | 0.033 | 0.032 | 0.011 | 0.022 | 0.042 | 0.062 |
| $P_{2019}$ | 0.075 | 0.073 | 0.04 | 0.059 | 0.089 | 0.124 |
| $P_{2021}$ | 0.077 | 0.074 | 0.041 | 0.06 | 0.091 | 0.131 |
| $P_{2030}$ | 0.087 | 0.082 | 0.041 | 0.064 | 0.104 | 0.163 |
| $\hat{q}$ | 0.362 | 0.362 | 0.32 | 0.347 | 0.378 | 0.409 |
| $q$ | 0.379 | 0.359 | 0.185 | 0.28 | 0.462 | 0.668 |
| $\beta$ | 0 | 0 | 0 | 0 | 0 | 0 |
| $\tau^{2}$ | 0 | 0 | 0 | 0 | 0 | 0 |

**S5.14 Scen 13**

|  | Mean | Median | 2.5% | 25% | 75% | 97.5% |
| --- | --- | --- | --- | --- | --- | --- |
| $R_{max}$ | 0.011 | 0.01 | 0.001 | 0.006 | 0.016 | 0.027 |
| $K$ | 65,219 | 62,711 | 40,336 | 53,069 | 74,676 | 104,121 |
| $z$ | 4.281 | 3.462 | 1.075 | 1.944 | 6.119 | 10.492 |
| $P_{MSY}$ | 0.65 | 0.649 | 0.507 | 0.574 | 0.726 | 0.792 |
| $\sigma$ | 0.0187 | 0.0195 | 0.0091 | 0.0152 | 0.0228 | 0.0252 |
| $N_{Min}$ | 2,193 | 2,166 | 505 | 1,290 | 3,071 | 4,015 |
| $N_{2019}$ | 4,591 | 4,565 | 3,822 | 4,289 | 4,866 | 5,499 |
| $N_{2021}$ | 4,700 | 4,662 | 3,836 | 4,353 | 5,008 | 5,776 |
| $N_{2030}$ | 5,237 | 5,115 | 3,864 | 4,601 | 5,740 | 7,274 |
| $P_{Min}$ | 0.033 | 0.032 | 0.011 | 0.022 | 0.042 | 0.063 |
| $P_{2019}$ | 0.075 | 0.073 | 0.041 | 0.059 | 0.089 | 0.125 |
| $P_{2021}$ | 0.077 | 0.074 | 0.041 | 0.06 | 0.091 | 0.133 |
| $P_{2030}$ | 0.087 | 0.082 | 0.041 | 0.064 | 0.104 | 0.165 |
| $\hat{q}$ | 0.363 | 0.362 | 0.32 | 0.347 | 0.377 | 0.409 |
| $q$ | 0.379 | 0.359 | 0.185 | 0.28 | 0.462 | 0.668 |
| $\beta$ | 0 | 0 | 0 | 0 | 0 | 0 |
| $\tau^{2}$ | 0.462 | 0.448 | 0.274 | 0.378 | 0.529 | 0.734 |

**S5.15 Scen 14**

|  | Mean | Median | 2.5% | 25% | 75% | 97.5% |
| --- | --- | --- | --- | --- | --- | --- |
| $R_{max}$ | 0.011 | 0.01 | 0.001 | 0.006 | 0.016 | 0.028 |
| $K$ | 65,265 | 62,694 | 40,554 | 52,985 | 74,762 | 105,519 |
| $z$ | 4.258 | 3.459 | 1.078 | 1.907 | 6.068 | 10.611 |
| $P_{MSY}$ | 0.649 | 0.649 | 0.507 | 0.571 | 0.724 | 0.794 |
| $\sigma$ | 0.0188 | 0.0196 | 0.0091 | 0.0153 | 0.0228 | 0.0252 |
| $N_{Min}$ | 2,179 | 2,165 | 503 | 1,267 | 3,053 | 4,003 |
| $N_{2019}$ | 4,594 | 4,563 | 3,828 | 4,293 | 4,868 | 5,529 |
| $N_{2021}$ | 4,703 | 4,657 | 3,832 | 4,348 | 5,014 | 5,805 |
| $N_{2030}$ | 5,248 | 5,121 | 3,874 | 4,608 | 5,756 | 7,374 |
| $P_{Min}$ | 0.033 | 0.032 | 0.011 | 0.022 | 0.042 | 0.062 |
| $P_{2019}$ | 0.075 | 0.073 | 0.04 | 0.059 | 0.089 | 0.125 |
| $P_{2021}$ | 0.077 | 0.074 | 0.041 | 0.06 | 0.091 | 0.132 |
| $P_{2030}$ | 0.087 | 0.082 | 0.041 | 0.064 | 0.104 | 0.166 |
| $\hat{q}$ | 0.551 | 0.387 | 0.073 | 0.216 | 0.678 | 2.016 |
| $q$ | 0.576 | 0.381 | 0.064 | 0.206 | 0.704 | 2.276 |
| $\beta$ | -0.007 | -0.008 | -0.207 | -0.075 | 0.061 | 0.192 |
| $\tau^{2}$ | 0 | 0 | 0 | 0 | 0 | 0 |

Table S6. Bayes factor comparison of scenarios. Blank indicates the model was not included in model averaging.

| Model | BayesFactor |
| --- | --- |
| B | 0.1085 |
| S-1 | 0.0687 |
| S-2 | 0.0323 |
| S-3 | 0.0518 |
| S-4 |  |
| S-5 |  |
| S-6 | 0.108 |
| S-7 | 0.1083 |
| S-8 |  |
| S-9 |  |
| S-10 | 0.1041 |
| S-11 | 0.1055 |
| S-12 | 0.1045 |
| S-13 | 0.1017 |
| S-14 | 0.1066 |

Figure S1. Trend of the observed (black dots) and estimated (grey dots) accumulated numbers of the southern right whale *Eubalaena australis* and associated 95% confidence interval (black bars) and 95% posterior predictive intervals (grey bars). The solid blue line represents the median estimated model-averaged trajectory of the population abundance ($N_{y}$) multiplied by posterior catchability (q), while the shaded areas correspond to the 50% and 95% credible intervals.

S1.1 Base Case


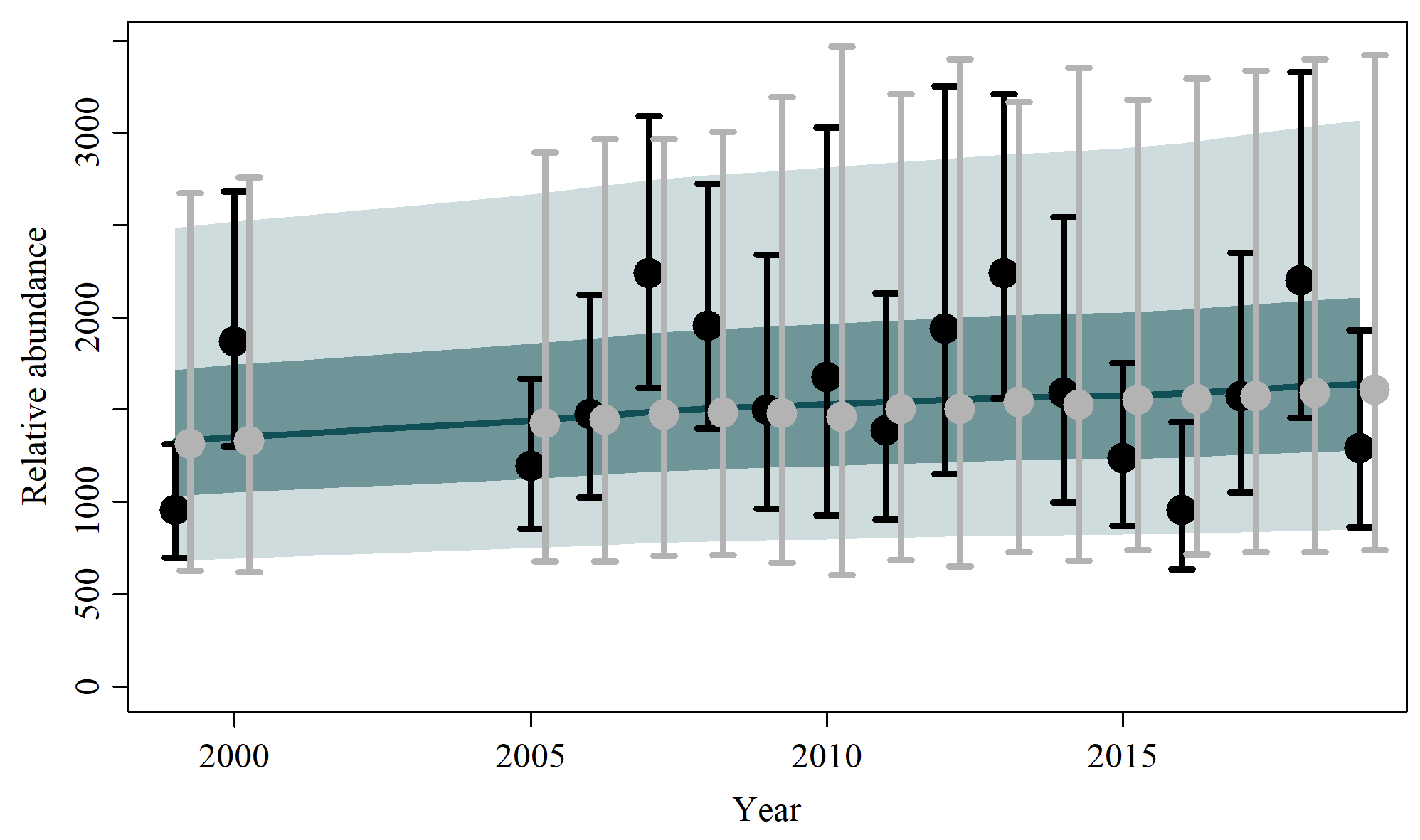


S1.2 Scen 1


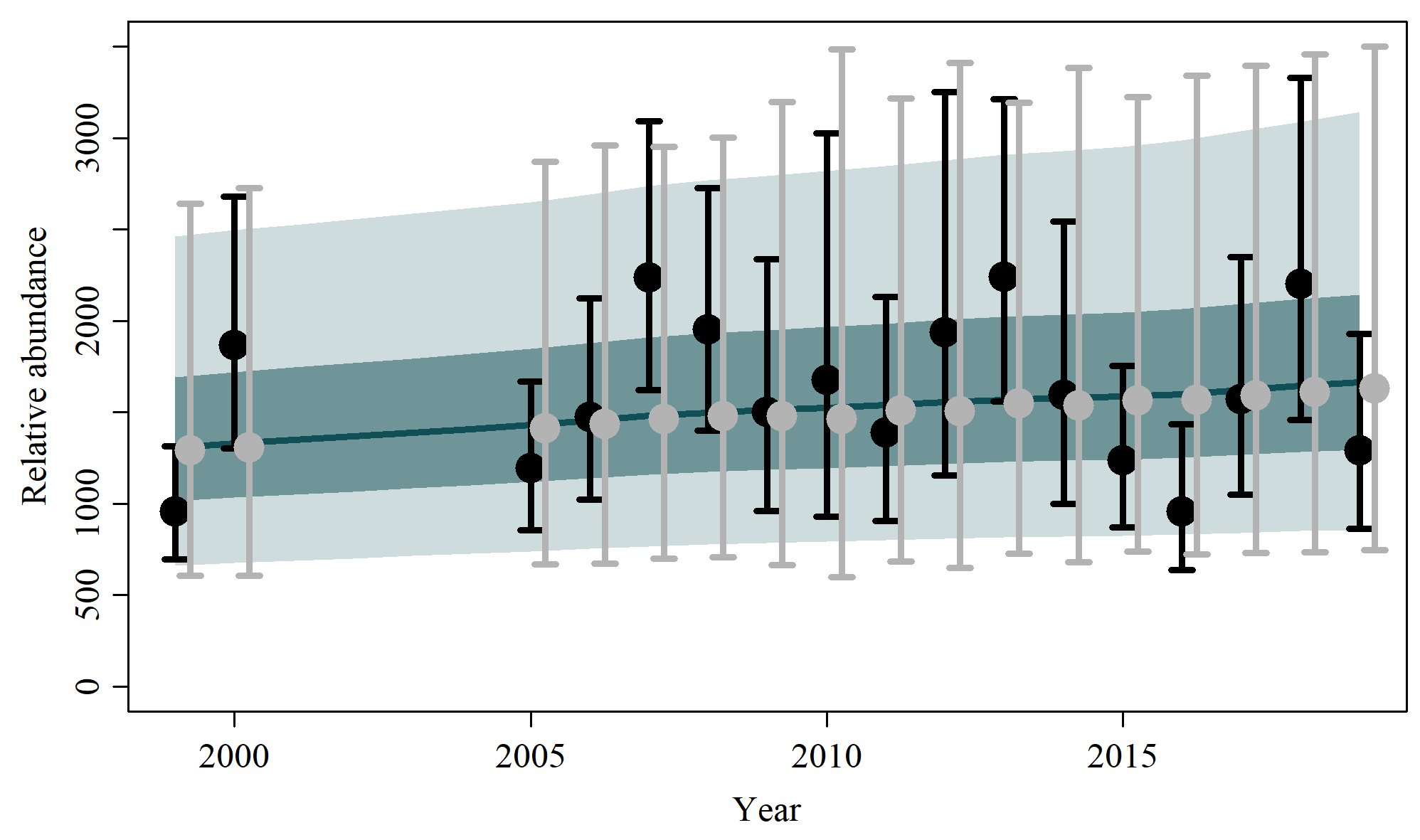


S1.3 Scen 2


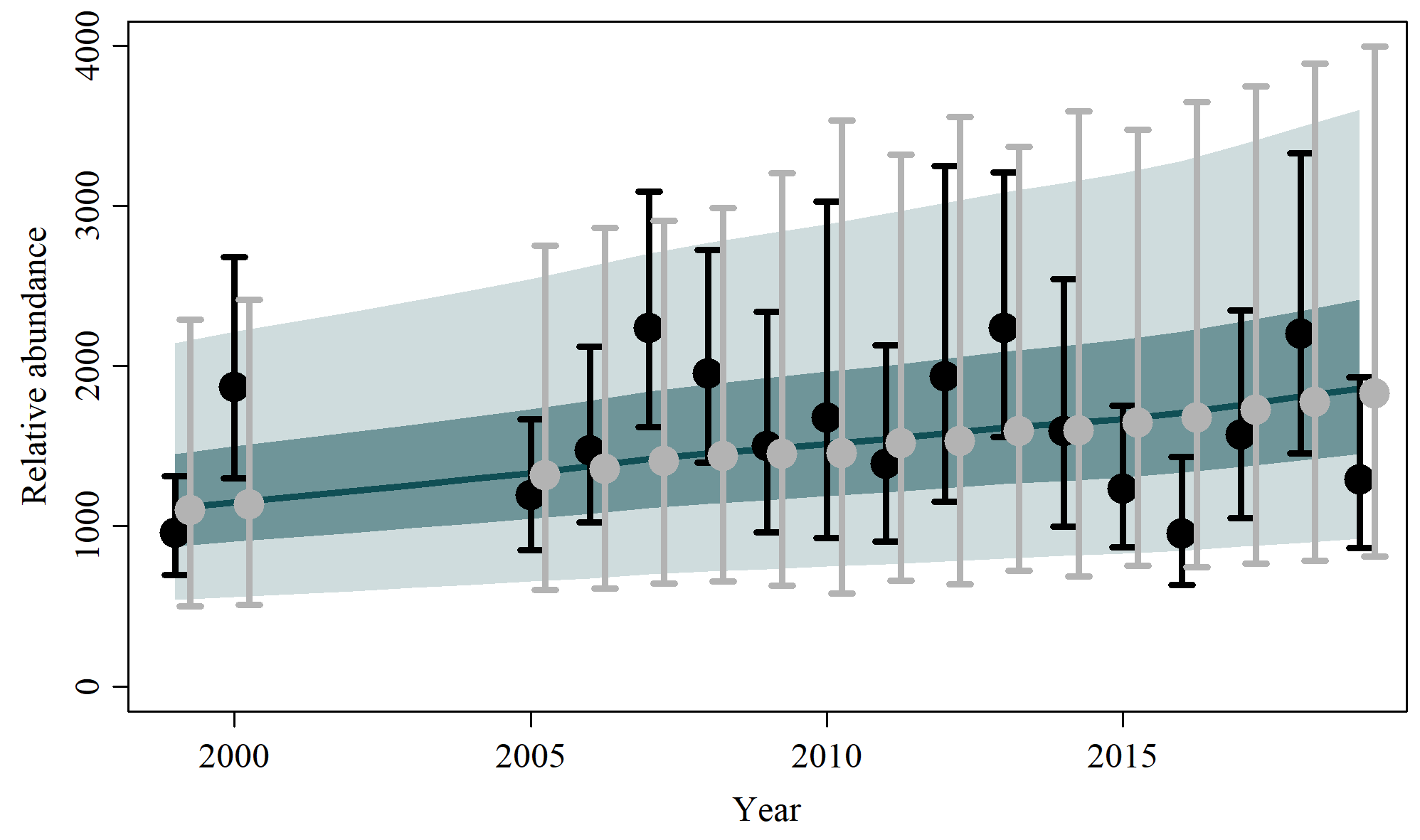


S1.4 Scen 3


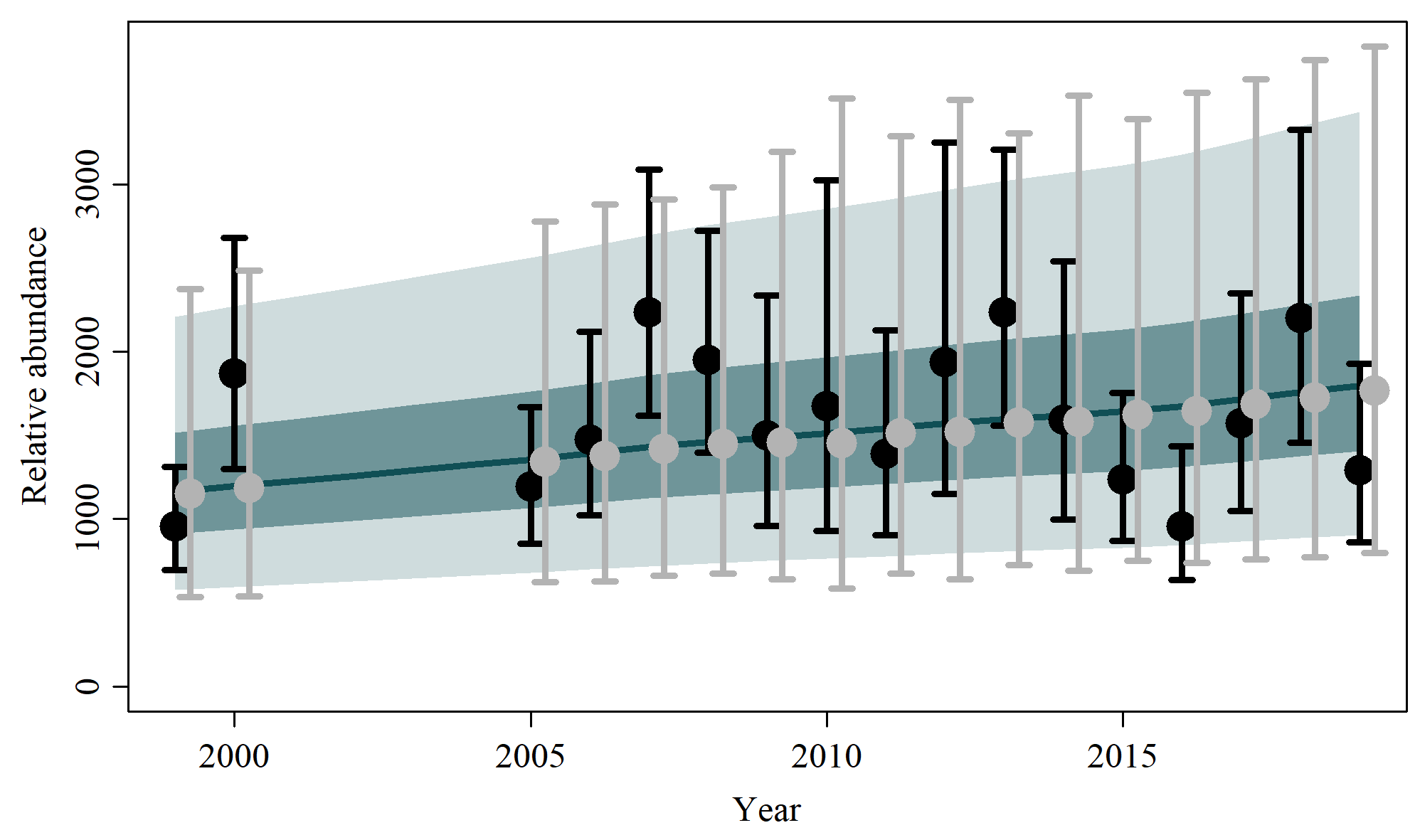


S1.5 Scen 4


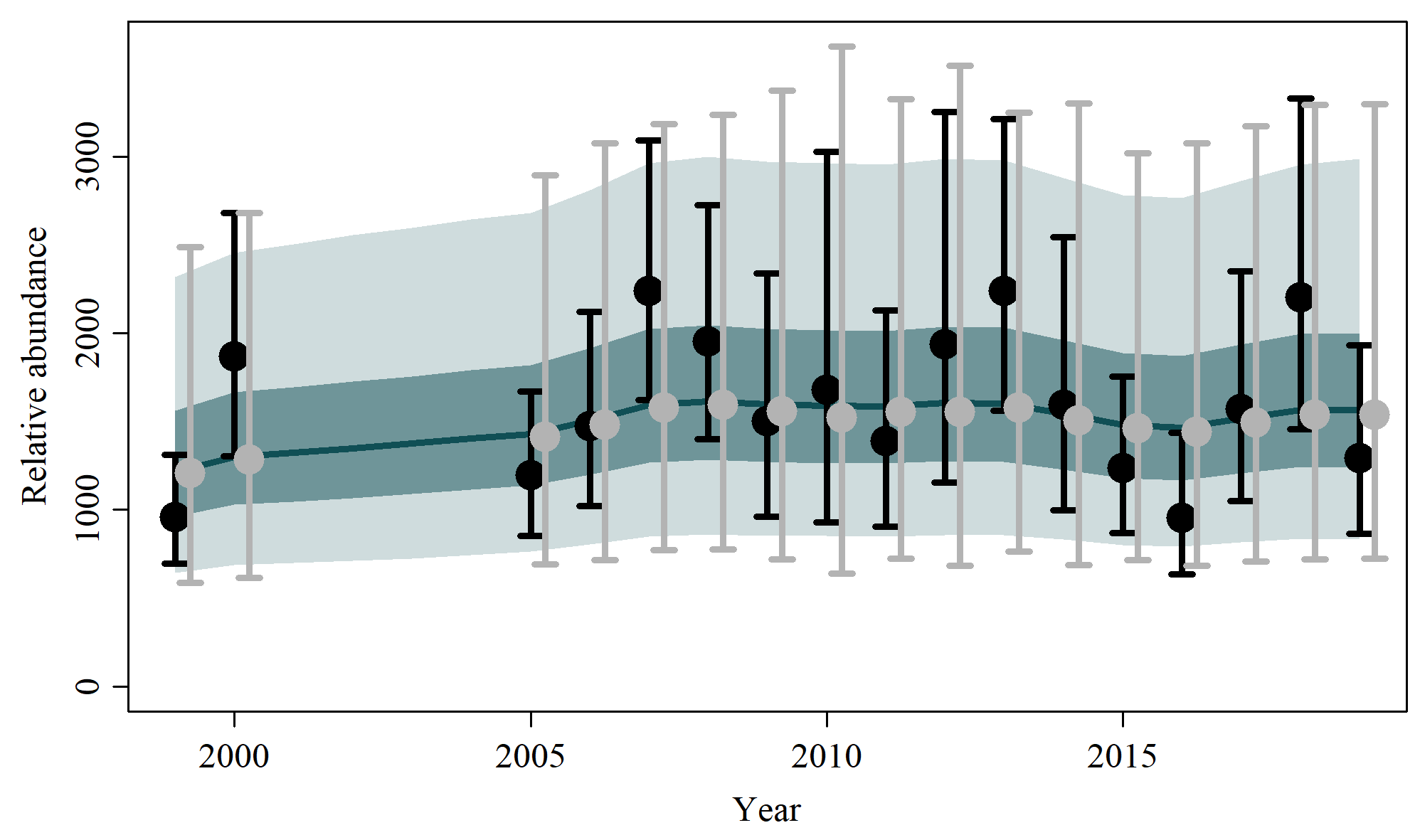


S1.6 Scen 5


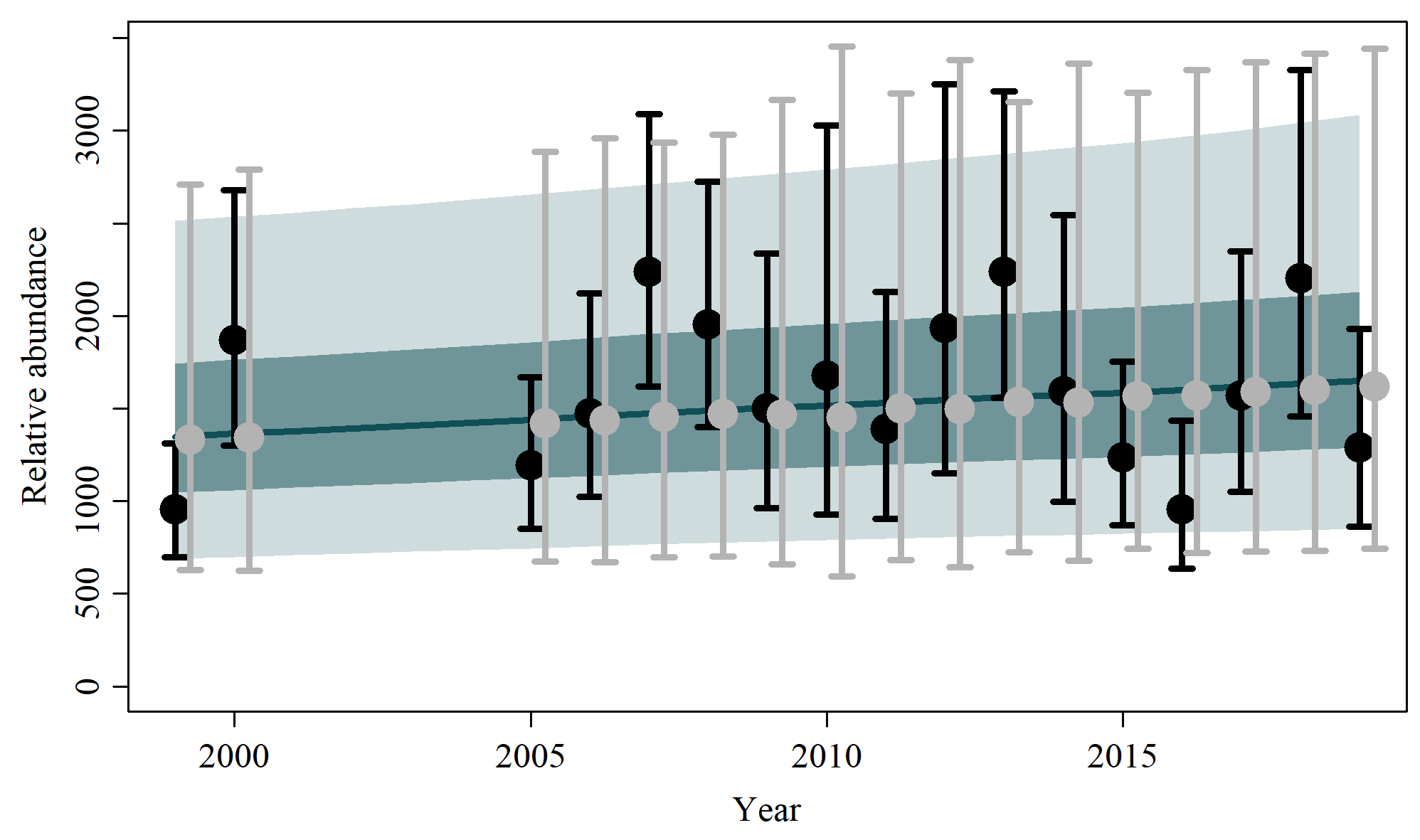


S1.7 Scen 6


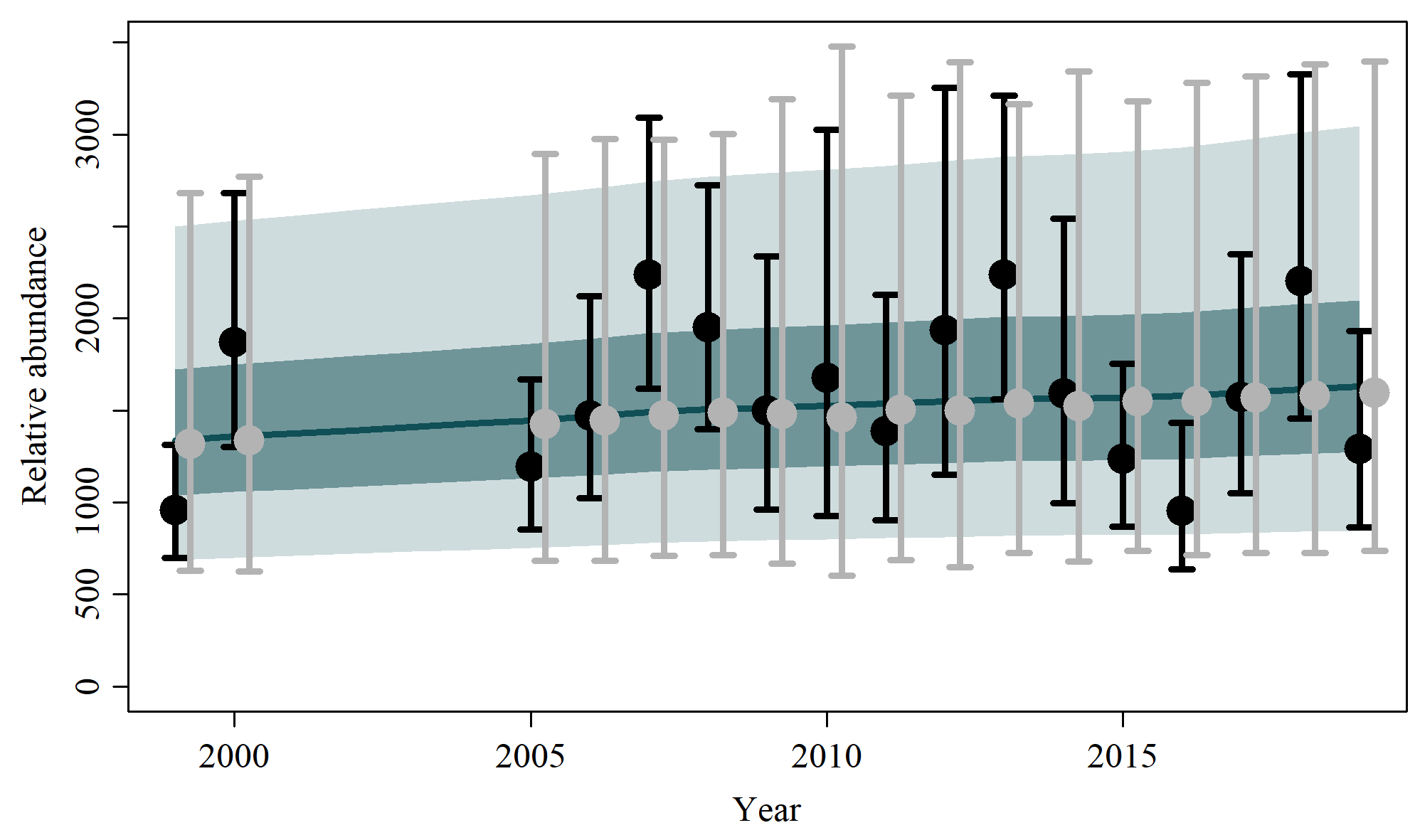


S1.8 Scen 7


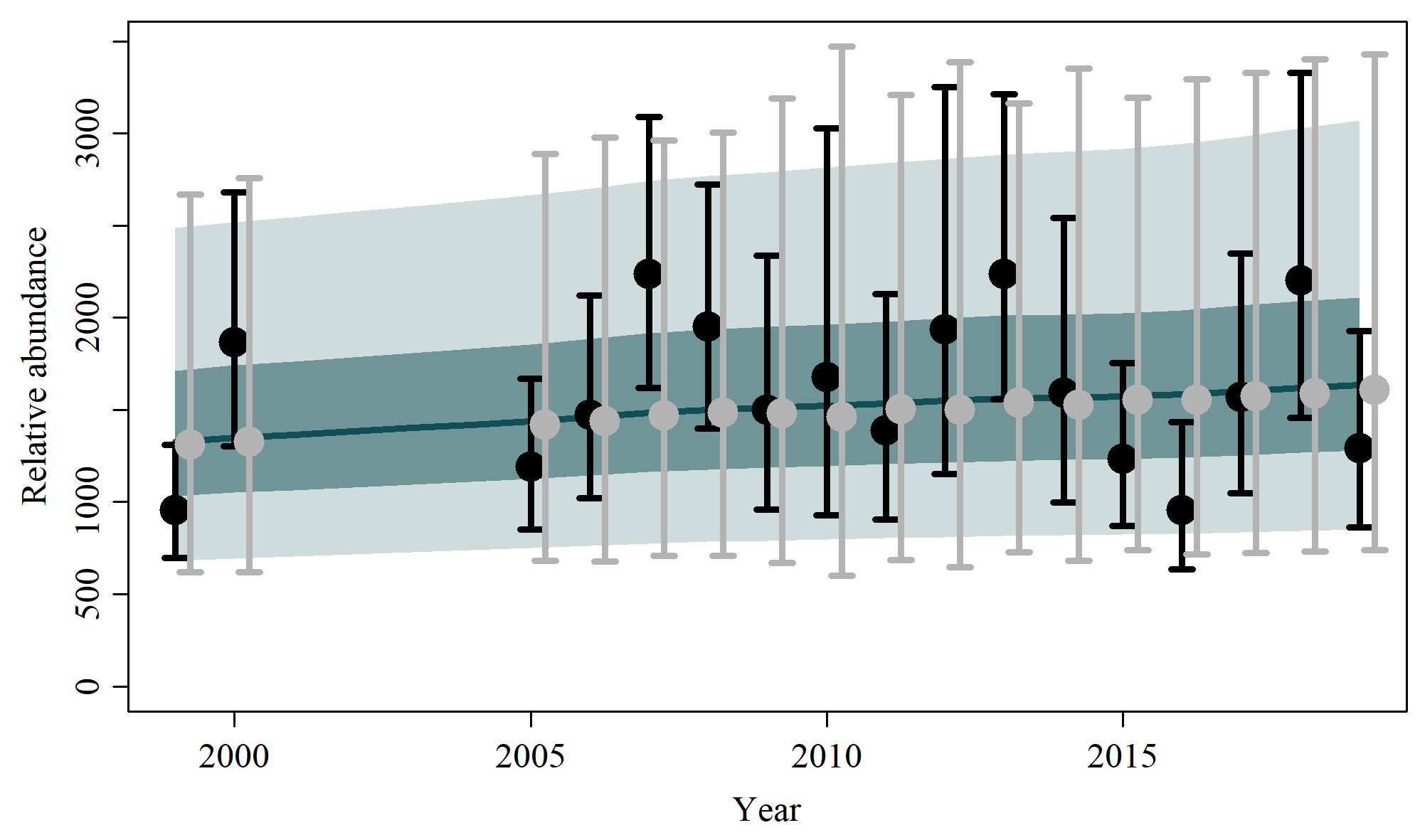


S1.9 Scen 8


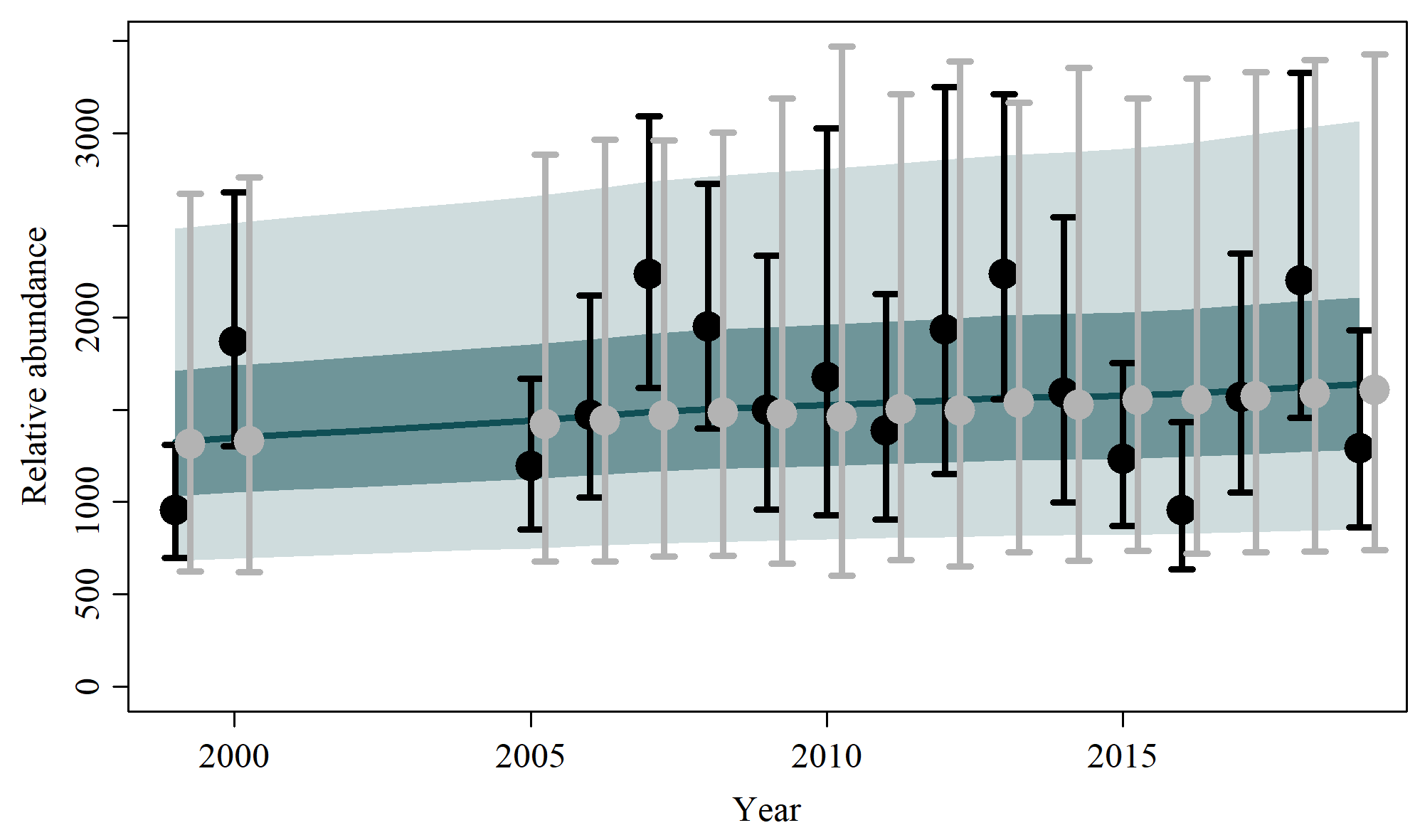


S1.10 Scen 9


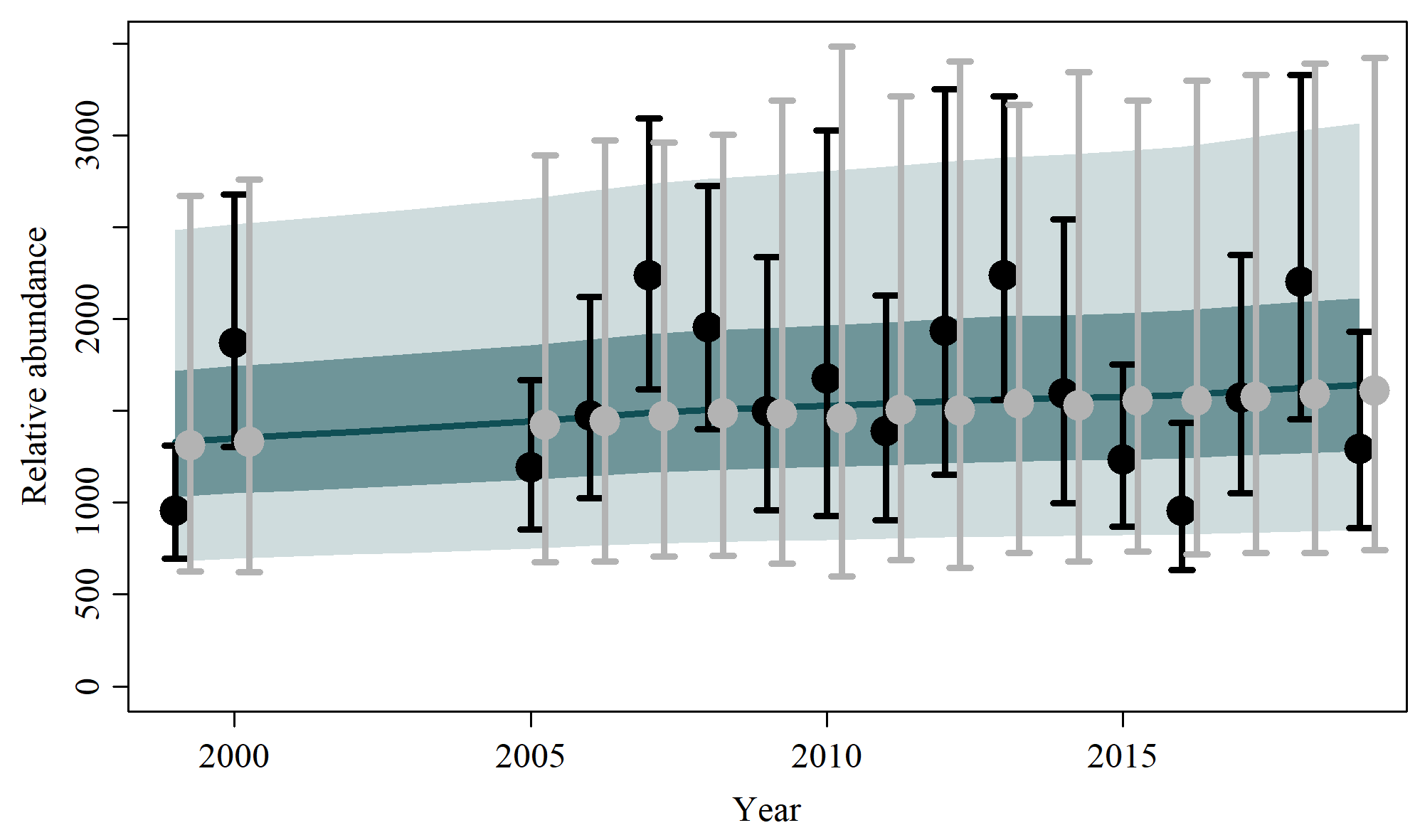


S1.11 Scen 10


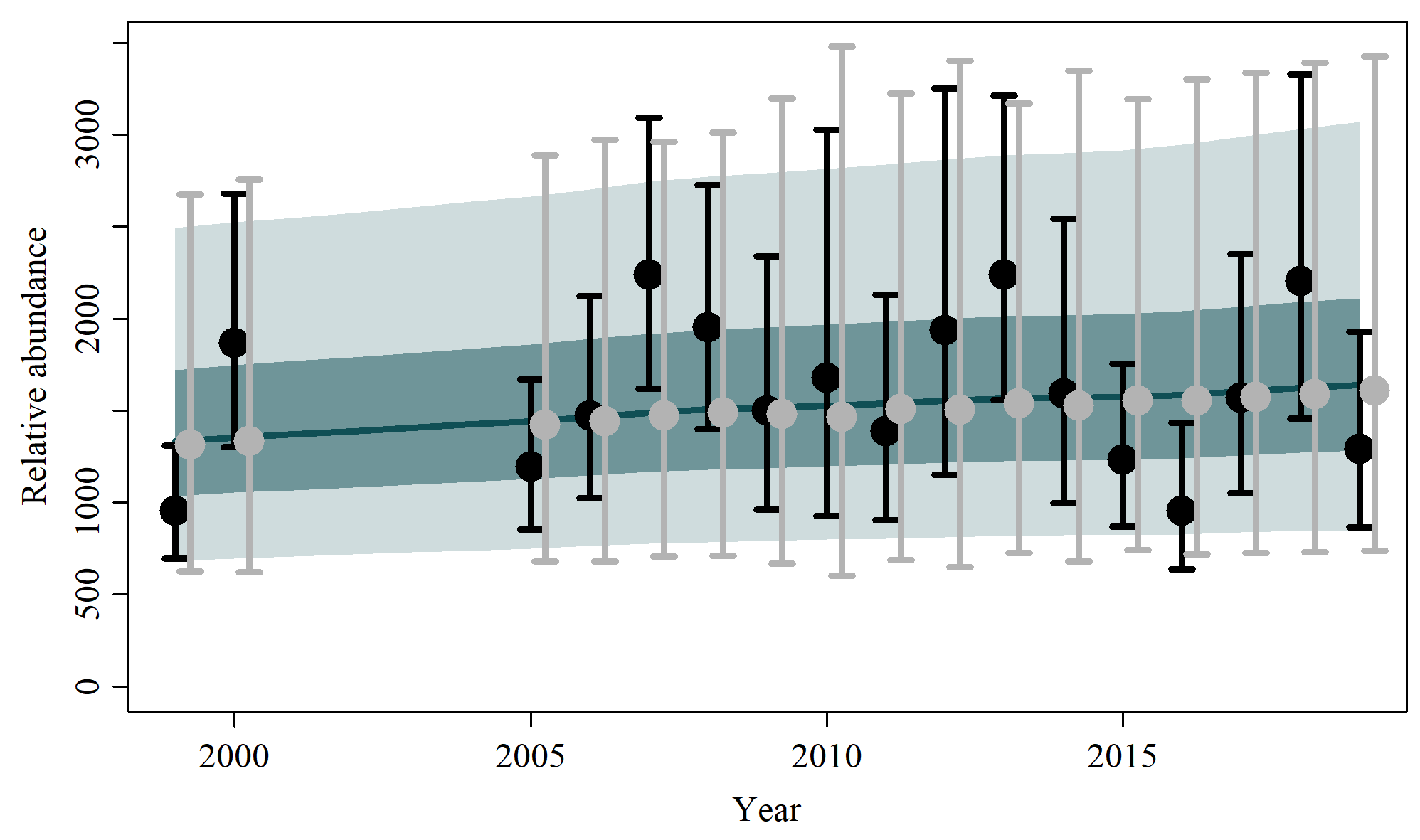


S1.12 Scen 11


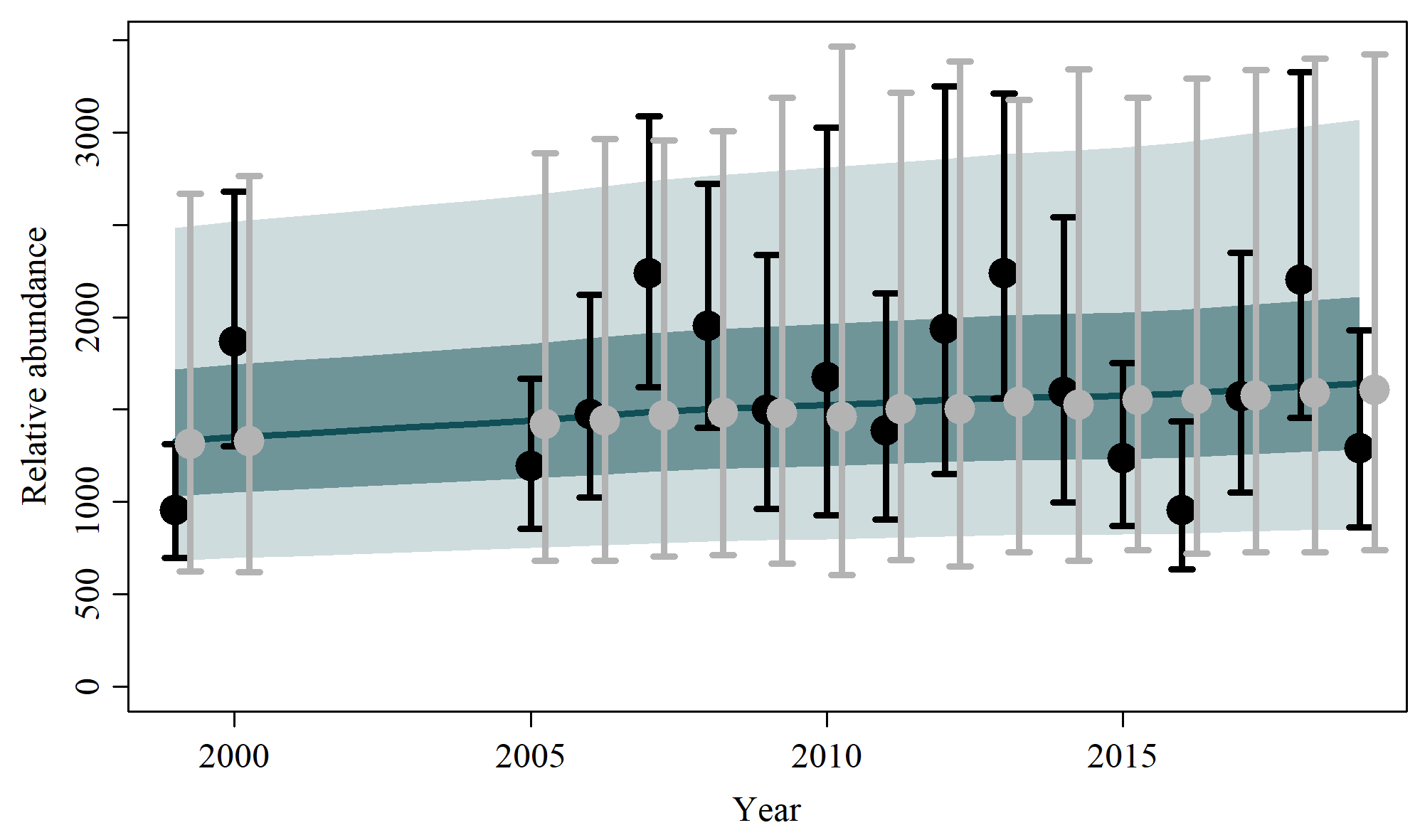


S1.13 Scen 12


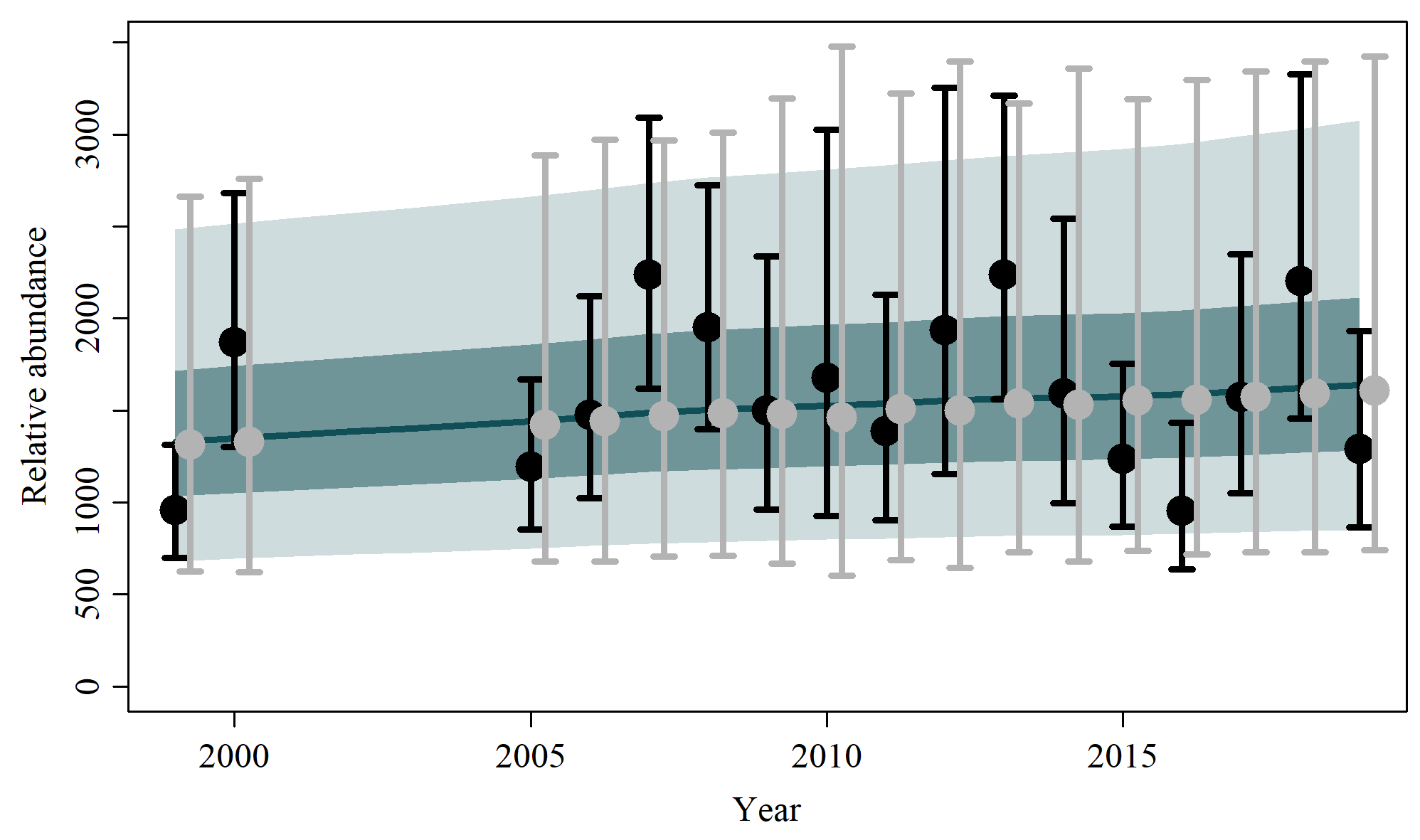


S1.14 Scen 13


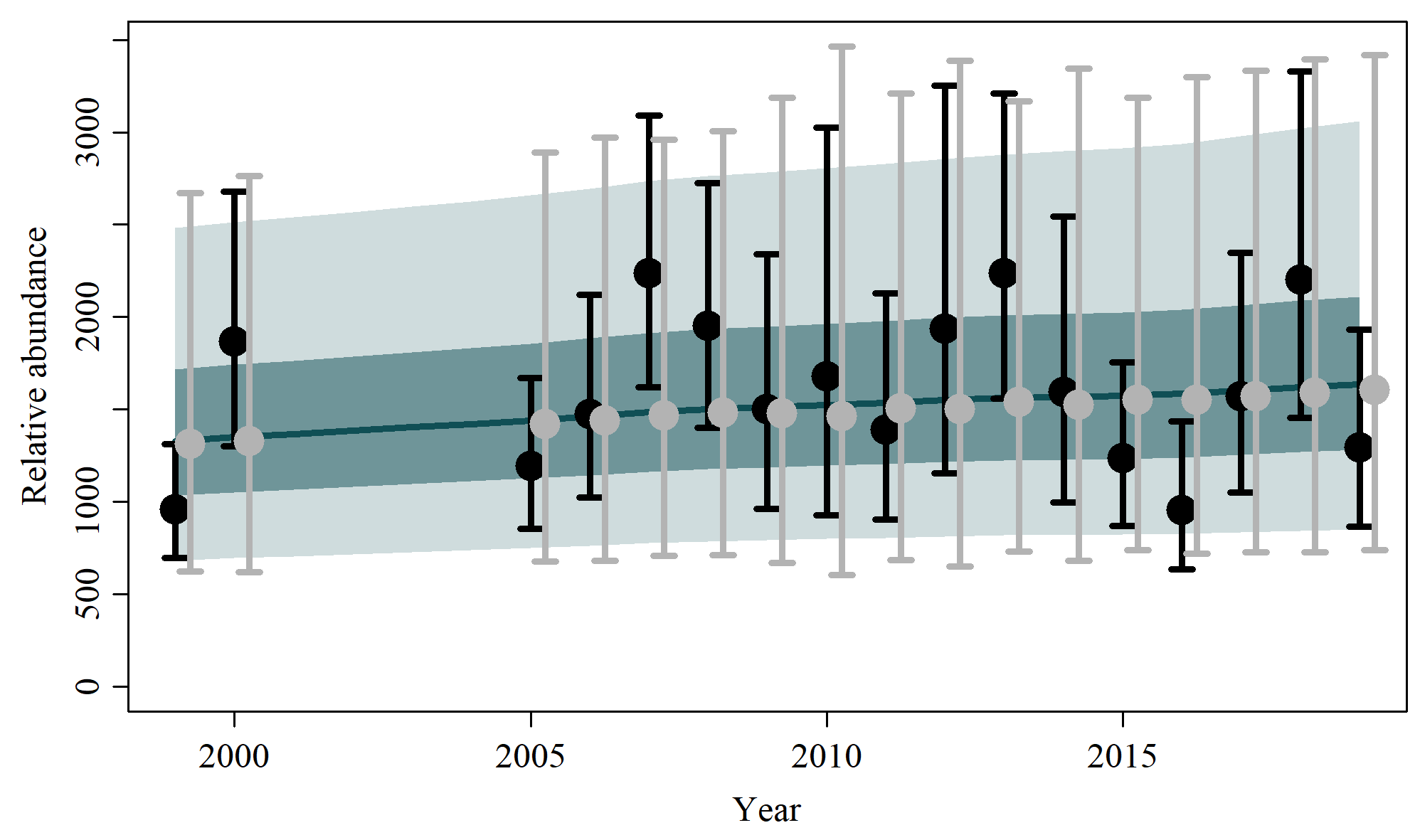


S1.15 Scen 14


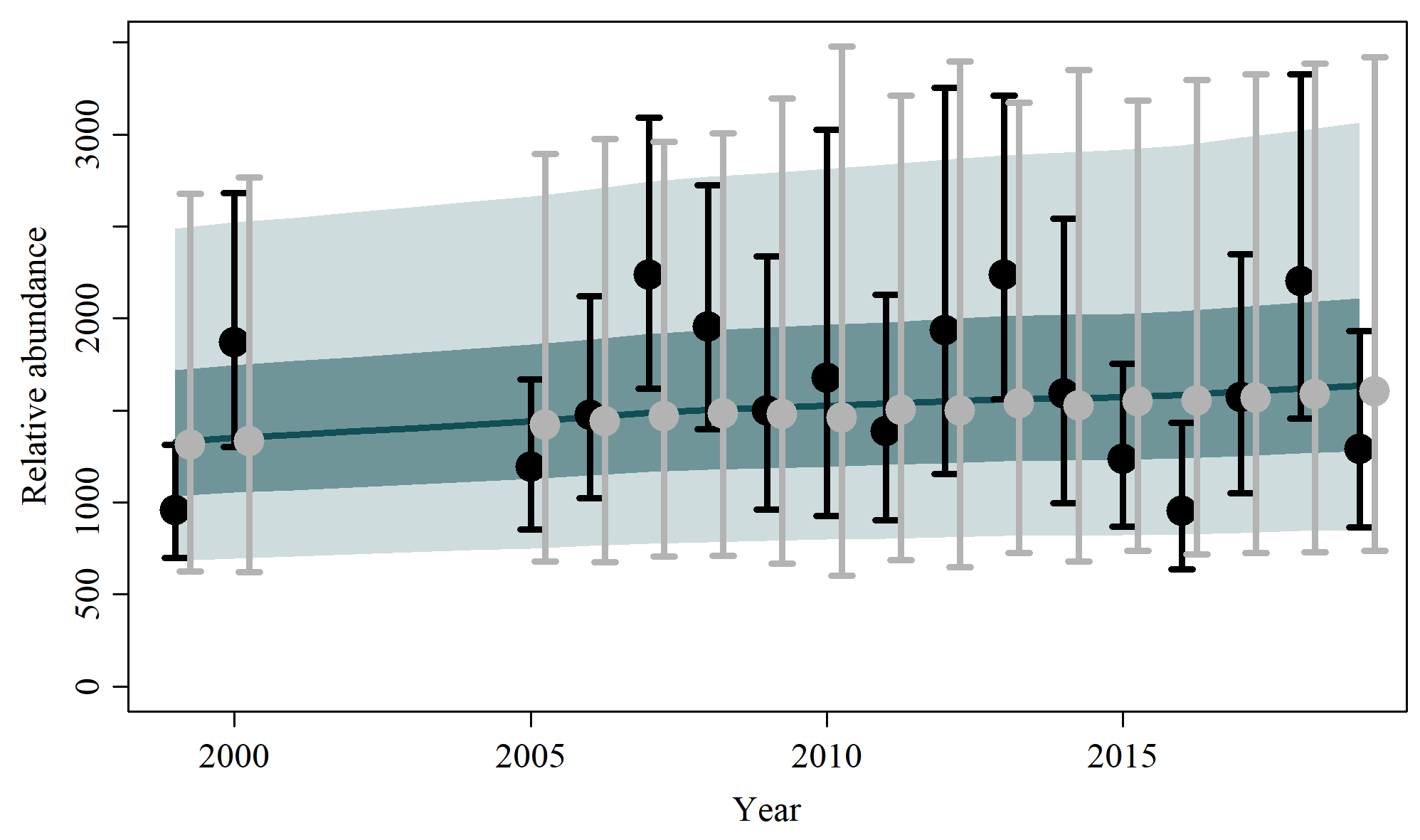


Figure S2. Posterior probability (thick line) and post-model pre-data (thin line) distributions of the key biological parameters for the base case and sensitivity scenarios of the assessment of southern right whale (SRW) *Eubalaena australis*. The posterior probability distribution of the base case is illustrated in the dashed grey line.

S2.1 Base Case


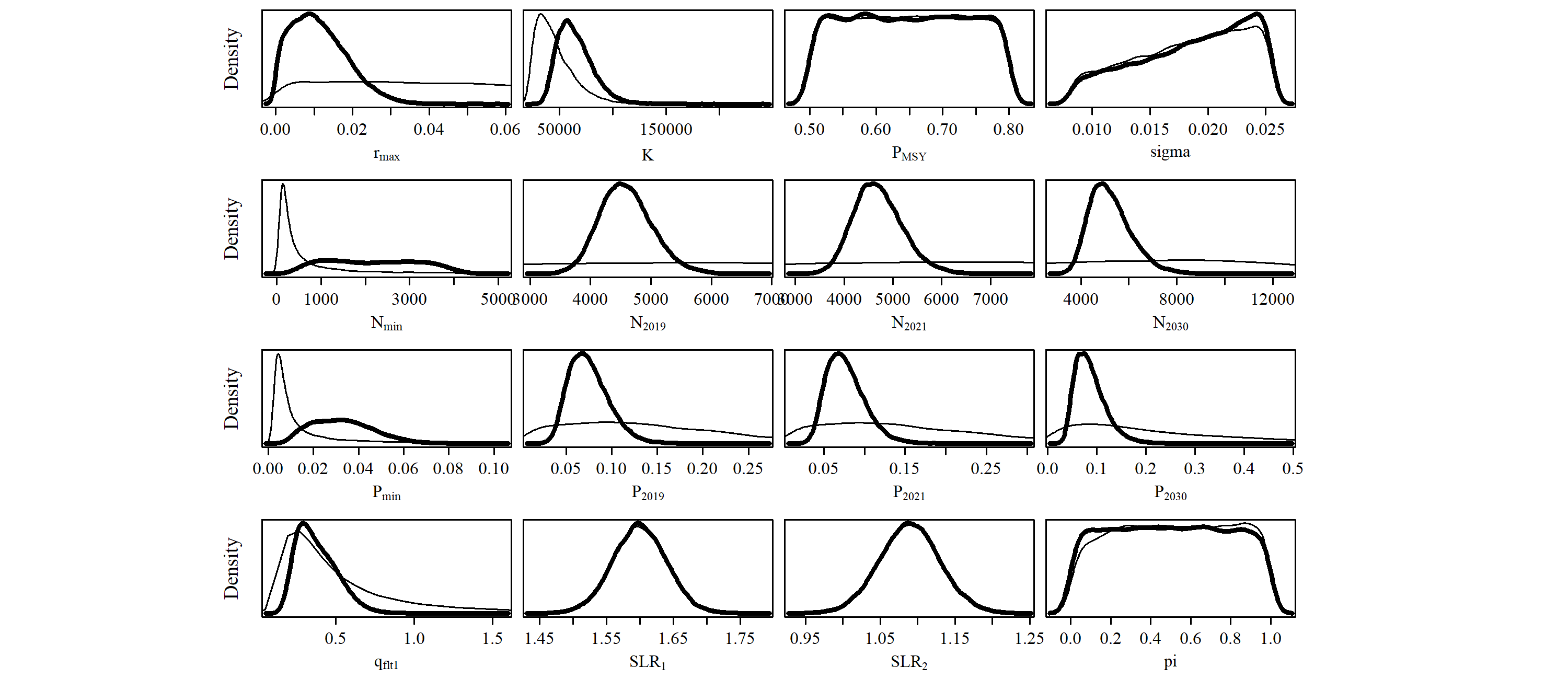


S2.2 Scen 1


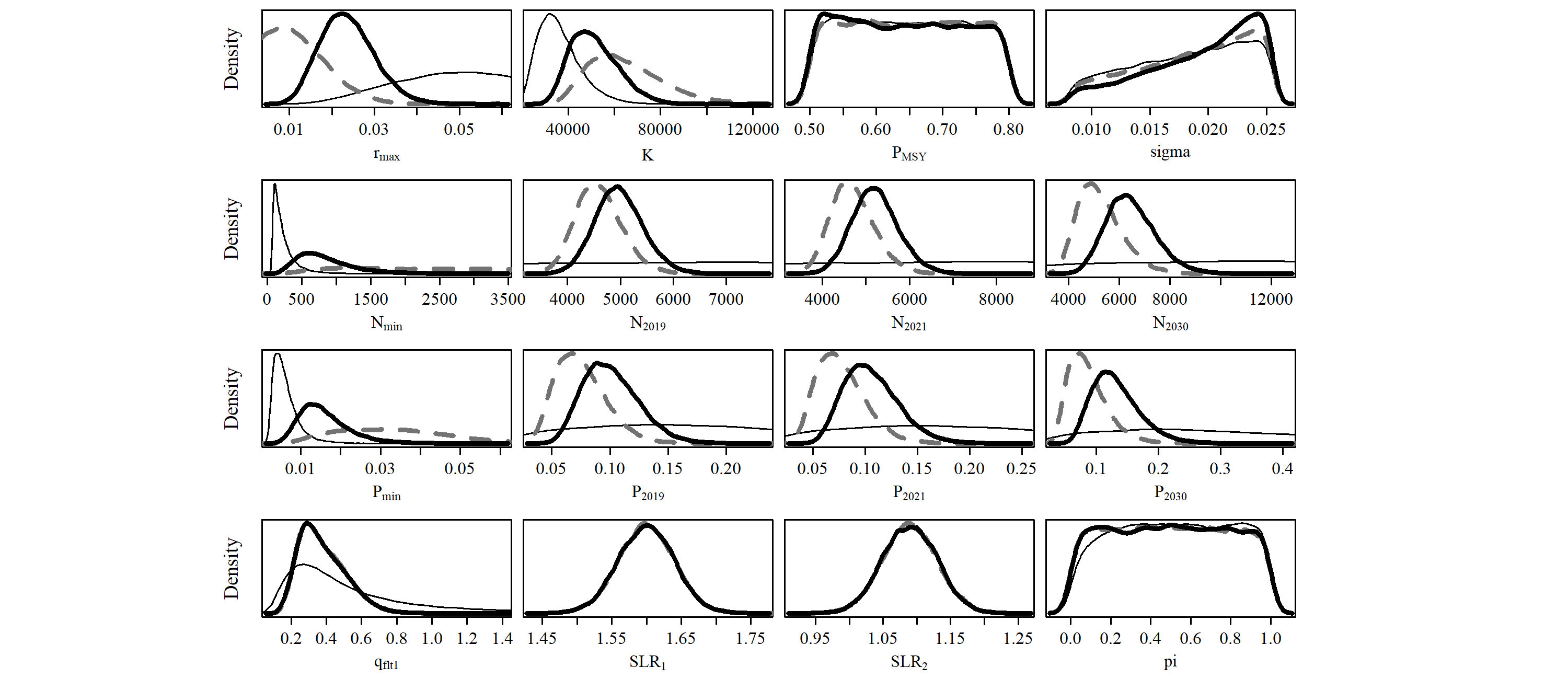


S2.3 Scen 2


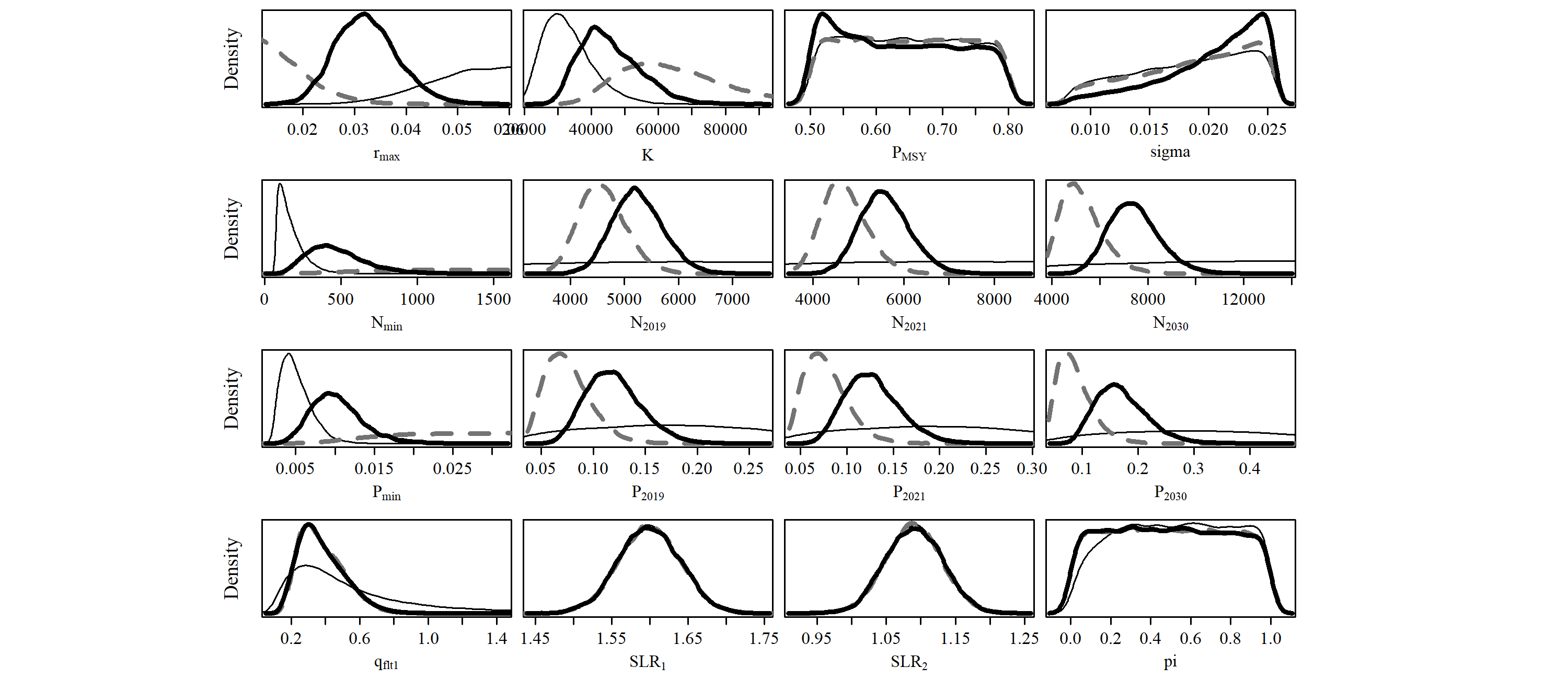


S2.4 Scen 3


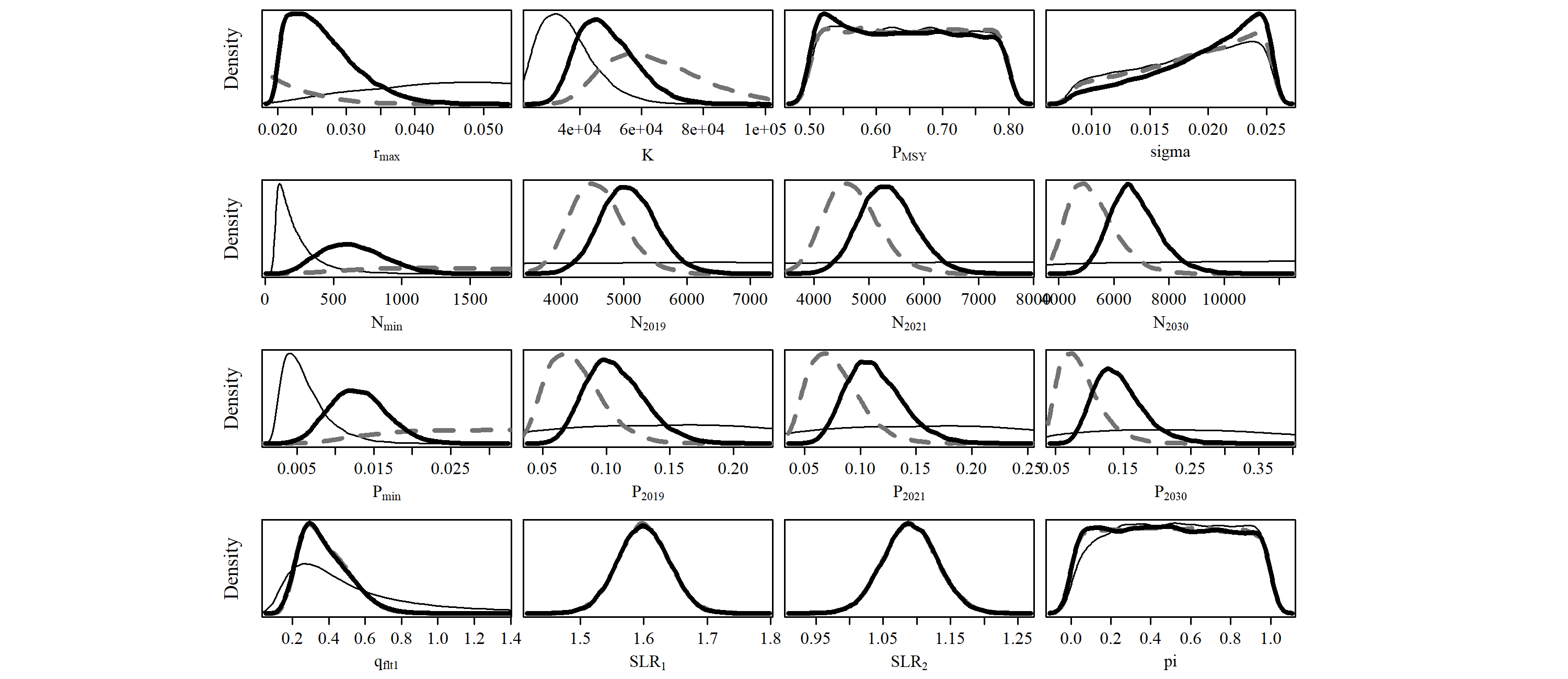


S2.5 Scen 4


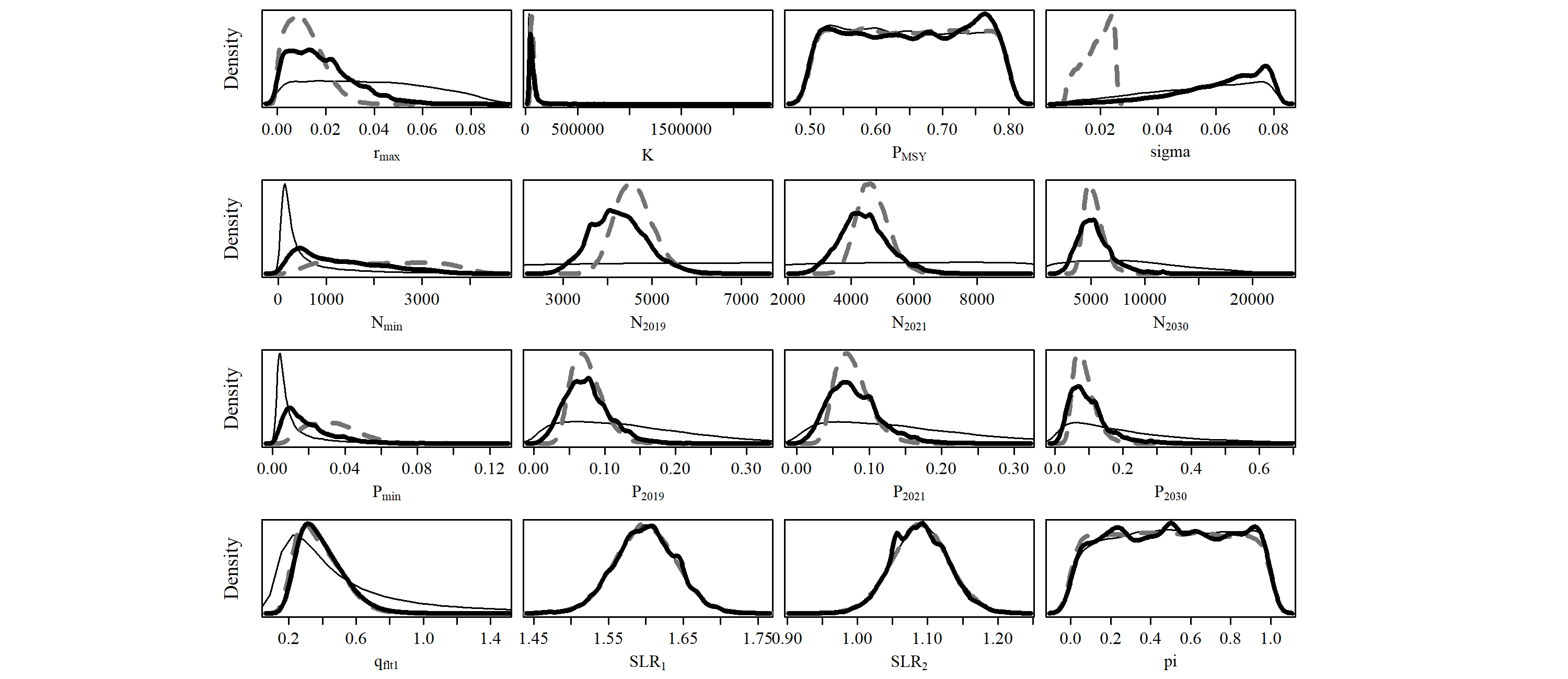


S2.6 Scen 5


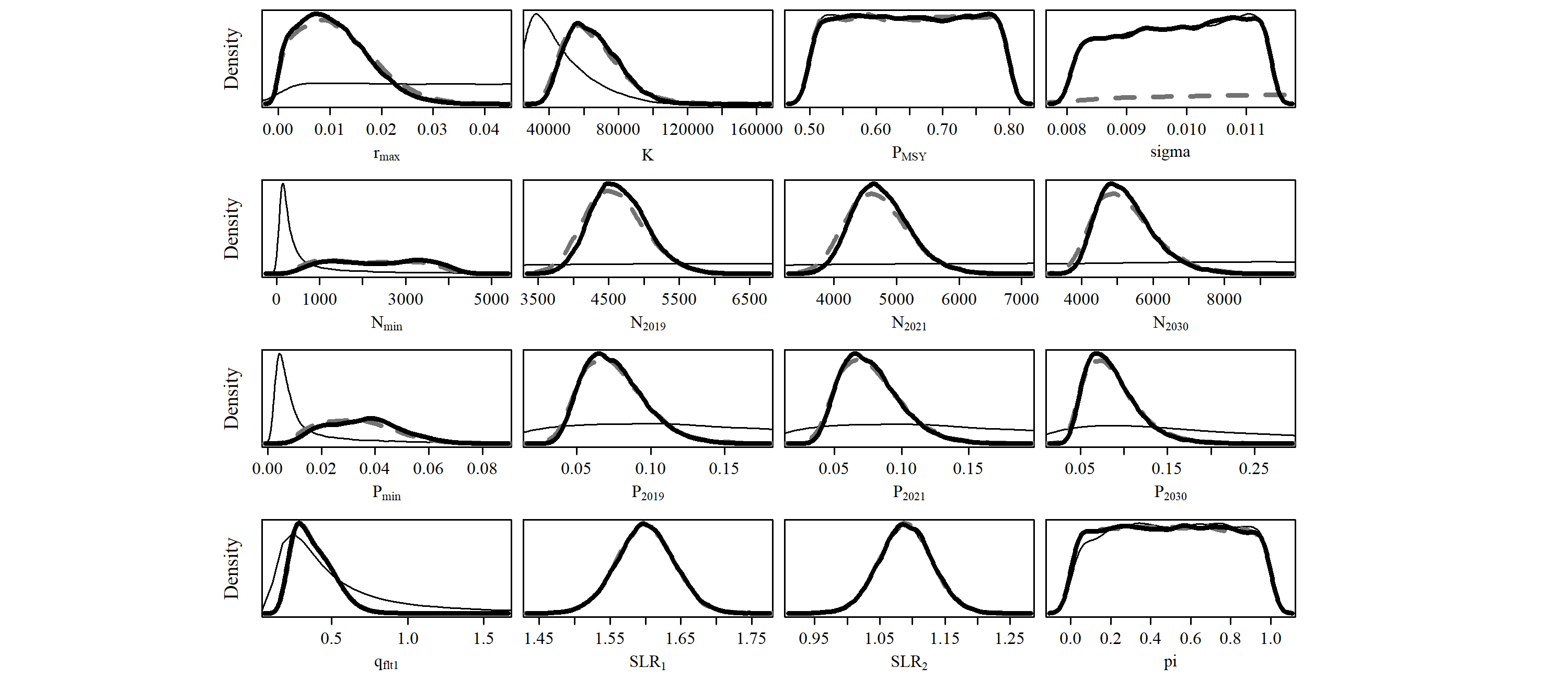


S2.7 Scen 6 (Note: target year is not included)


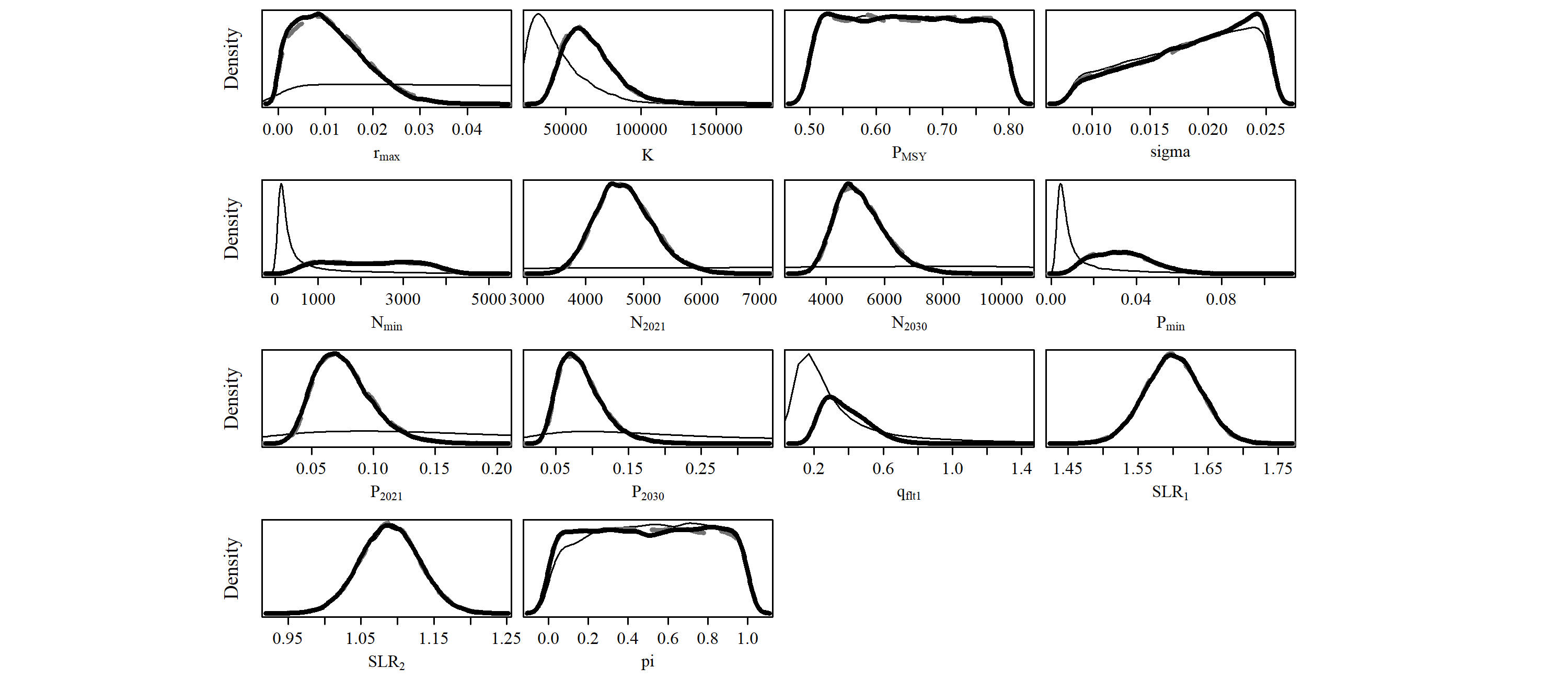


S2.8 Scen 7


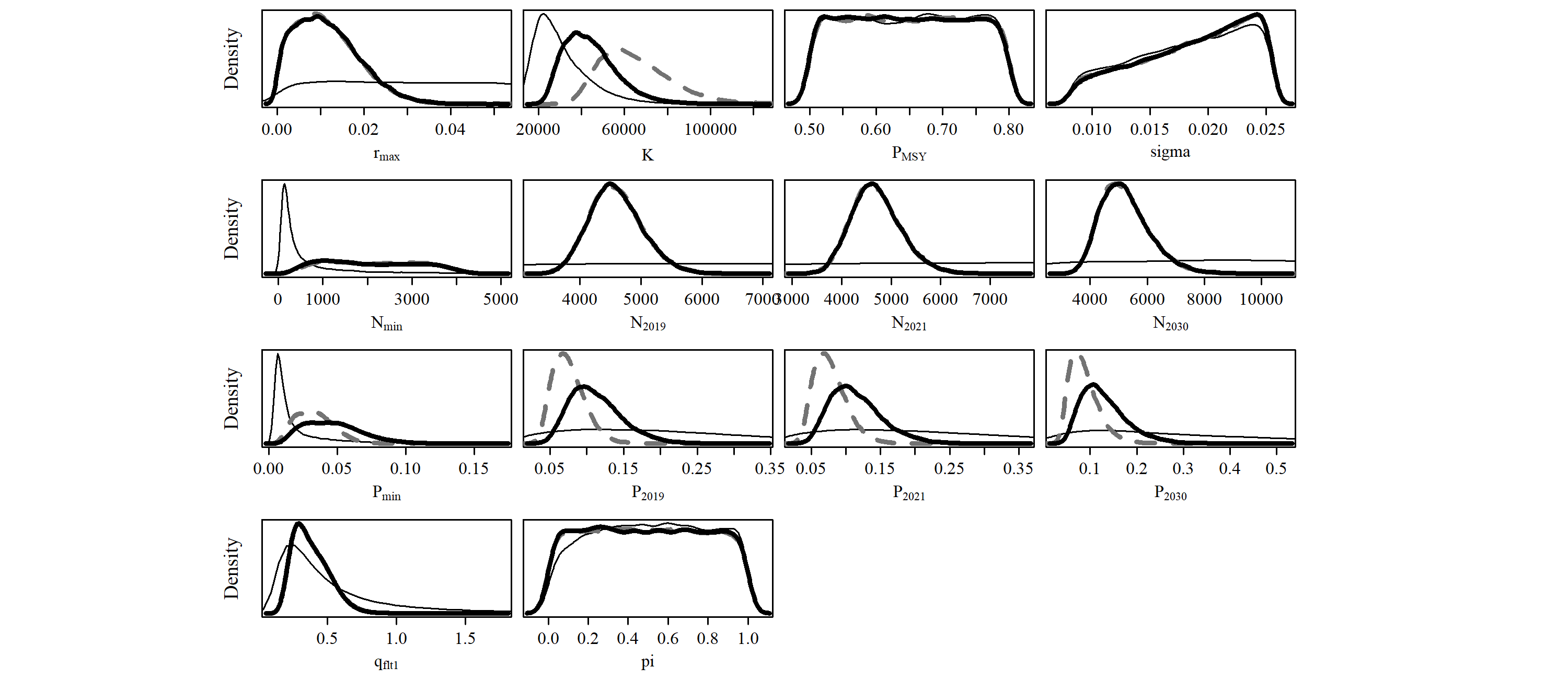


S2.9 Scen 8


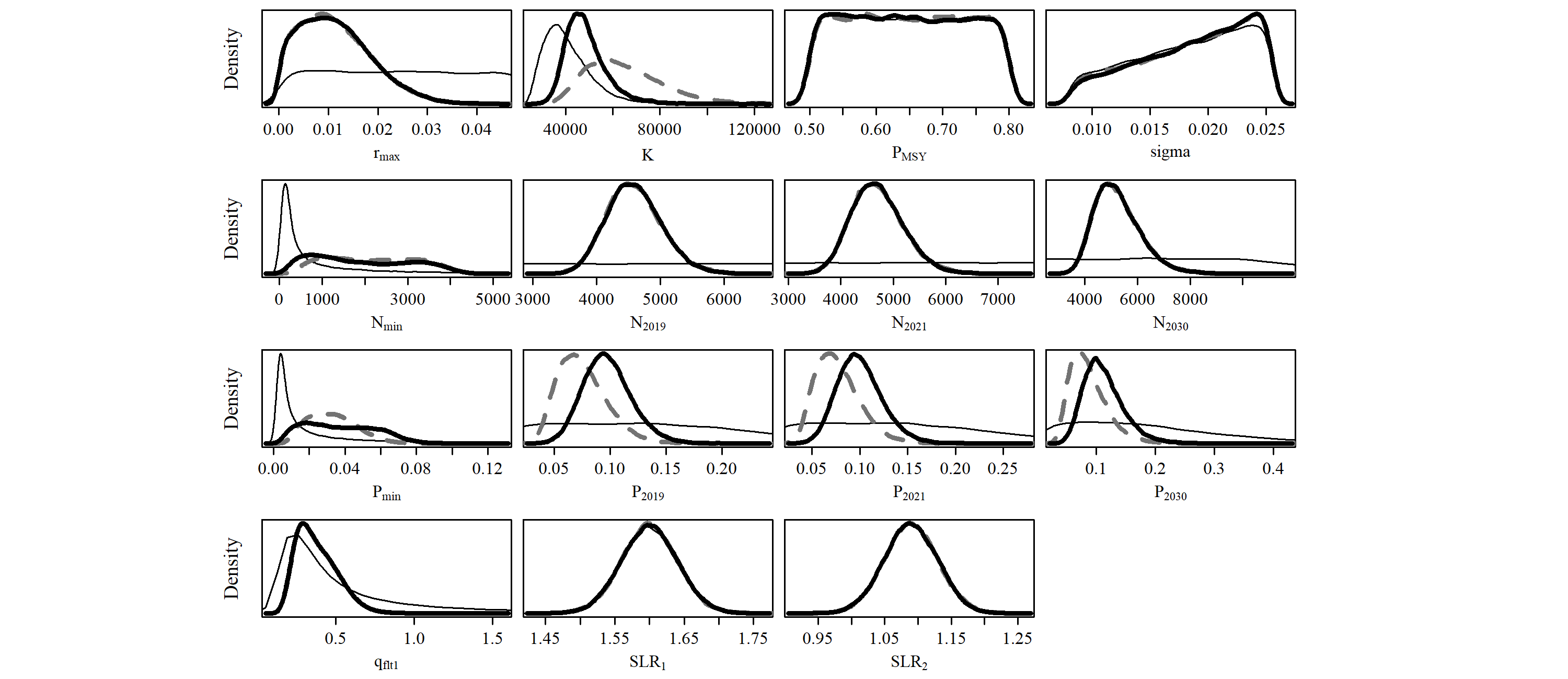


S2.10 Scen 9


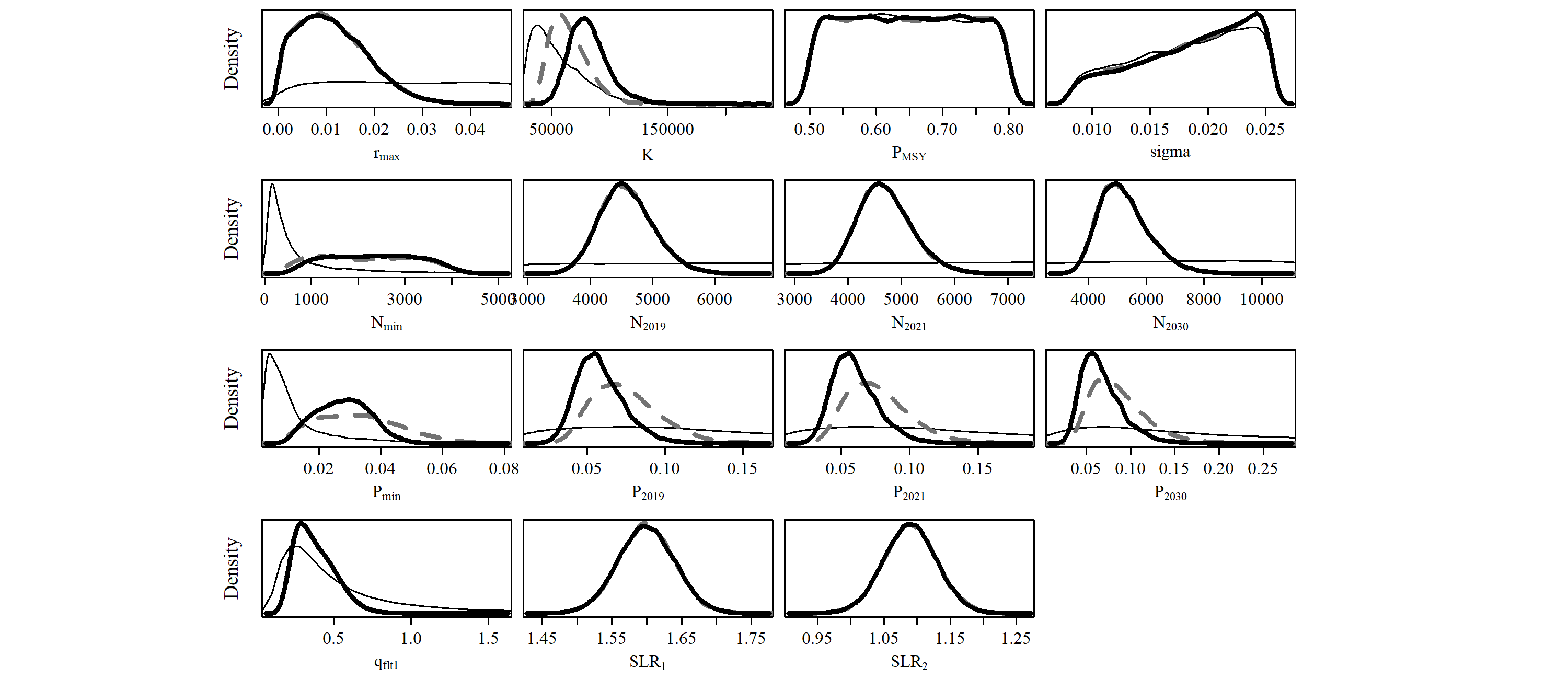


S2.11 Scen 10


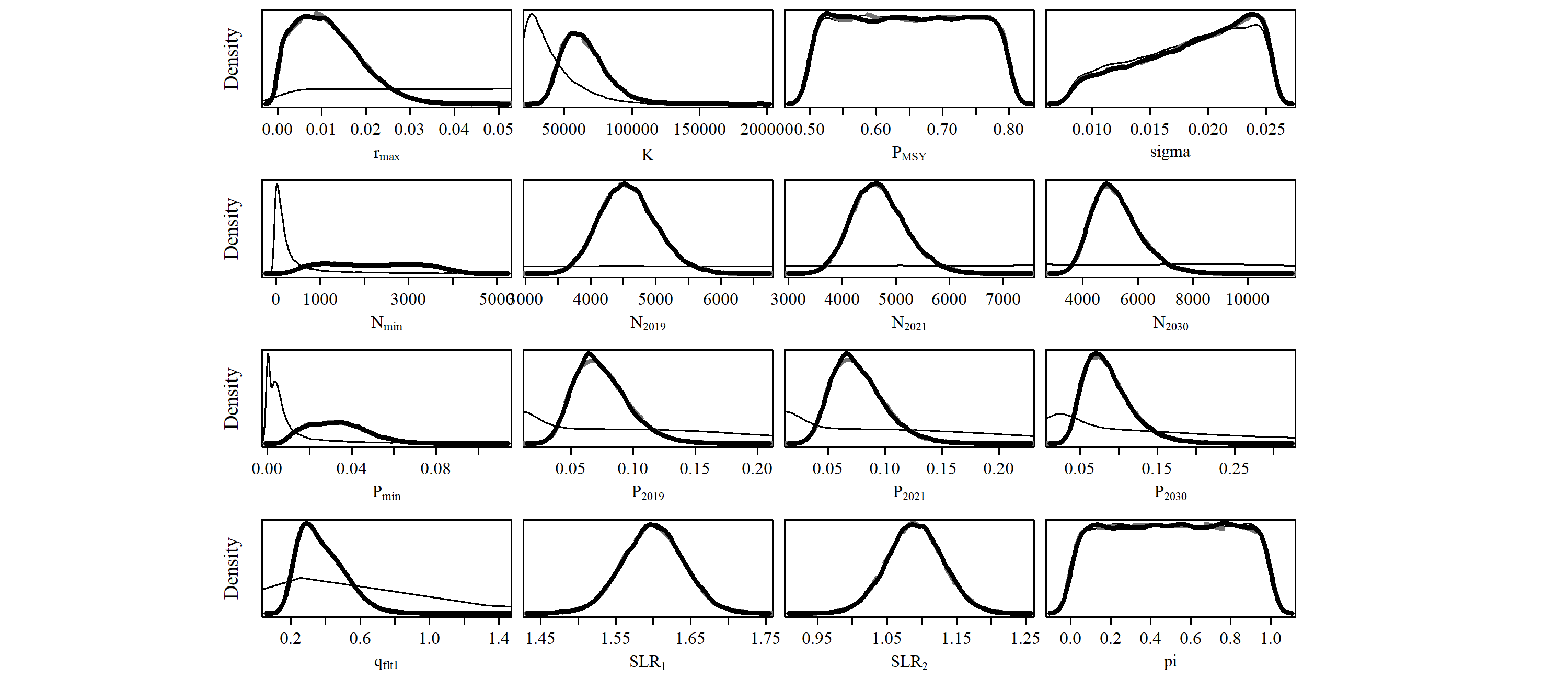


S2.12 Scen 11


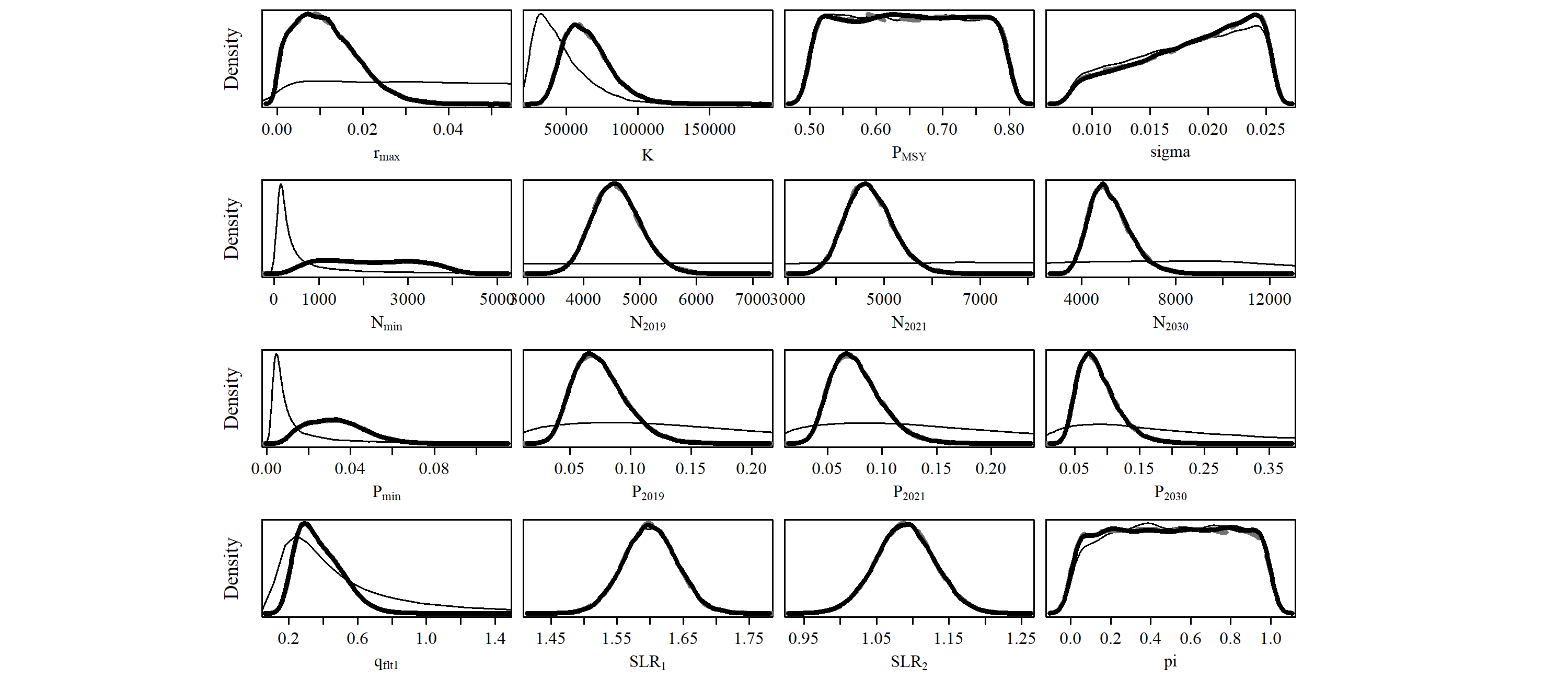


S2.13 Scen 12


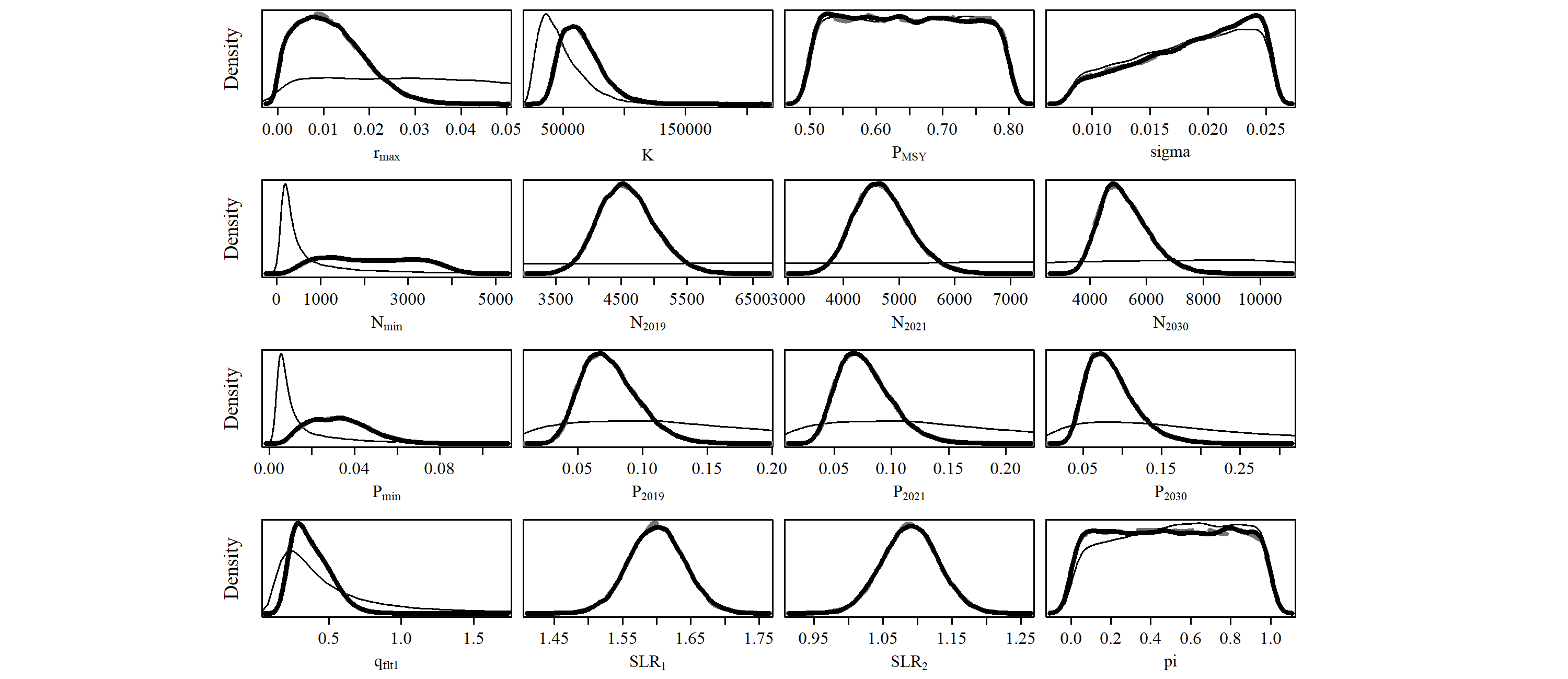


S2.14 Scen 13


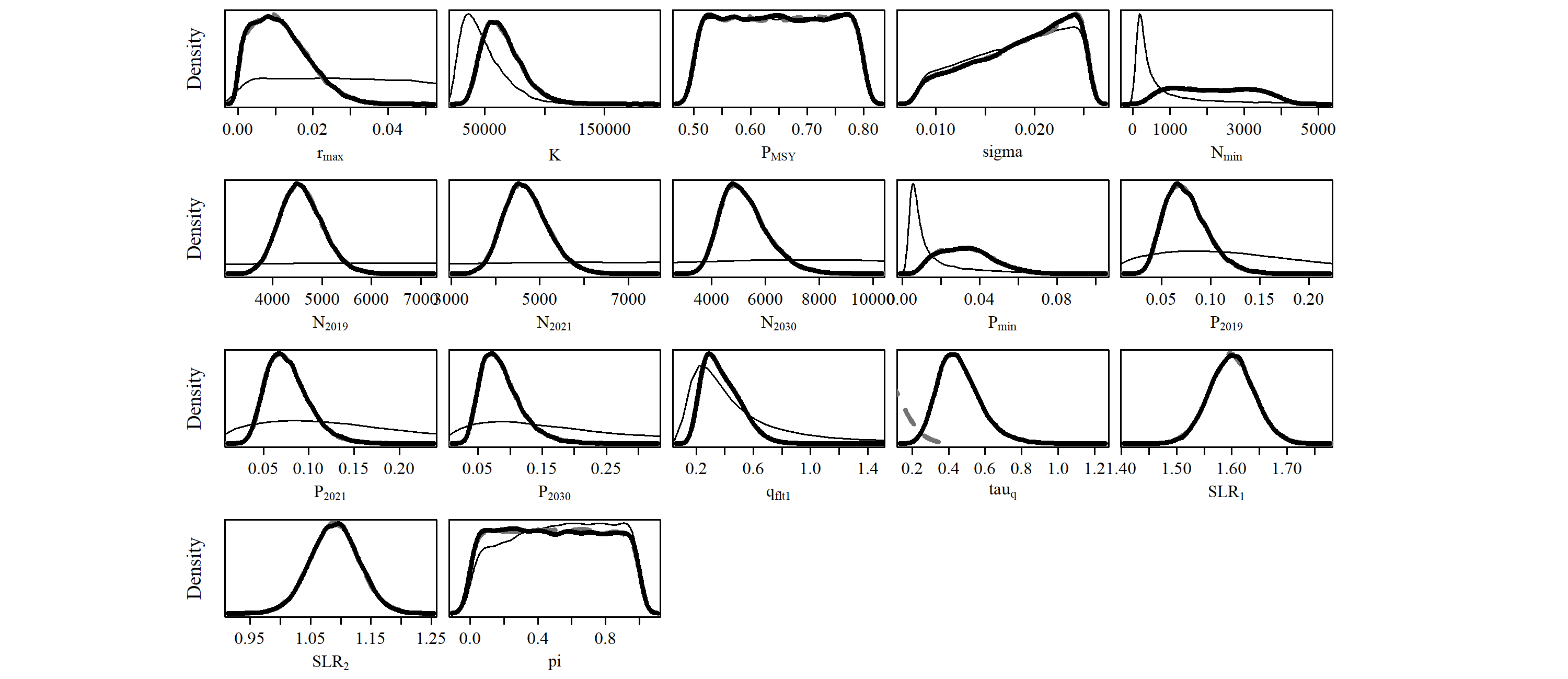


S2.15 Scen 14


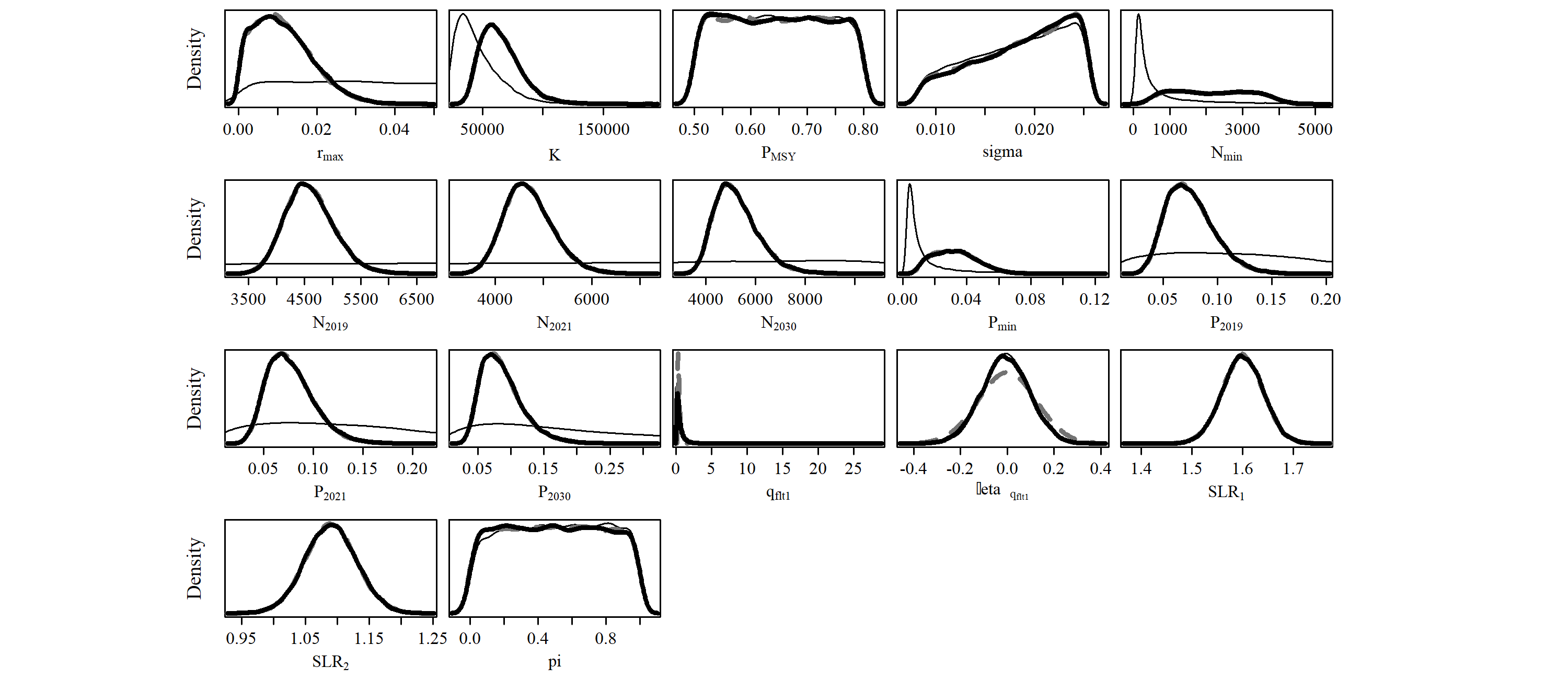


Figure S3. Population trajectories (blue lines) and time series of estimated catches (red lines) of southern right whale (SRW) *Eubalaena australis.* The solid blue line represents the median estimated model-averaged trajectory of the population abundance ($N_{y}$), while the shaded areas correspond to the 50% and 95% credible intervals. The dashed line represents the median estimated base case trajectory of the abundance. The solid red line represents the average number of whaling catches as estimated by the catch parameter ($\pi$), while the red shaded areas correspond to the 50% and 95% credible intervals. The grey and black dots represent the estimated and observed, respectively, absolute abundance in 2010.

S3.1 Base Case


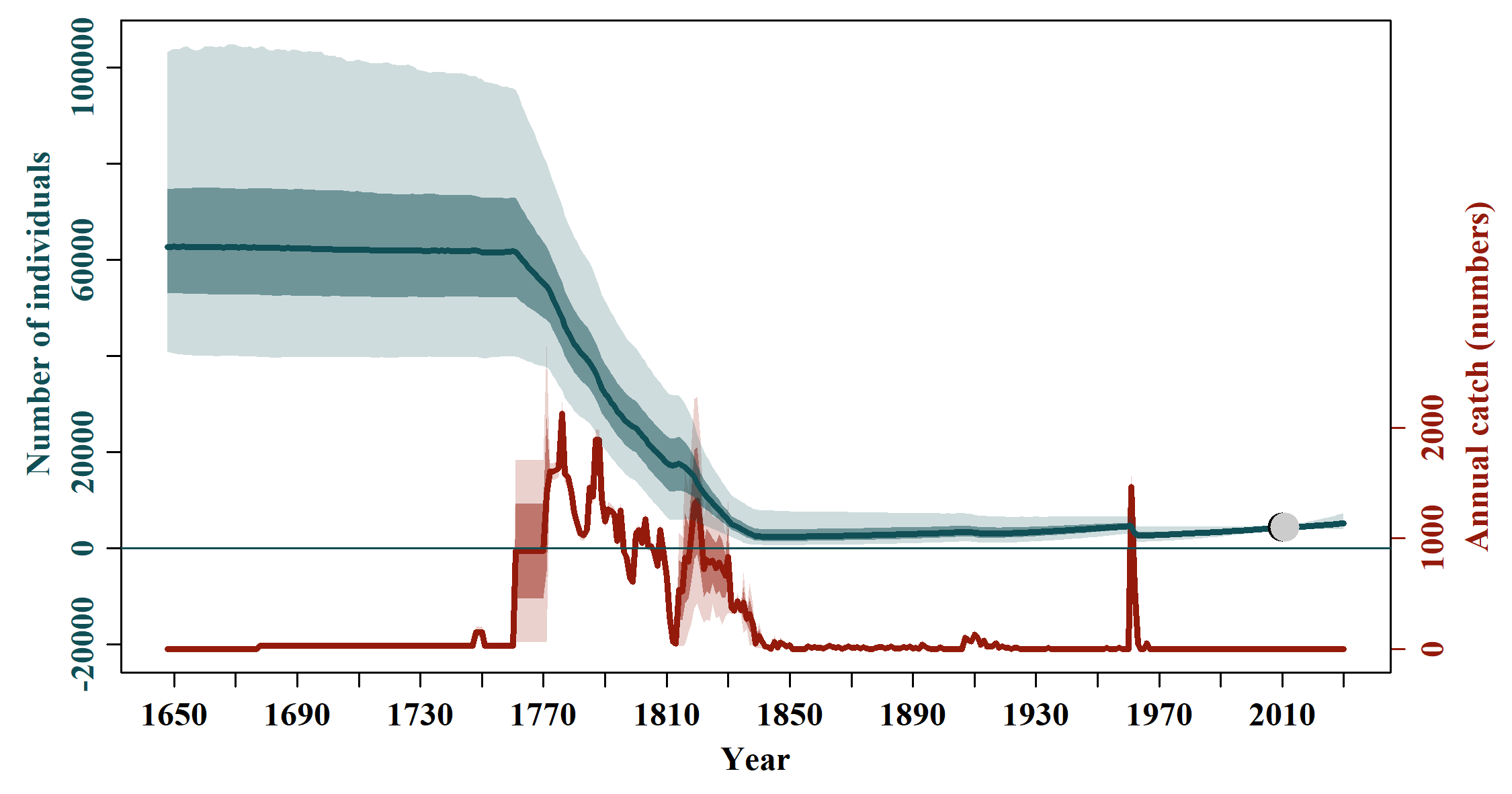


S3.2 Scen 1


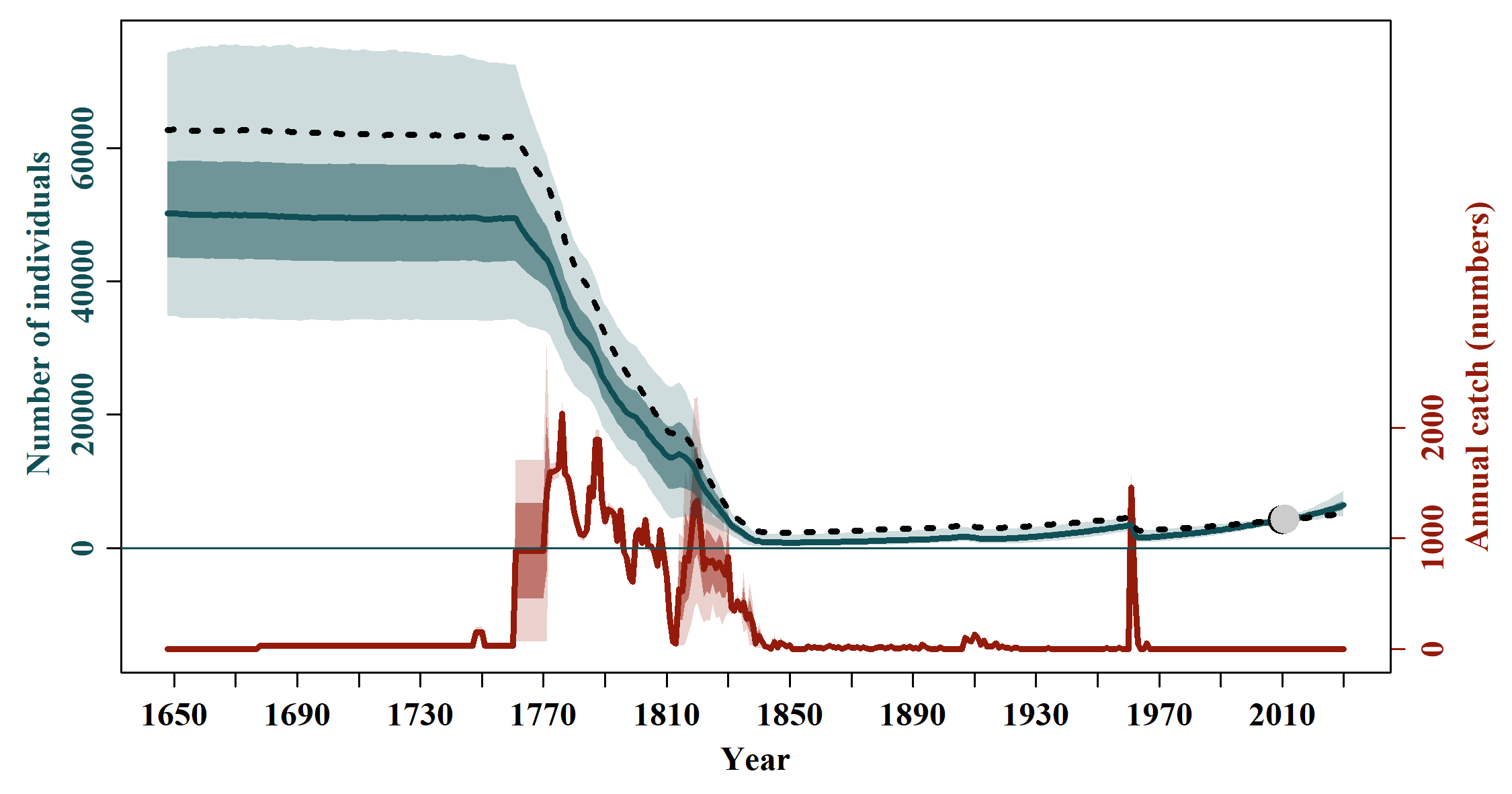


S3.3 Scen 2


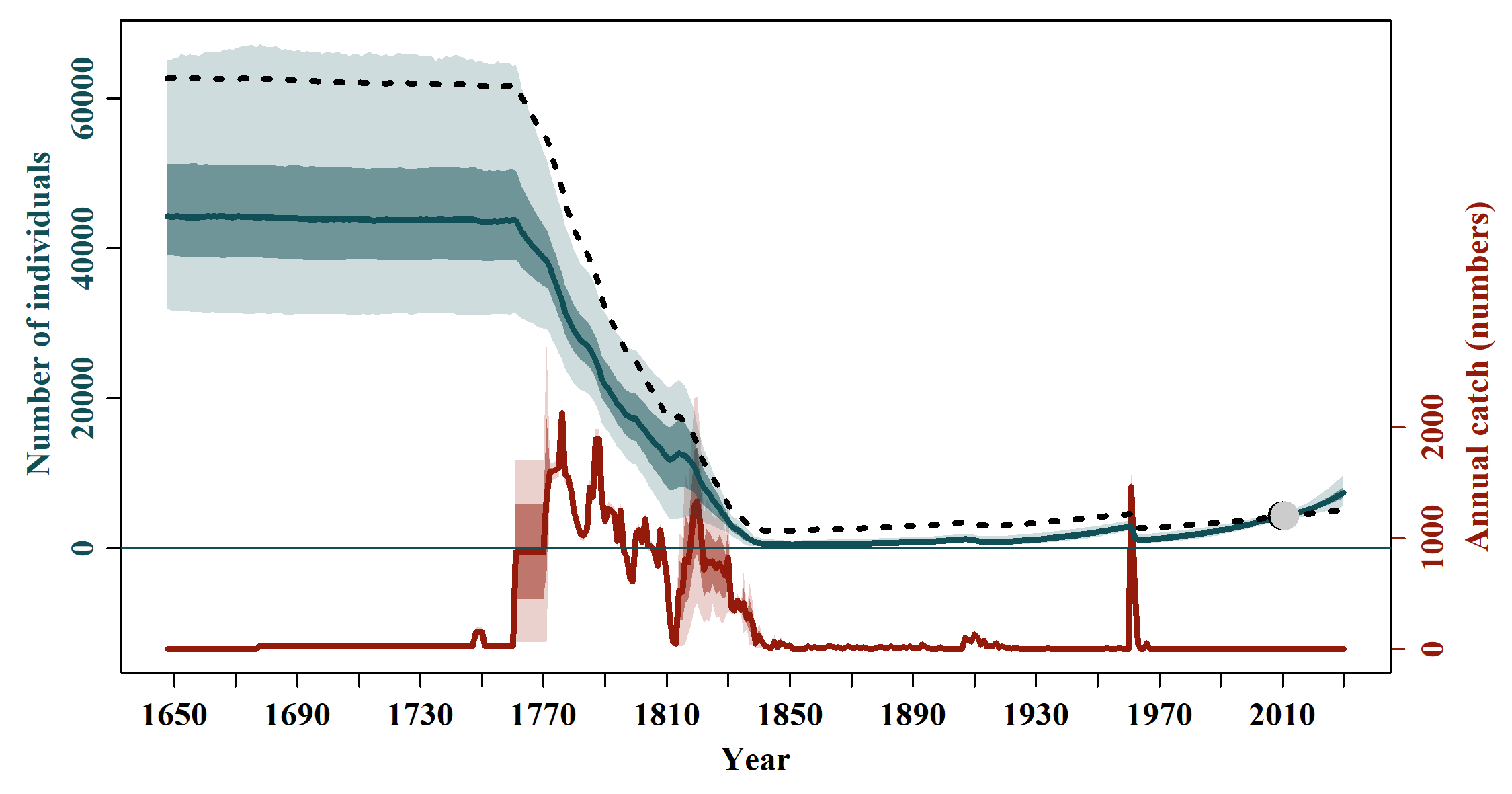


S3.4 Scen 3


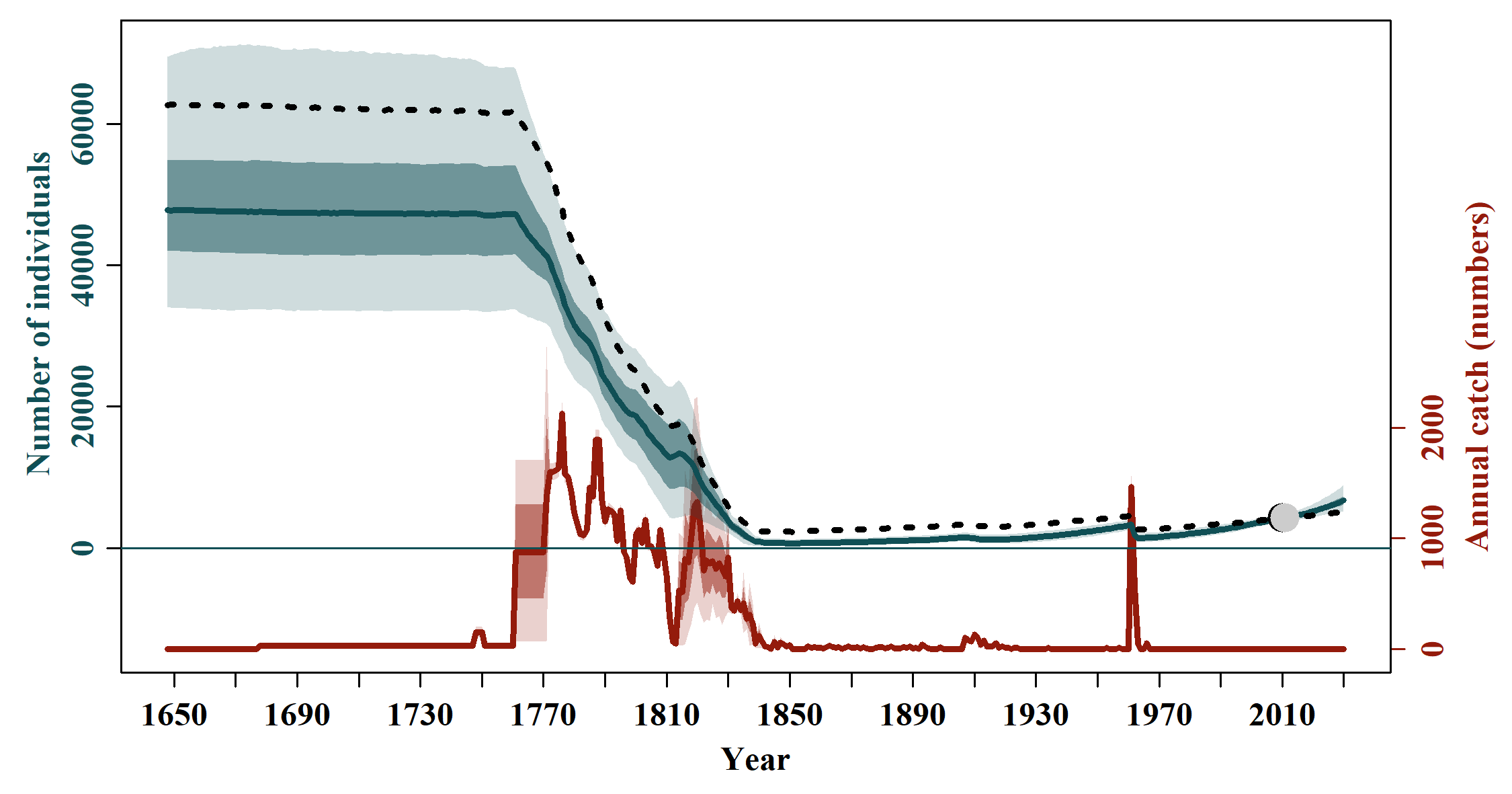


S3.5 Scen 4


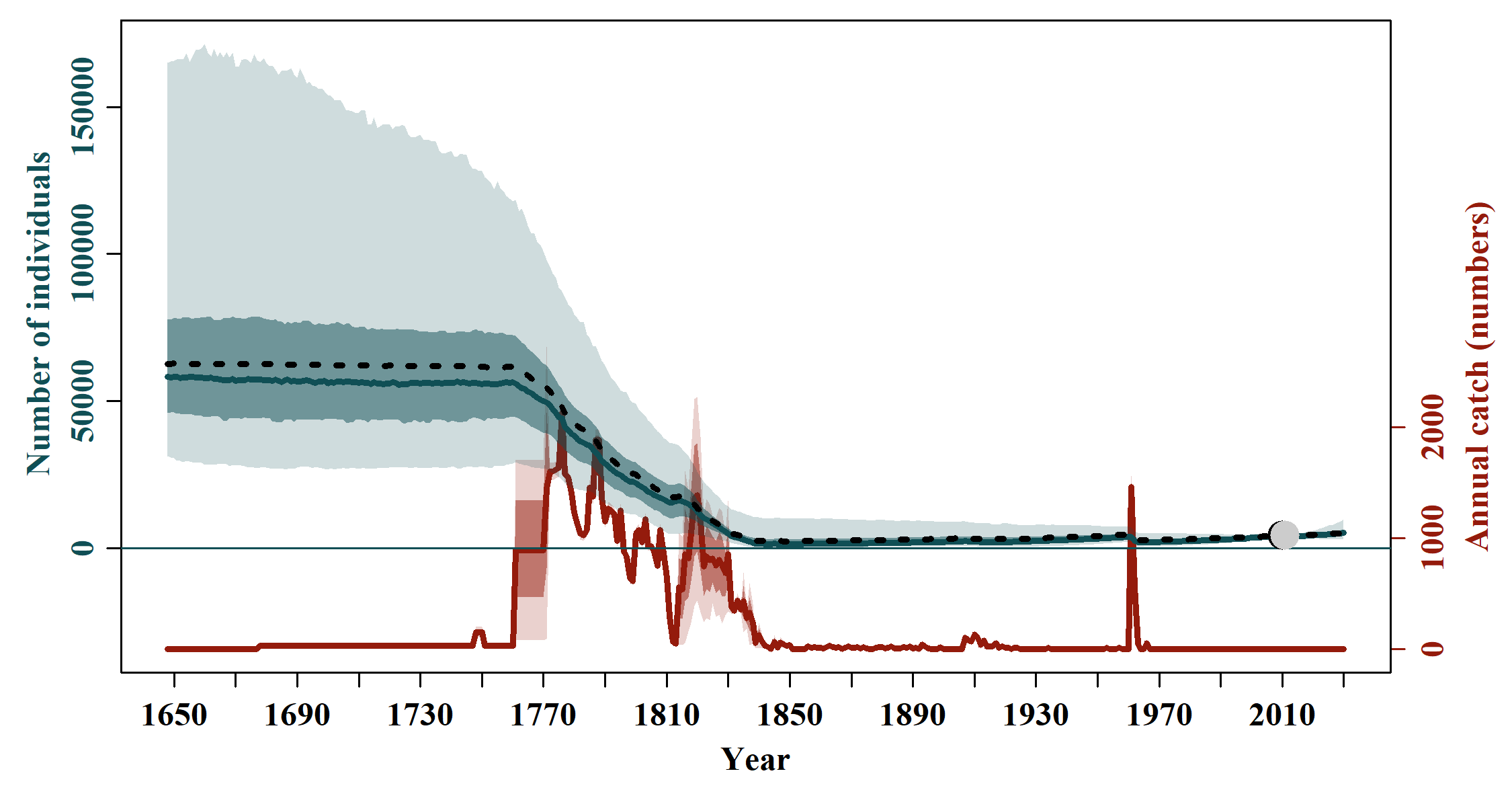


S3.6 Scen 5


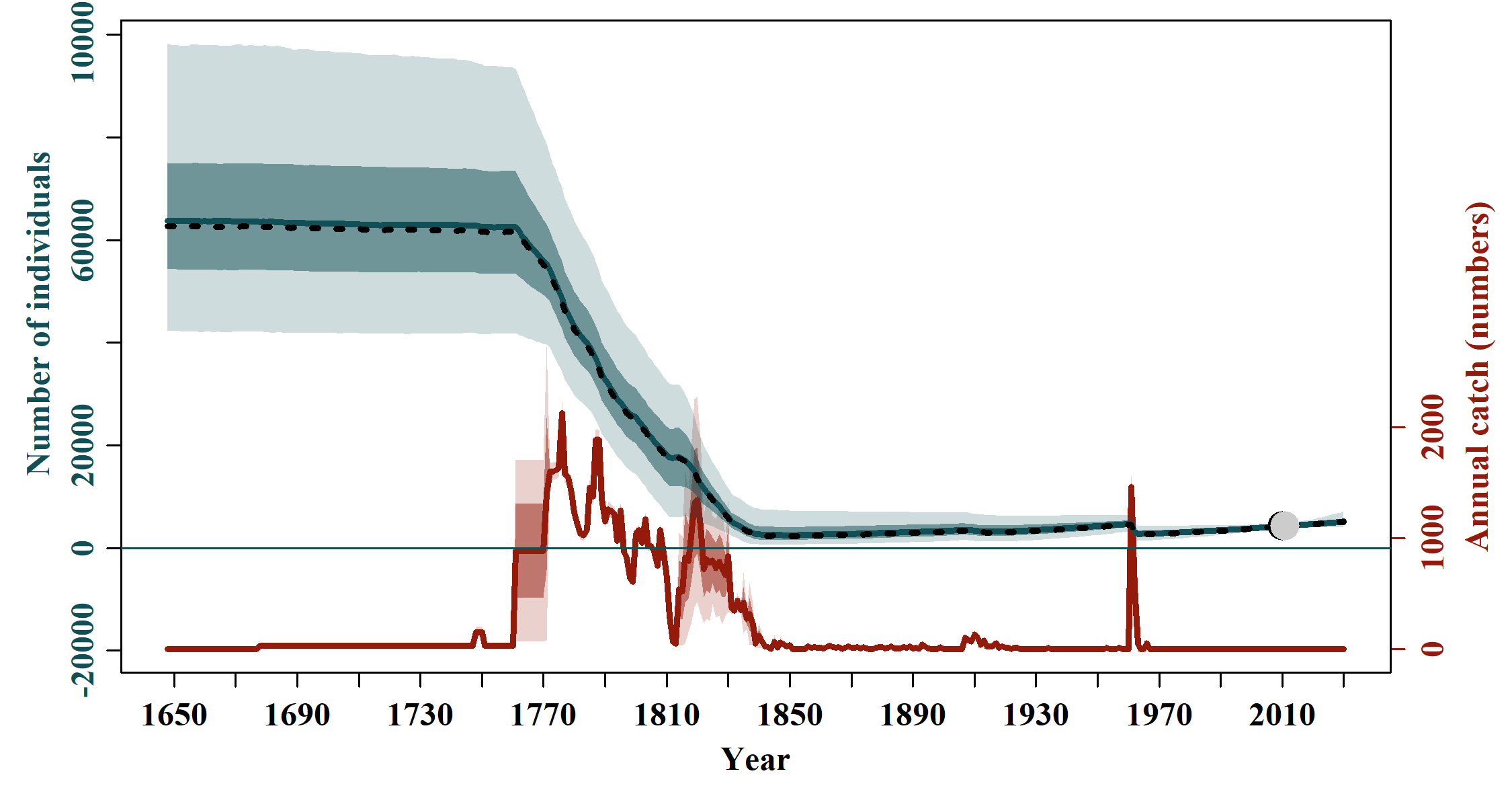


S3.7 Scen 6


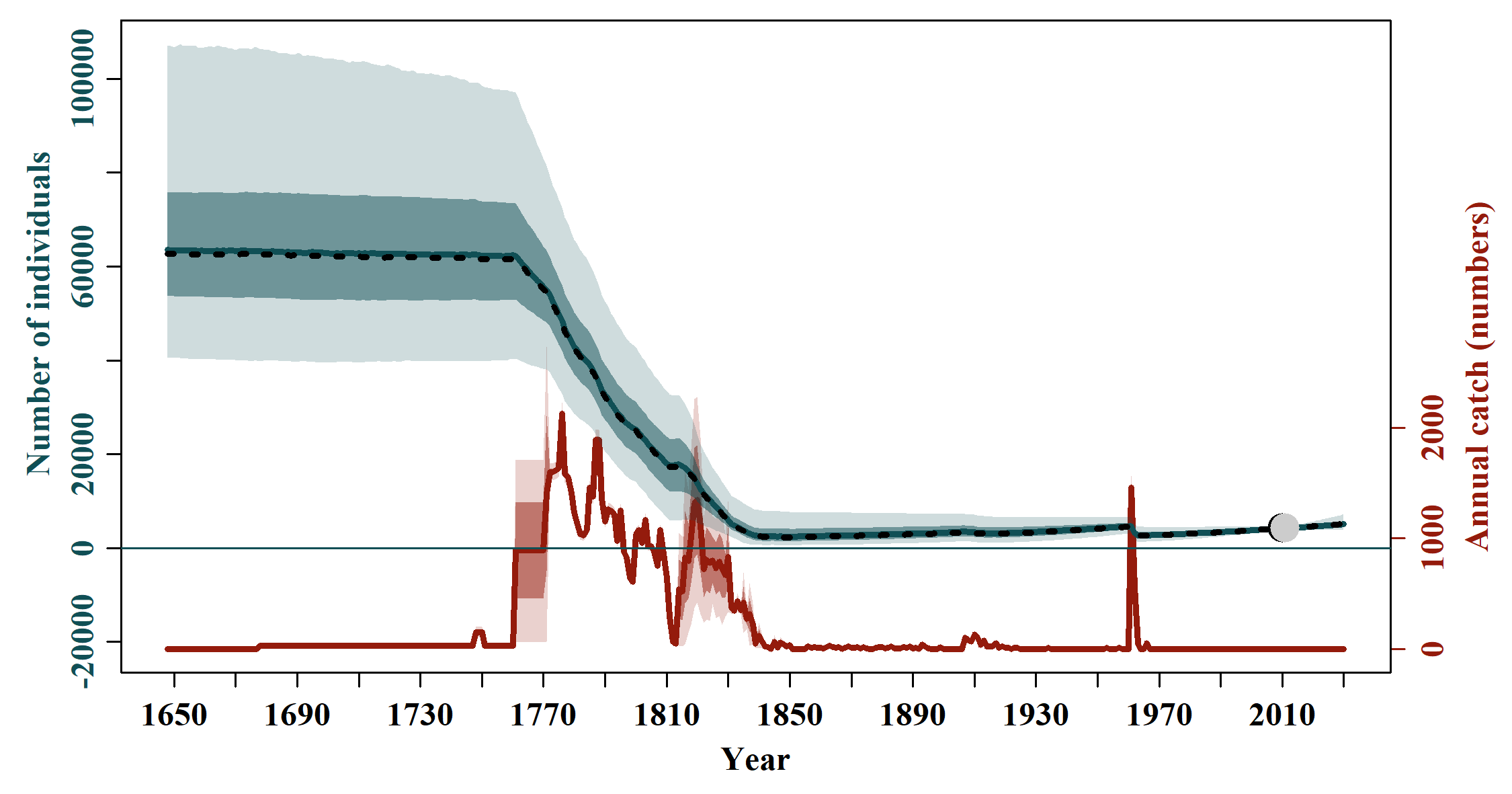


S3.8 Scen 7


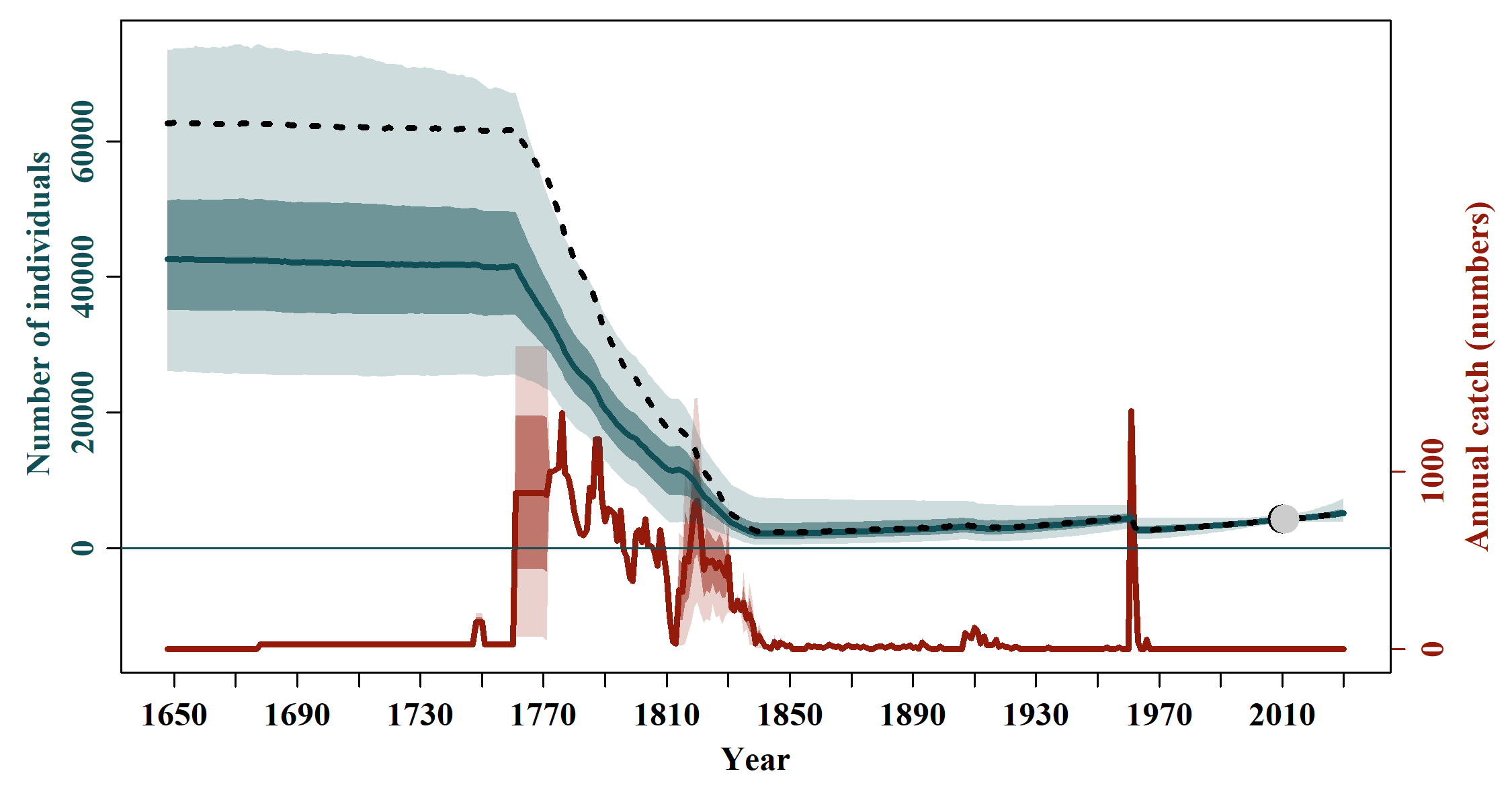


S3.9 Scen 8


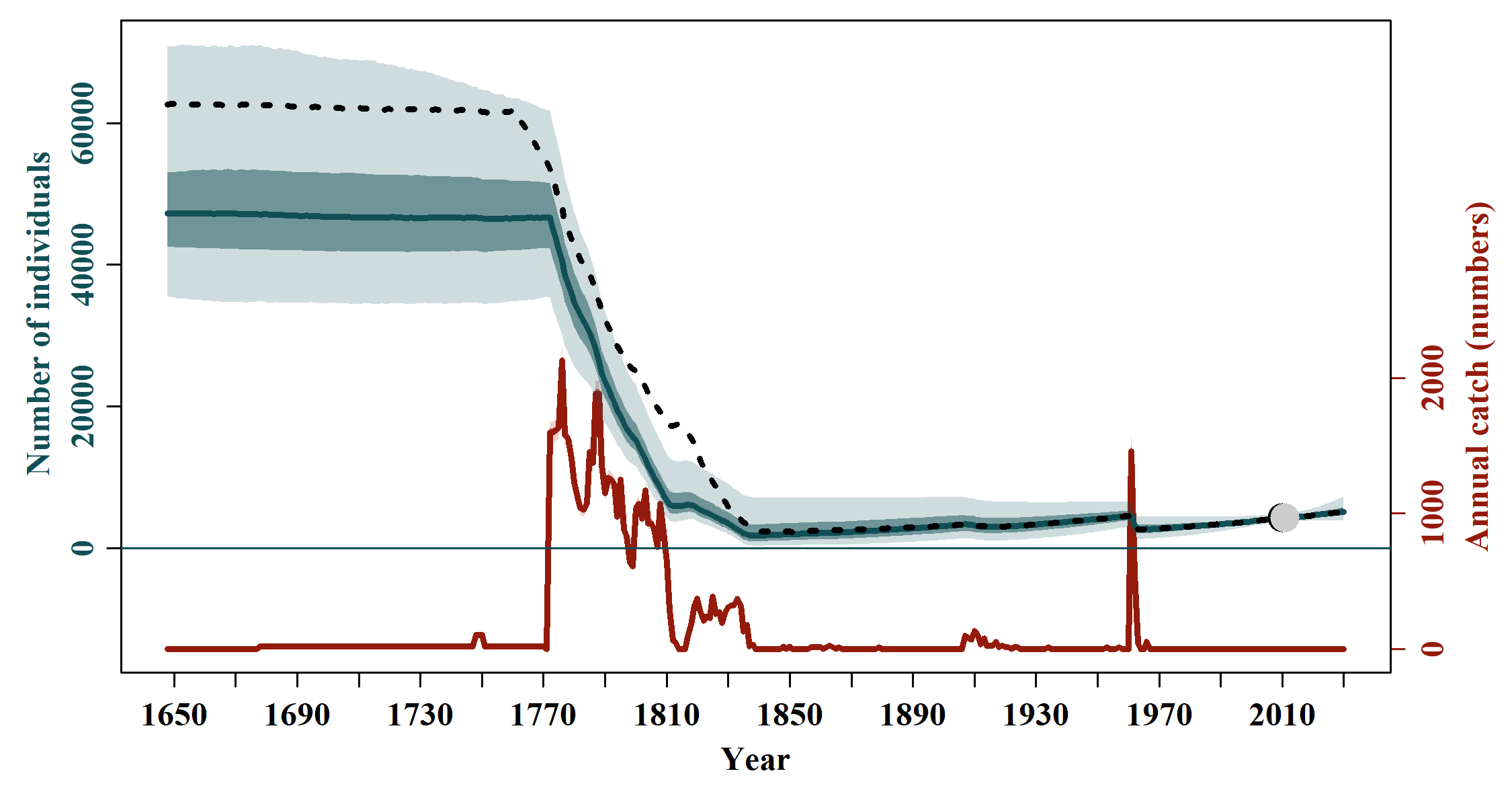


S3.10 Scen 9


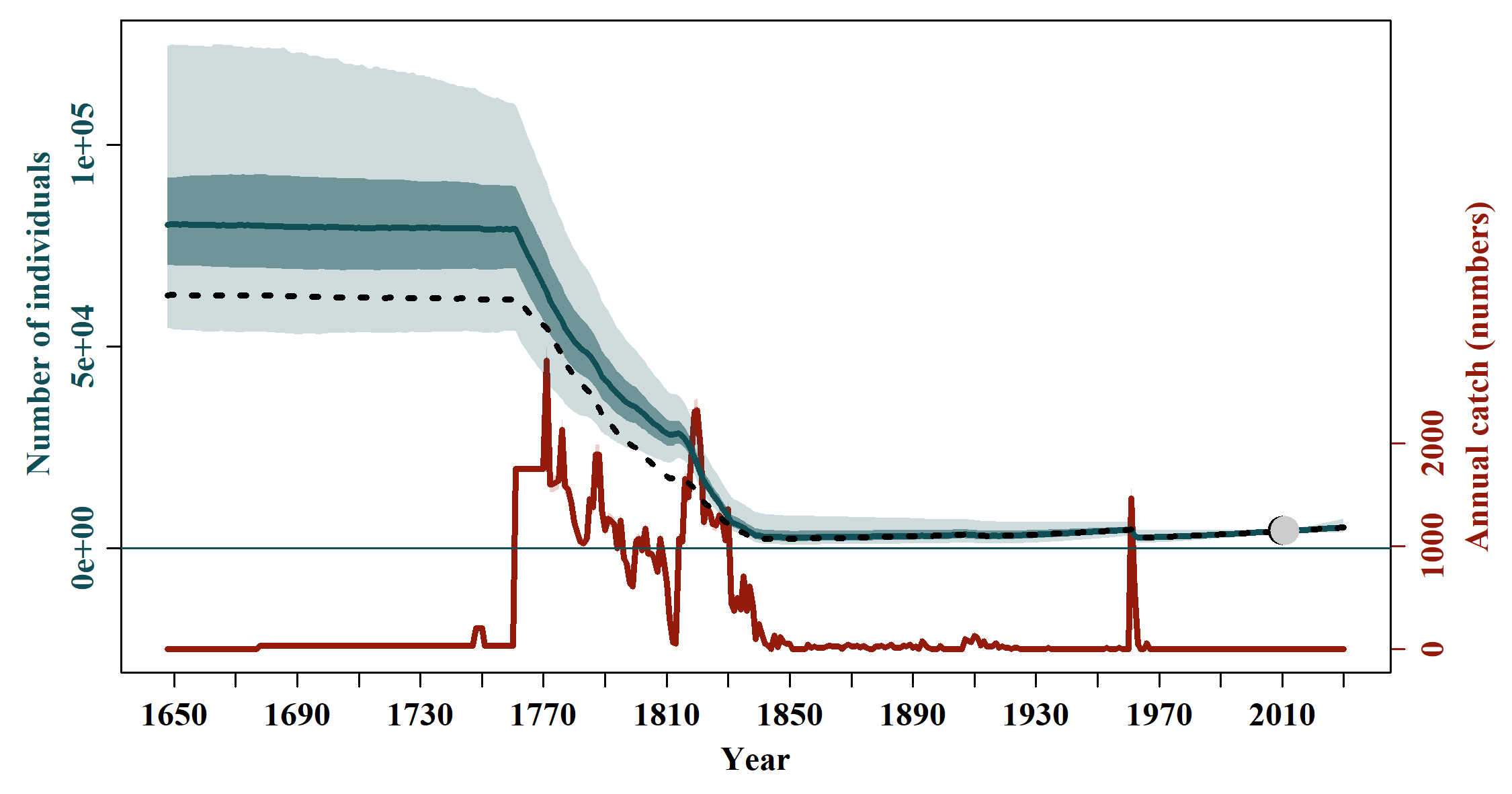


S3.11 Scen 10


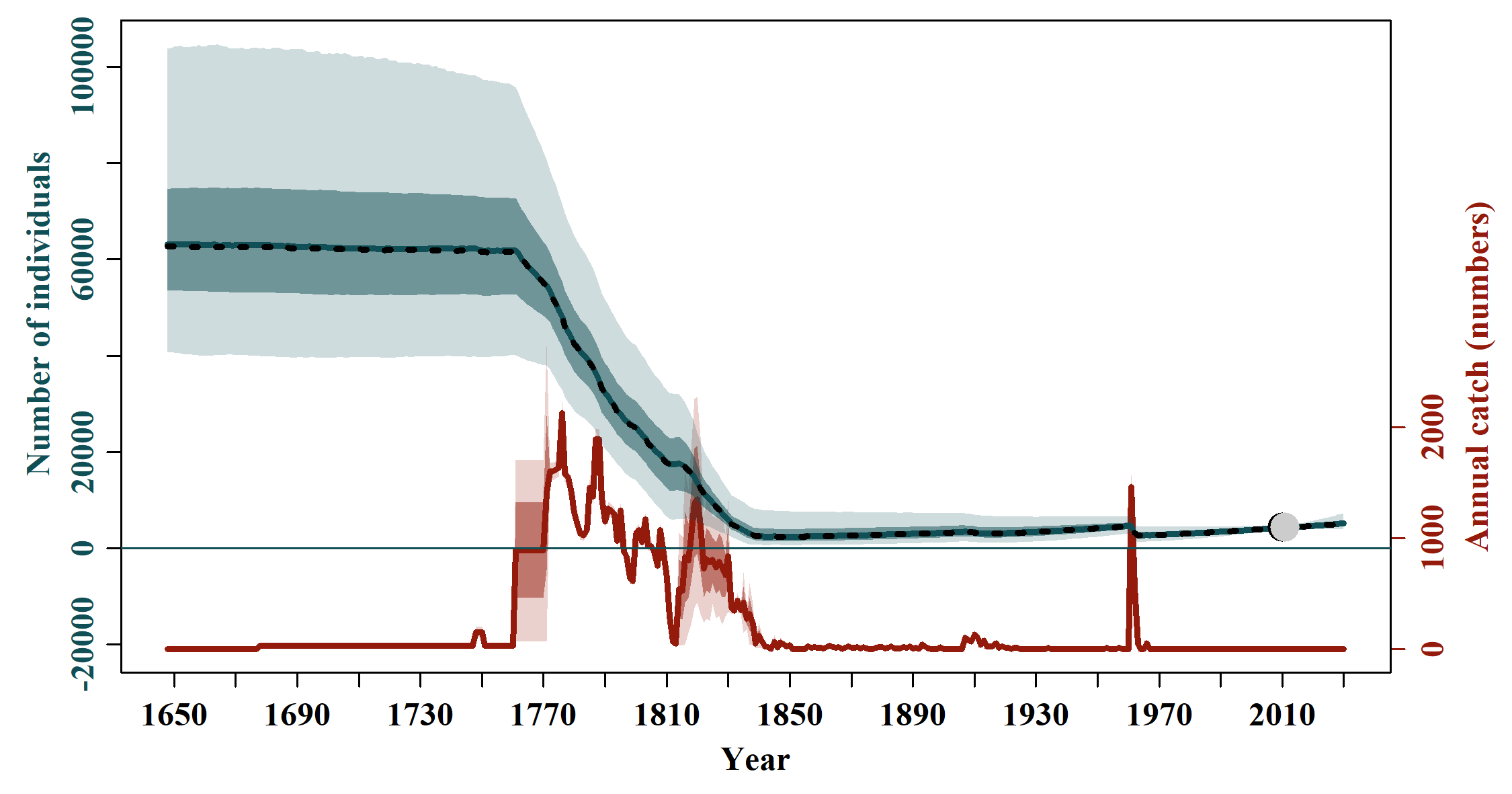


S3.12 Scen 11


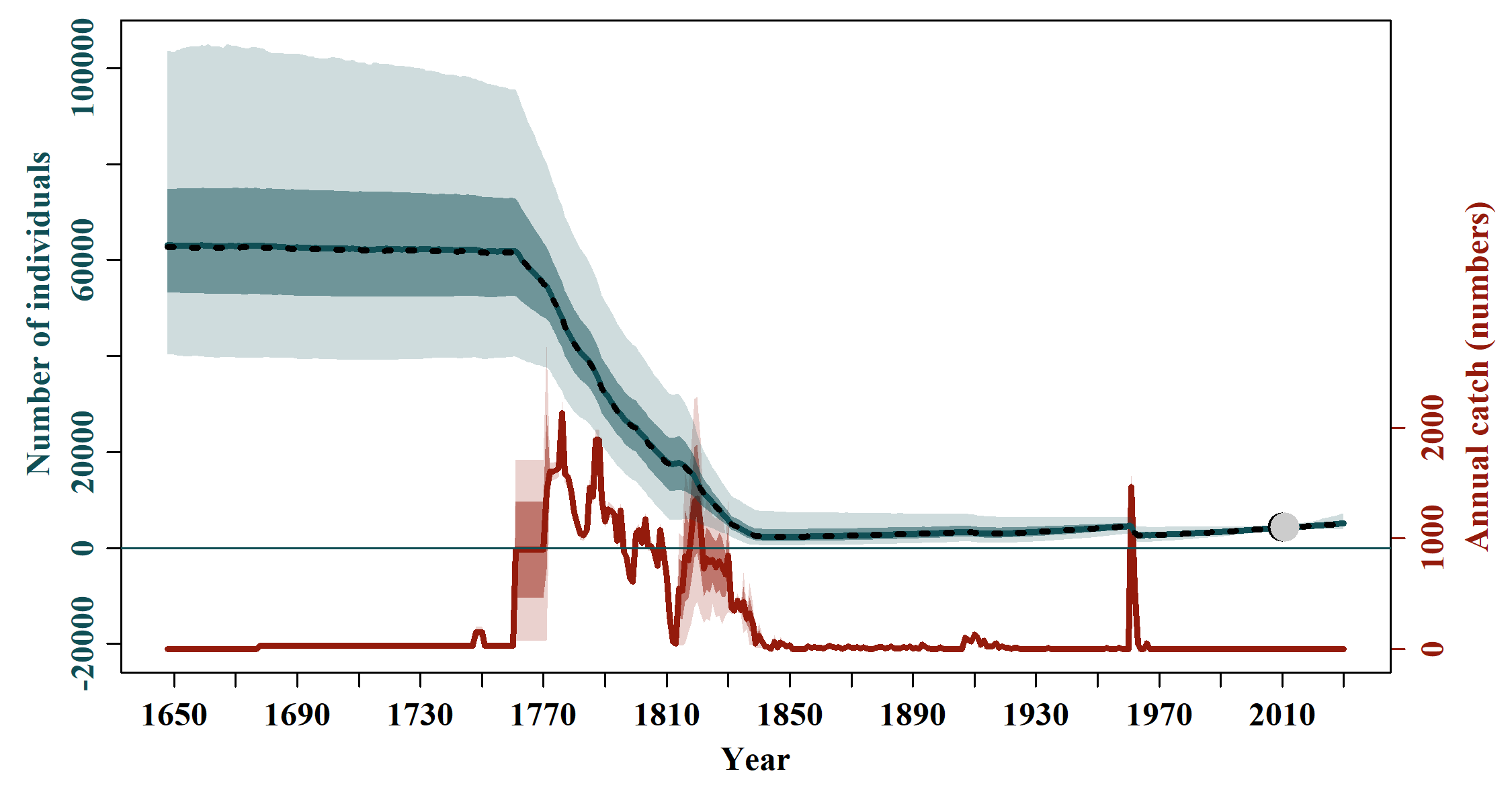


S3.13 Scen 12


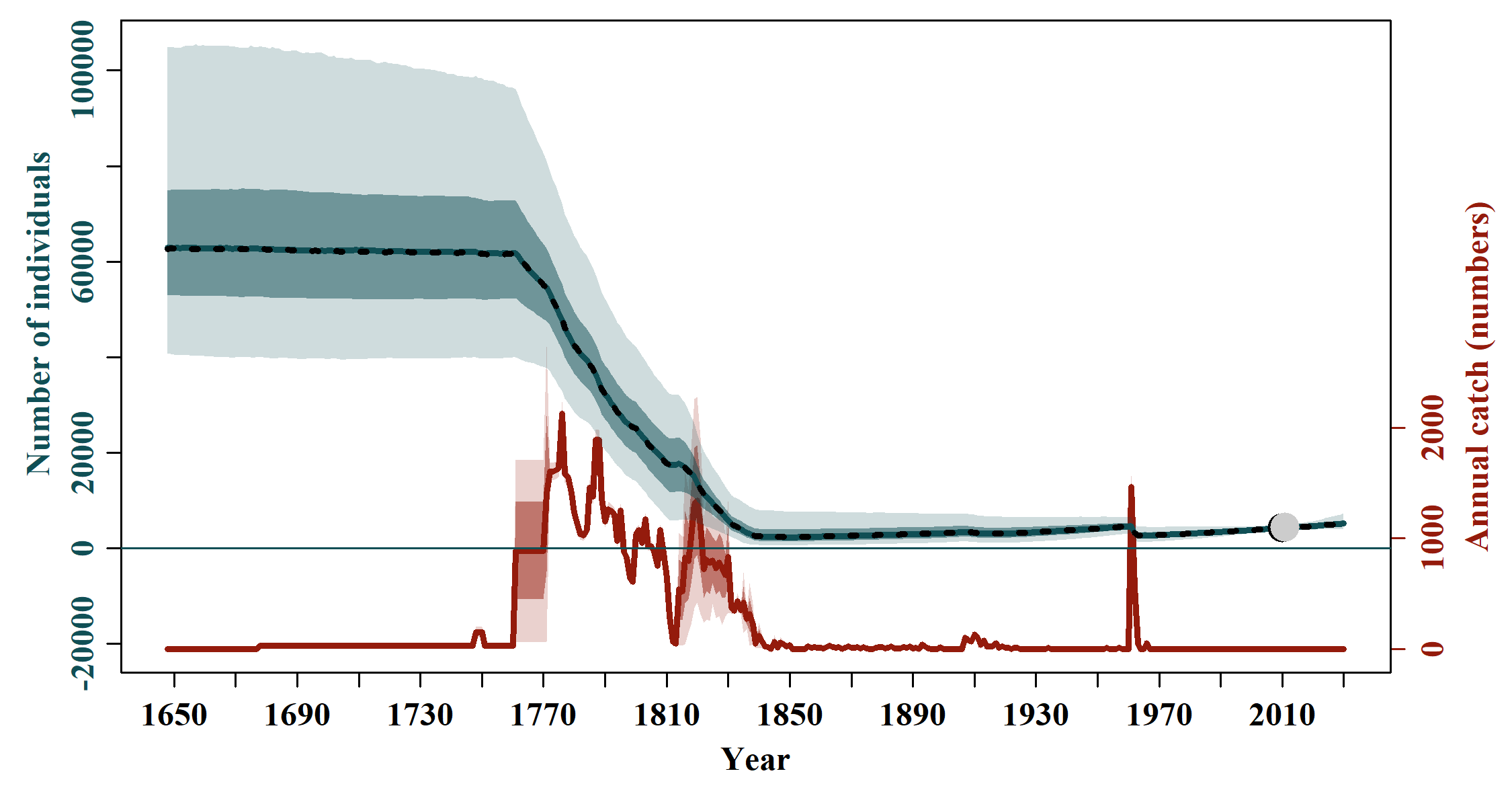


S3.14 Scen 13


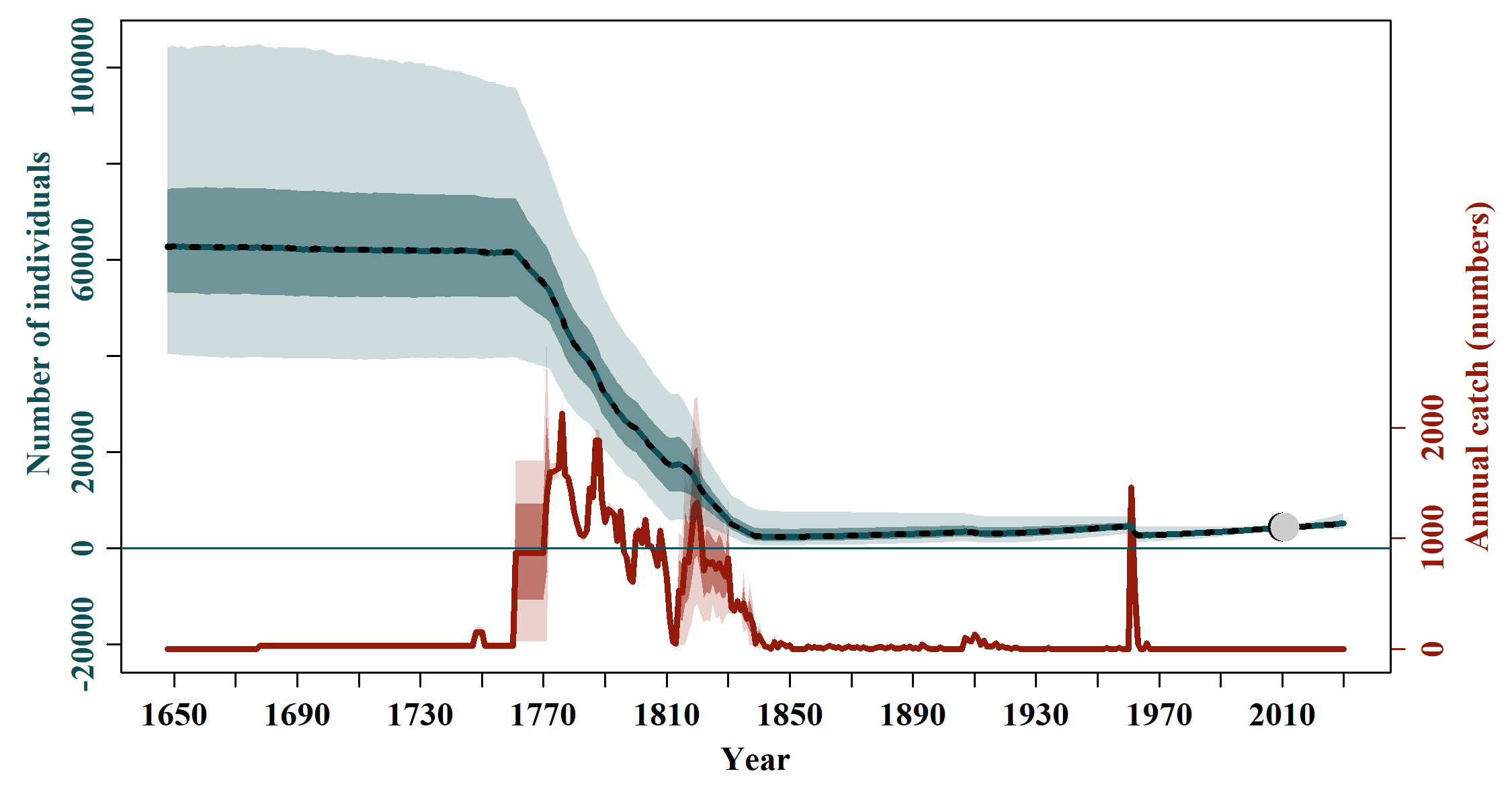


S3.15 Scen 14


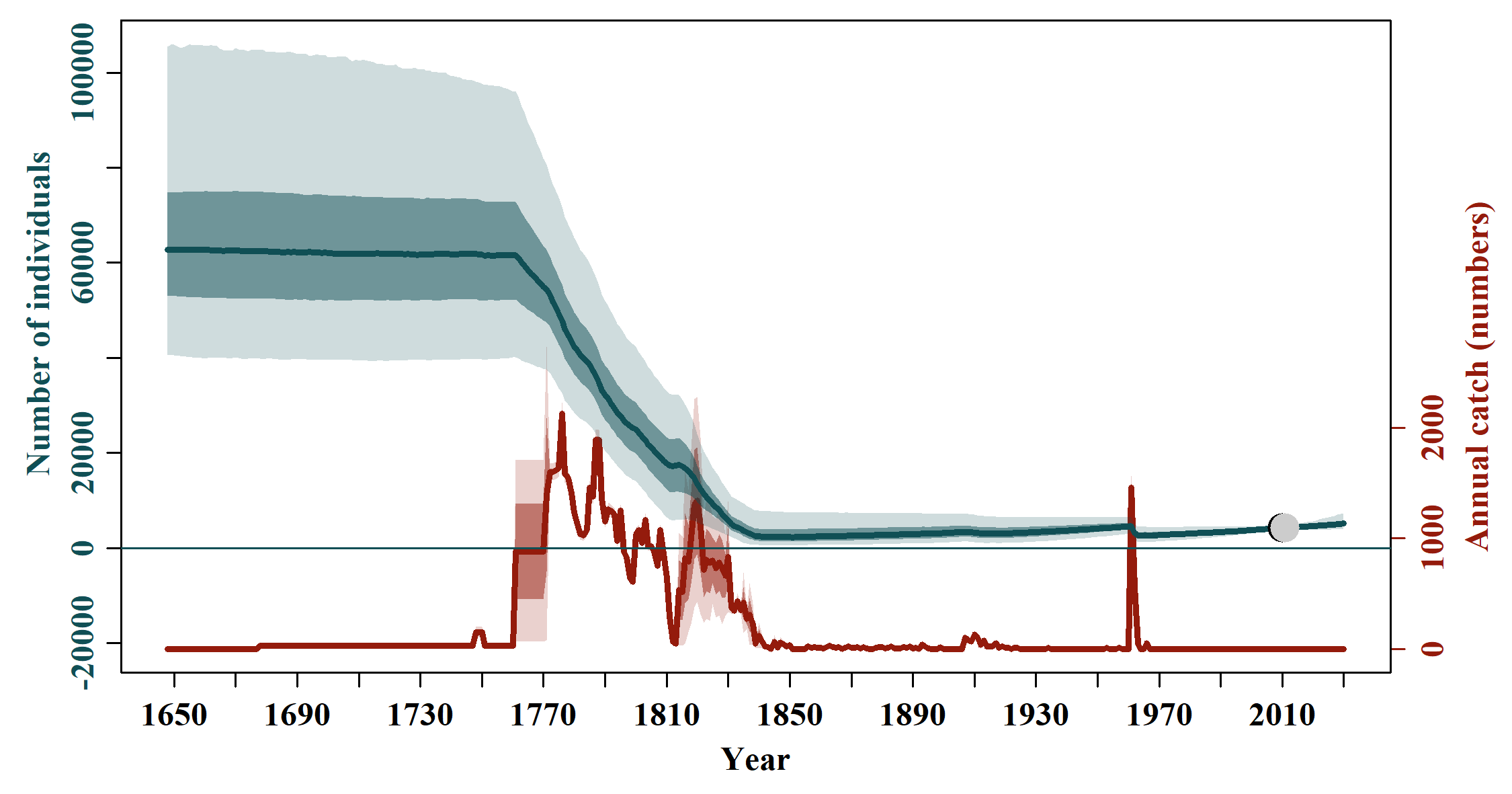


Figure S4. Fits of the observed (black dots) and estimated (grey dots) absolute abundance of southern right whale *Eubalaena australis* and associated 95% confidence interval (black bars) and 95% posterior predictive intervals (grey bars). The blue line is the median abundance trajectory and the shaded areas correspond to the 50% and 95% credible intervals.

| S4.0 Model average  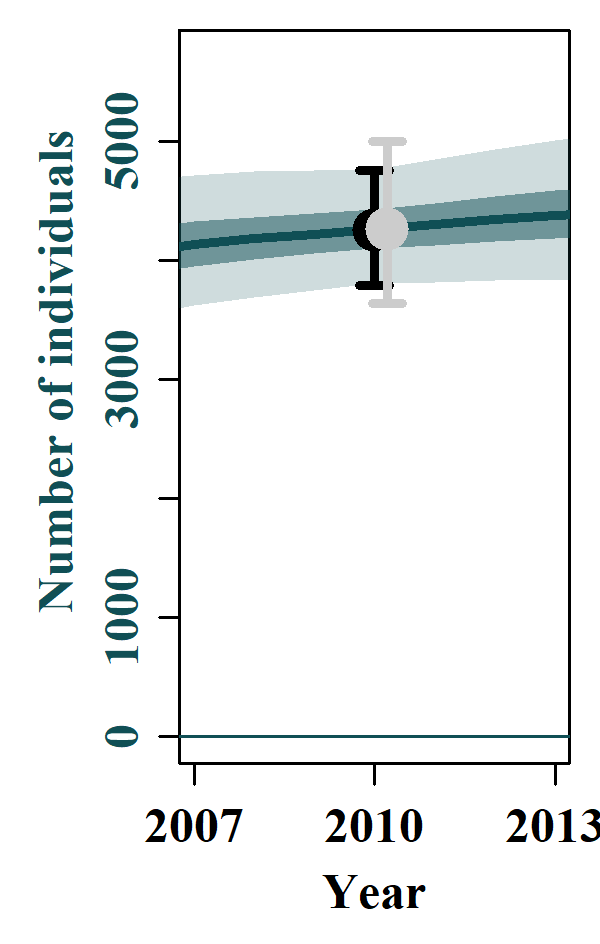 | S4.1 Base case  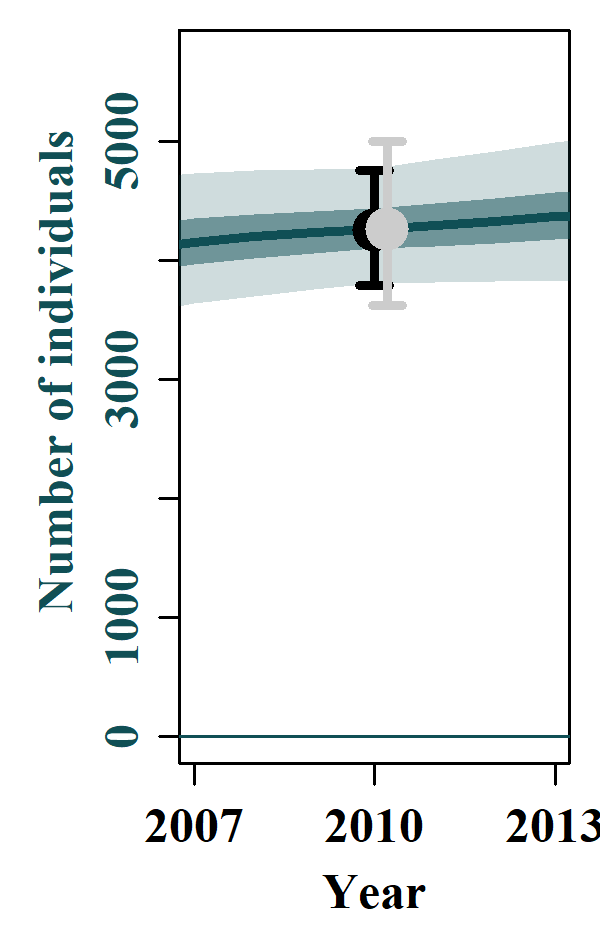 | S4.2 Scen 1  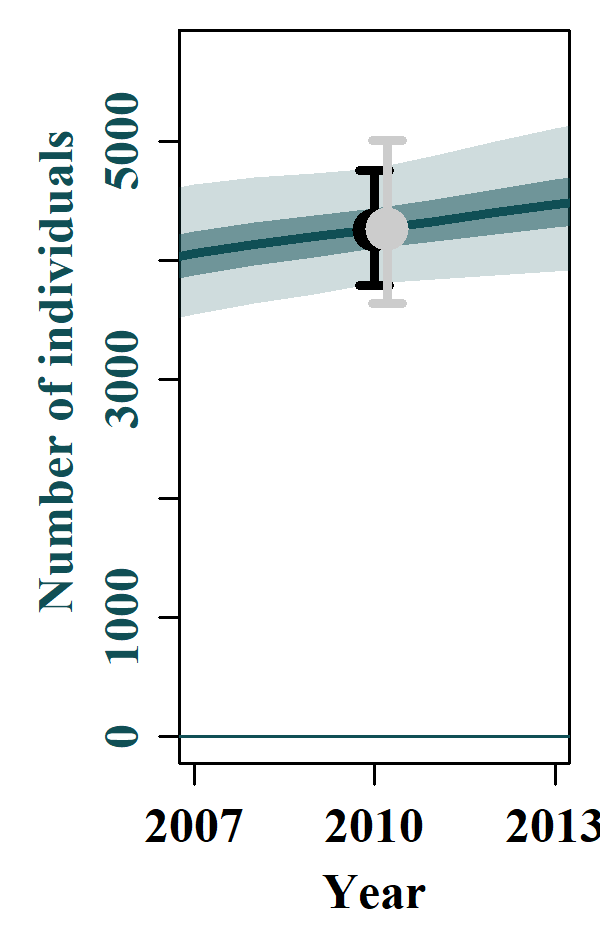 | S4.3 Scen 2  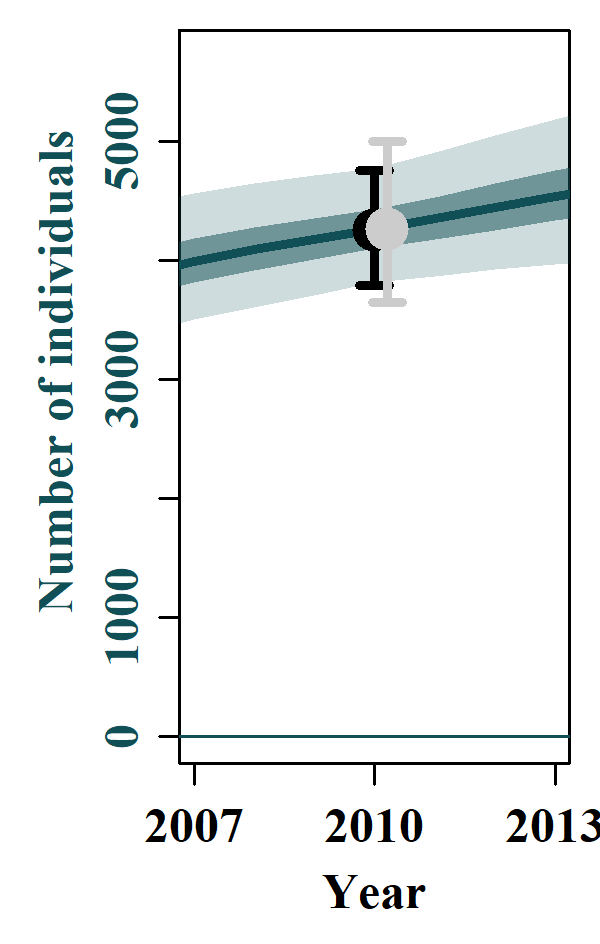 |
| --- | --- | --- | --- |
| S4.4 Scen 3  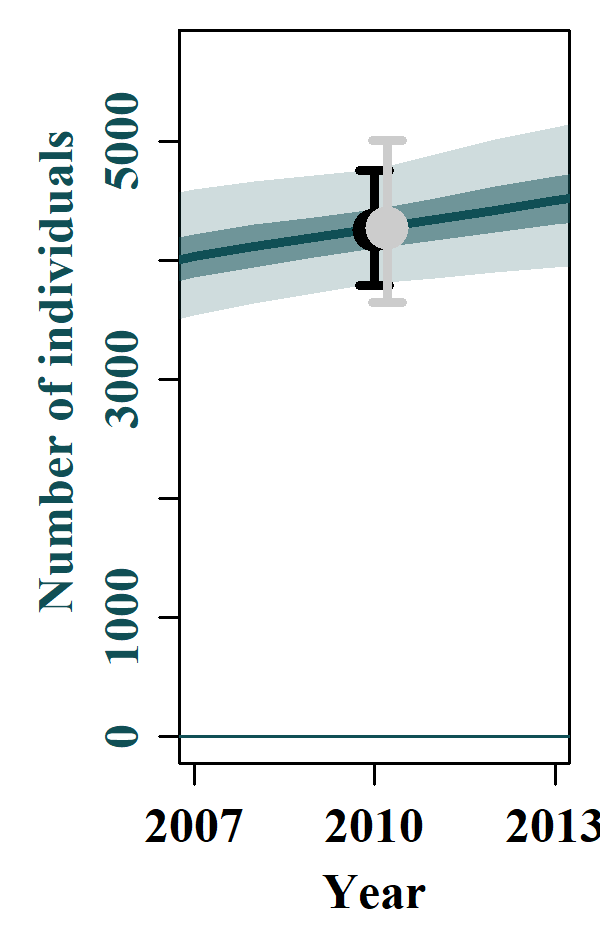 | S4.5 Scen 4  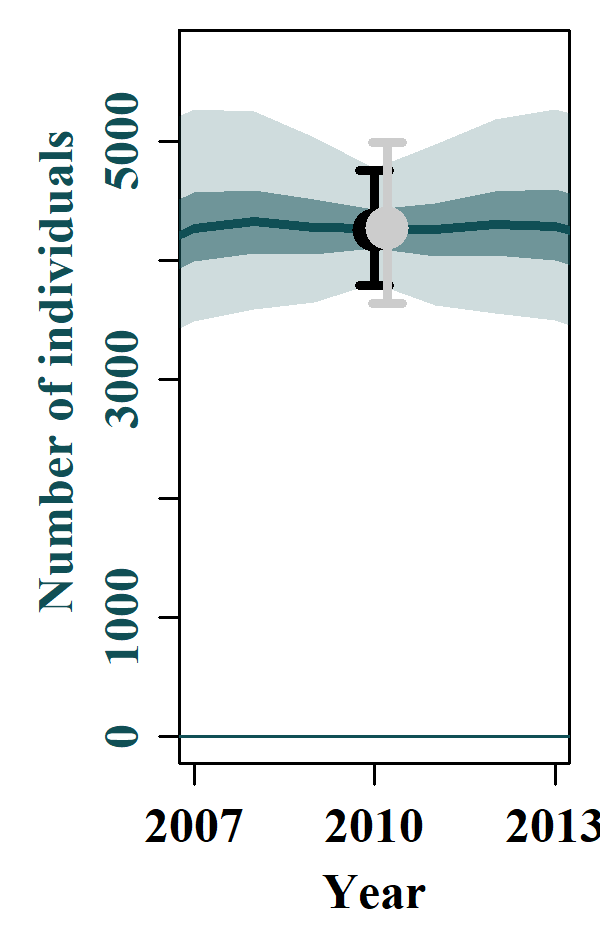 | S4.6 Scen 5  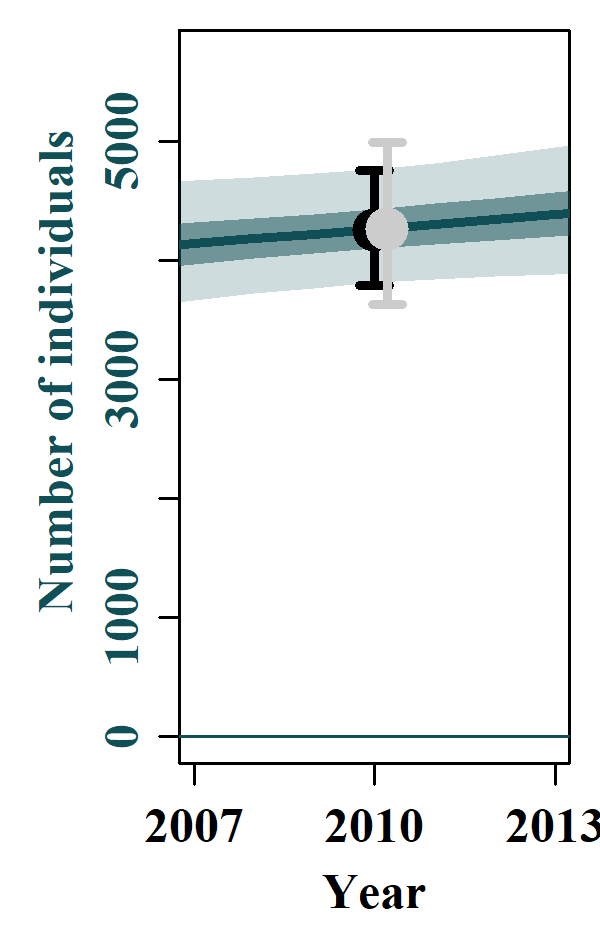 | S4.7 Scen 6  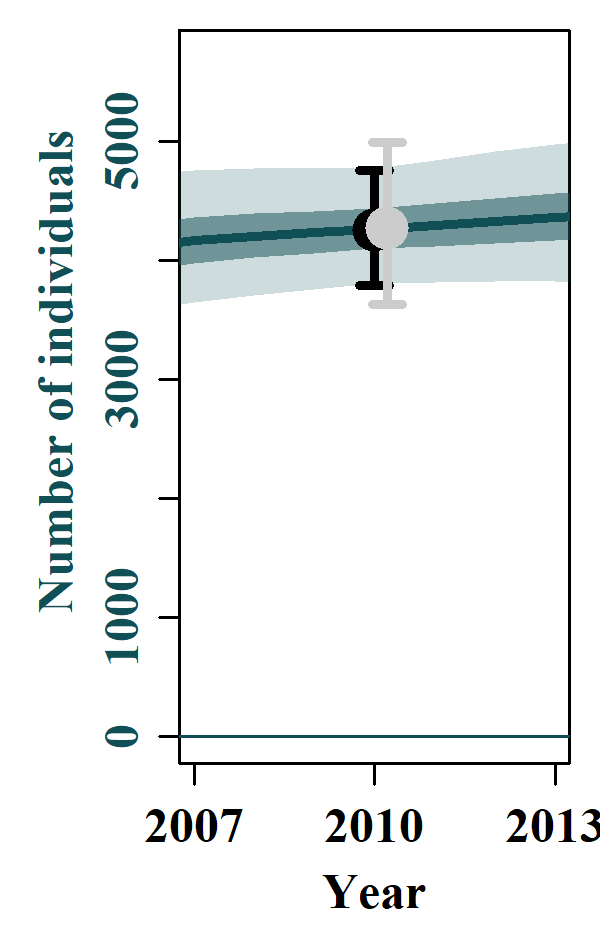 |
| S4.8 Scen 7  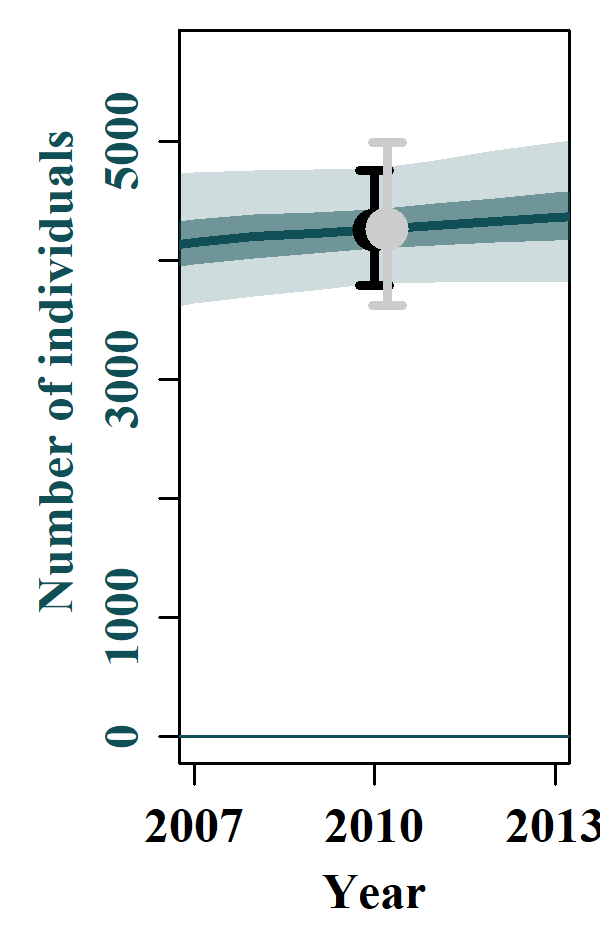 | S4.9 Scen 8  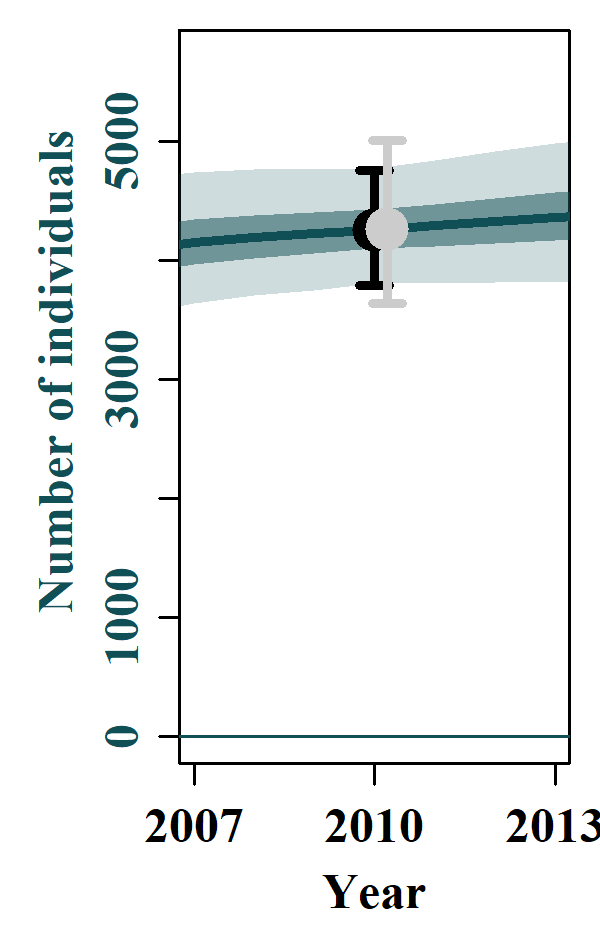 | S4.10 Scen 9  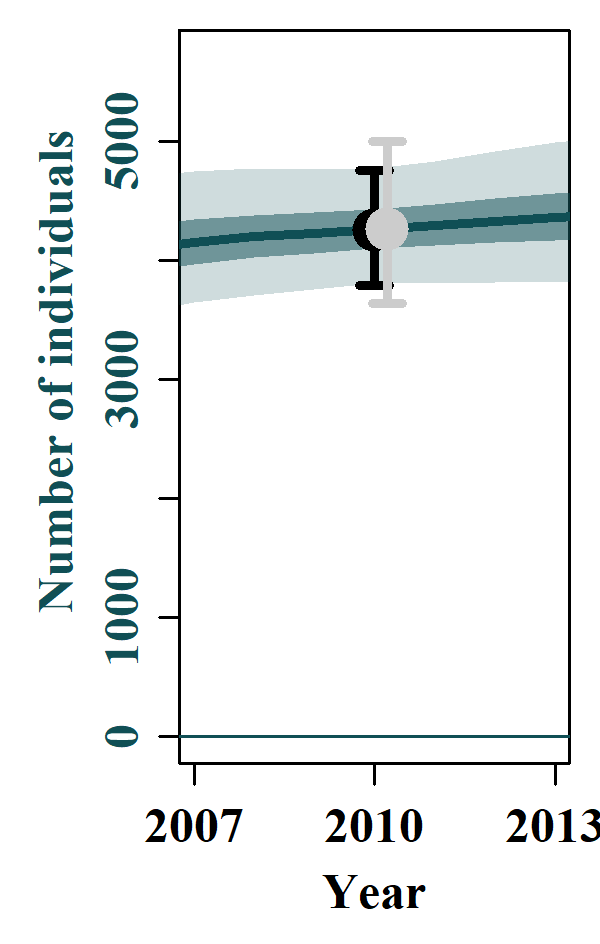 | S4.11 Scen 10  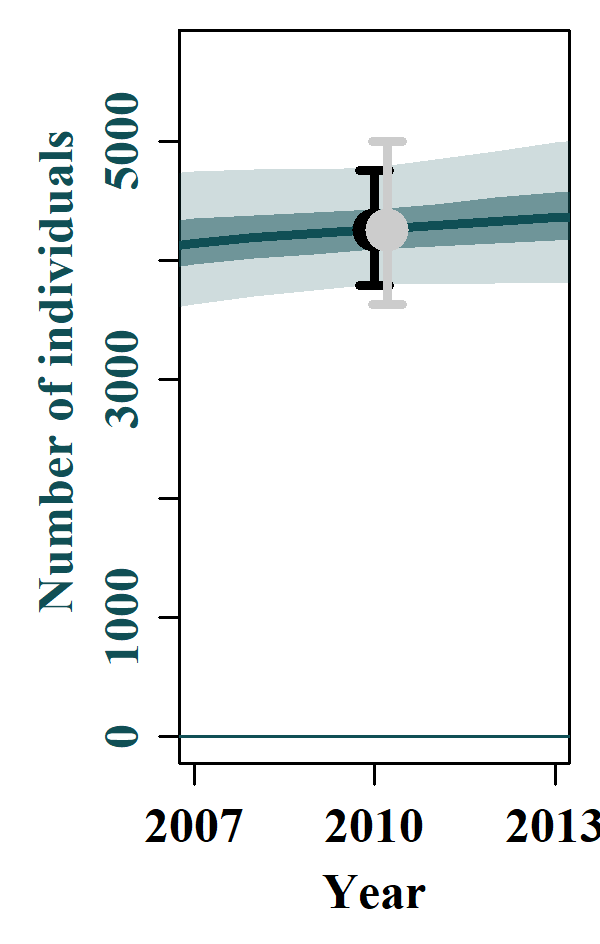 |
| S4.12 Scen 11  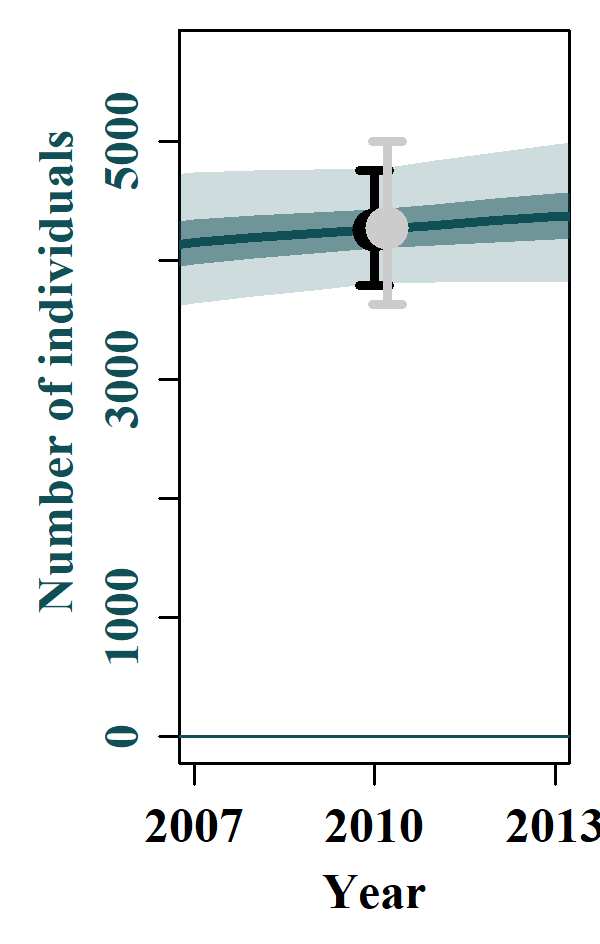 | S4.13 Scen 12  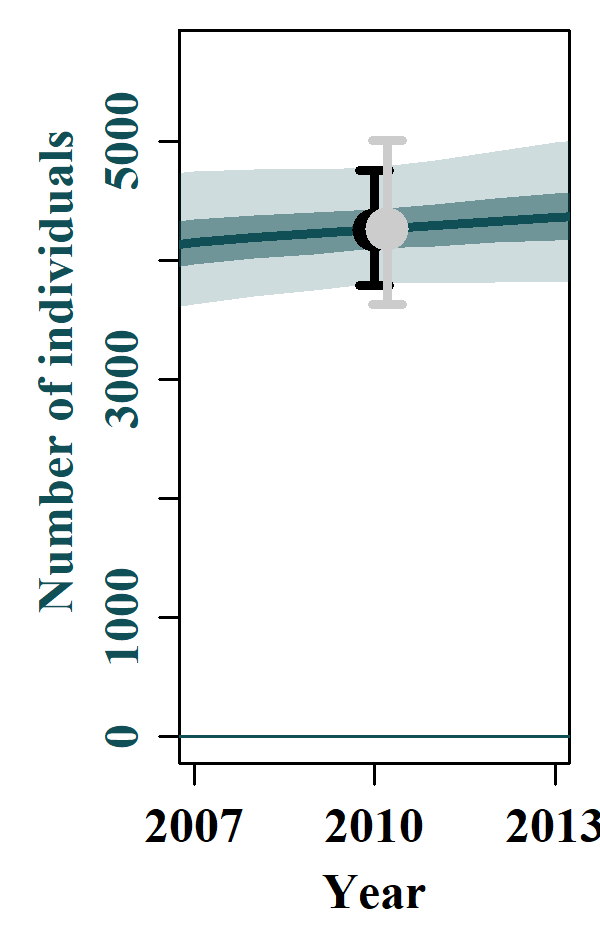 | S4.14 Scen 13  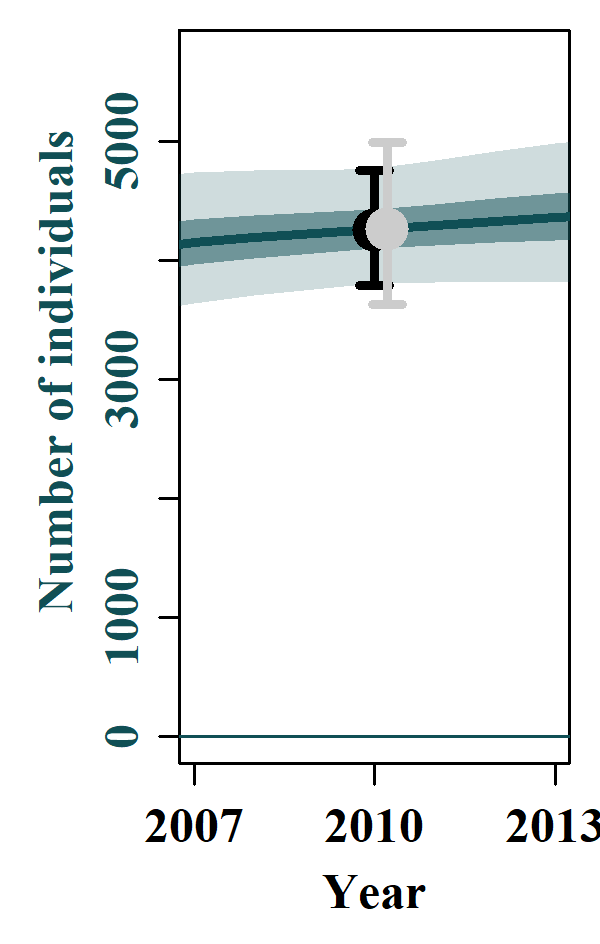 | S4.15 Scen 14  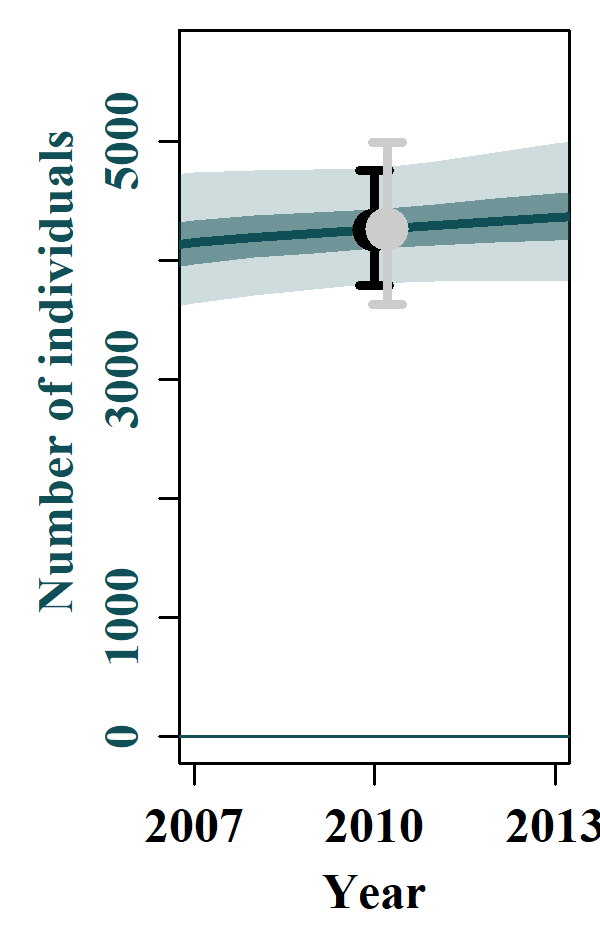 |
